# Conservative and Atypical Ferritins of Sponges

**DOI:** 10.3390/ijms22168635

**Published:** 2021-08-11

**Authors:** Kim I. Adameyko, Anton V. Burakov, Alexander D. Finoshin, Kirill V. Mikhailov, Oksana I. Kravchuk, Olga S. Kozlova, Nicolay G. Gornostaev, Alexander V. Cherkasov, Pavel A. Erokhov, Maria I. Indeykina, Anna E. Bugrova, Alexey S. Kononikhin, Andrey V. Moiseenko, Olga S. Sokolova, Artem N. Bonchuk, Irina V. Zhegalova, Anton A. Georgiev, Victor S. Mikhailov, Natalia E. Gogoleva, Guzel R. Gazizova, Elena I. Shagimardanova, Oleg A. Gusev, Yulia V. Lyupina

**Affiliations:** 1 N.K. Koltzov Institute of Developmental Biology, Russian Academy of Sciences, 119334 Moscow, Russia; kim.adameyko@idbras.ru (K.I.A.); finoshin@idbras.ru (A.D.F.); kravchuk444@mail.ru (O.I.K.); n_gornostaev@mail.ru (N.G.G.); paer@freemail.ru (P.A.E.); mikhailov48@mail.ru (V.S.M.); 2 A.N. Belozersky Institute of Physical and Chemical Biology, Lomonosov Moscow State University, 119234 Moscow, Russia; antburakov@belozersky.msu.ru (A.V.B.); kv.mikhailov@gmail.com (K.V.M.); 3 A.A. Kharkevich Institute for Information Transmission Problems, Russian Academy of Sciences, 127051 Moscow, Russia; I.Zhegalova@skoltech.ru; 4 Extreme Biology Laboratory, Institute of Fundamental Medicine and Biology, Kazan Federal University, 420111 Kazan, Russia; olga-sphinx@yandex.ru (O.S.K.); NEGogoleva@kpfu.ru (N.E.G.); grgazizova@gmail.com (G.R.G.); rjuka@mail.ru (E.I.S.); oleg.gusev@riken.jp (O.A.G.); 5 Center of Life Sciences, Skolkovo Institute of Science and Technology, 143026 Moscow, Russia; A.Cherkasov@skoltech.ru; 6 N.M. Emanuel Institute of Biochemical Physics, Russian Academy of Sciences, 119334 Moscow, Russia; mariind@yandex.ru (M.I.I.); anna.bugrova@gmail.com (A.E.B.); 7 Center for Computational and Data-Intensive Science and Engineering, Skolkovo Institute of Science and Technology, 143026 Moscow, Russia; konoleha@yandex.ru; 8 Faculty of Biology, M.V. Lomonosov Moscow State University, 119991 Moscow, Russia; postmoiseenko@gmail.com (A.V.M.); sokolova@mail.bio.msu.ru (O.S.S.); semga2001@yandex.ru (A.A.G.); 9 Institute of Gene Biology, Russian Academy of Sciences, 119334 Moscow, Russia; errinaceus@rambler.ru; 10 Faculty of Bioengineering and Bioinformatics, M.V. Lomonosov Moscow State University, 119991 Moscow, Russia; 11 Department of Regulatory Transcriptomics for Medical Genetic Diagnostics, Graduate School of Medical Sciences, Juntendo University, Tokyo 113-8421, Japan; 12 RIKEN Center for Integrative Medical Sciences, RIKEN, Yokohama 351-0198, Japan

**Keywords:** ferritin, heme, globins, iron, sponges, *Halisarca dujardini*, *Halichondria panicea*, invertebrates, whole body regeneration

## Abstract

Ferritins comprise a conservative family of proteins found in all species and play an essential role in resistance to redox stress, immune response, and cell differentiation. Sponges (Porifera) are the oldest Metazoa that show unique plasticity and regenerative potential. Here, we characterize the ferritins of two cold-water sponges using proteomics, spectral microscopy, and bioinformatic analysis. The recently duplicated conservative *HdF1a/b* and atypical *HdF2* genes were found in the *Halisarca dujardini* genome. Multiple related transcripts of *HpF1* were identified in the *Halichondria panicea* transcriptome. Expression of *HdF1a/b* was much higher than that of *HdF2* in all annual seasons and regulated differently during the sponge dissociation/reaggregation. The presence of the MRE and HRE motifs in the *HdF1* and *HdF2* promotor regions and the IRE motif in mRNAs of *HdF1* and *HpF* indicates that sponge ferritins expression depends on the cellular iron and oxygen levels. The gel electrophoresis combined with specific staining and mass spectrometry confirmed the presence of ferric ions and ferritins in multi-subunit complexes. The 3D modeling predicts the iron-binding capacity of HdF1 and HpF1 at the ferroxidase center and the absence of iron-binding in atypical HdF2. Interestingly, atypical ferritins lacking iron-binding capacity were found in genomes of many invertebrate species. Their function deserves further research.

## 1. Introduction

Regulation of iron availability is an important part of cellular homeostasis. The bioavailability of iron is limited by the insolubility of ferric salts, while the excess of free iron leads to oxidative stress. Sequestration of iron ions within cells is mediated by ferritins, a widely distributed and conserved protein family found in all domains of life. Ferritins are organized in complexes consisting of 24 subunits forming a hollow sphere that is able to accumulate and store up to 4500 Fe^3+^ atoms as ferrihydrite and, thereby, serve as a buffer to sequester excessive iron [1]. When iron ions are needed, lysosomal degradation leads to their release from the ferritin complexes. Mammalian ferritin is composed of heavy (H) and light (L) chains. The H subunits are responsible for rapid detoxification of iron by ferroxidase activity [2,3], and the L subunits facilitate iron nucleation, mineralization, and long-term storage [4,5]. Members of the ferritin-like protein superfamily are involved in multiple cellular metabolic pathways, the redox-stress resistance, DNA replication, chlorophyll biosynthesis, endospore coat formation, fatty acid metabolism, tRNA modification, monooxygenase reactions, detoxification, and biomineralization [6,7,8]. Ferritins are used as delivery vehicles for iron and drugs, as well as biomarkers of various diseases, so the main important functions of ferritin have been studied in vertebrates. Knockout of the mouse H-ferritin (MoHF) is embryonically lethal, and inactivation of MoHF makes the cells, especially myeloid, more sensitive to oxidative damage [9,10,11]. Ferritin synthesis is stimulated during the development and cell differentiation, inflammation, and tumorigenesis. A decrease in H-ferritin can induce epithelial-to-mesenchymal transition of mammalian tumor cells through the TGF- β1 pathway [12,13,14]. Hypoxia inducible factor A (HIFA) can directly bind with hypoxia response element (HRE) in the promoter region of human L-ferritin (HuLF) to enhance its expression thus regulating epithelial-to-mesenchymal transition of glioma [15]. Cell death by ferroptosis is characterized by the iron-dependent accumulation of reactive oxidized lipid species and depends on the regulation of iron storage and ferritin expression [16]. Sensitivity to ferritinophagy and ferroptosis varies between different cell types. The resistance to ferroptosis is provided by the prominin2-MVB-exosome-ferritin pathway and has consequences for iron homeostasis and cancer [17]. The most specialized mammalian cells which are highly sensitive to iron deficiency and ferroptosis are the neural cells [18]. They utilize excessive iron under certain conditions (magnetic resonance imaging) faster than other body cells [19]. Invertebrate ferritins perform some unique functions that are not seen in the ferritins found in vertebrates. Ferritin of *Apis mellifera* (Honeybees) participates in magnetite formation in their trophocytes [20]. The ferritin-like superfamily proteins are associated with immune functions in the marine invertebrates [21], and ferritin ChF of the marine tubeworm *Chaetopterus* sp. is involved in the production of bioluminescence in the secreted mucus [22]. The crystal structure of ferritin from the marine bivalve mollusk *Sinonovacula constricta* predicted the iron-binding sites in the 3-fold channel, ferroxidase center, and putative nucleation sites, similar to the mammalian ferritins [23]. Exploring the functions of ferritins in ancient animals is of special interest.

Sponges (phylum Porifera) are benthic animals, and likely represent the oldest phylum of the existing Metazoa. Sea sponges of the sub-tidal zone are well adapted to changes in the temperature and oxygen content and provide a unique model for studying the cell adaptation processes in animals [24]. The sea sponges grow only in the presence of iron ions [25]. Some bacterial symbionts use iron receptors or enzymatic systems to extract iron from sponges [26,27]. These interconnections are genetically inherited and could be activated by low iron concentrations in the environment [28]. The main component of the sponge body is an aquiferous system that undergoes numerous rearrangements in the course of the sponge life cycle, as well as due to constant changes in the environmental conditions [29,30,31]. The sponge body is represented by several types of cells that can be in different redox and metabolic cycle phases. The gradients of oxygen, metabolites, and proteins stimulate constant movements and transformation of cells. The sponge cells have the capacity to transition between multiple cell types similar to the transdifferentiating and stem cells of mammals [32,33,34]. A unique feature of sponge cells is the ability to reaggregate and form functional primmorphs from dissociated cells. The experimental model of the sponge cells reaggregation was first introduced by Wilson in 1907 [35] to study morphological transitions in sponges and lately was exploited in many laboratories [35,36,37]. It has been demonstrated that *Halisarca* *dujardini* (cl. Demospongia) has a high regenerative capacity that combines protective and regenerative mechanisms [38,39]. By using biochemical methods and transcriptomic analyses, we have previously described iron metabolic and heme biosynthesis/transport pathways and their association with the reaggregation process in two cold-water sea sponges of cl. Demospongia, *Halisarca dujardini* (lacking spicules) and *Halichondria panicea* (having spicules) [24,40]. In the present study, we analyze ferritin complexes of these two sponge species, along with their regulation at transcriptional/translational levels, and their contribution to the adaptation processes. To elucidate the latter, differential expression of ferritins and related proteins of iron metabolism was studied in the sponge *H. dujardini* at different periods of the life cycle and in the course of morphogenetic processes accompanying sponge body dissociation and cells reaggregation.

## 2. Results

### 2.1. Ferritin Genes

In order to recover ferritins from studied sponges, we combined data from our published de novo transcriptomes for *Halisarca dujardini* and *Halichondria panicea* (NCBI project numbers PRJNA594150 and PRJNA594151) with draft genomic data for *H. dujardini* (Supplementary Table S1). Three ferritin sequences were identified in the transcriptomic data of *H. dujardini*, *HdF1a*, *HdF1b*, and *HdF2*, and one ferritin sequence was identified for *H. panicea*, *HpF1*. Using novel genomic data for *H. dujardini*, we found that the *HdF1b* gene is located 3538 base pairs away from the *HdF1a* gene in the same scaffold (distance between coding regions; see scaffolds accession numbers in Supplementary Table S1). In comparison with *HdF1a*, the *HdF1b* gene features only two nucleotide substitutions in the coding region, one of which does not change the encoded amino acid, and another one leads to an A35G replacement (corresponding to position 38 by conventional human ferritin *HuHF* numbering). The coding part of the *HdF2* gene sequence differs markedly from the *HdF1a/b* genes and is shorter by 6 nucleotides (Supplementary Table S1).

The genomic features of three *H. dujardini* ferritin genes were compared with those of marine (*Amphimedon queenslandica*, *Sycon ciliatum*, and *Oscarella pearsei*) and freshwater (*Ephydatia muelleri* and *Lubomirskia baikalensis*) sponges, which have published genomes (see sequences identifiers in Supplementary Table S1). At least two ferritin genes were found in each sponge species. All ferritin genes of *H. dujardini* and *S. ciliatum* are intronless, while genes of other species have two or three exons (Supplementary Table S1).

**Figure 1:**
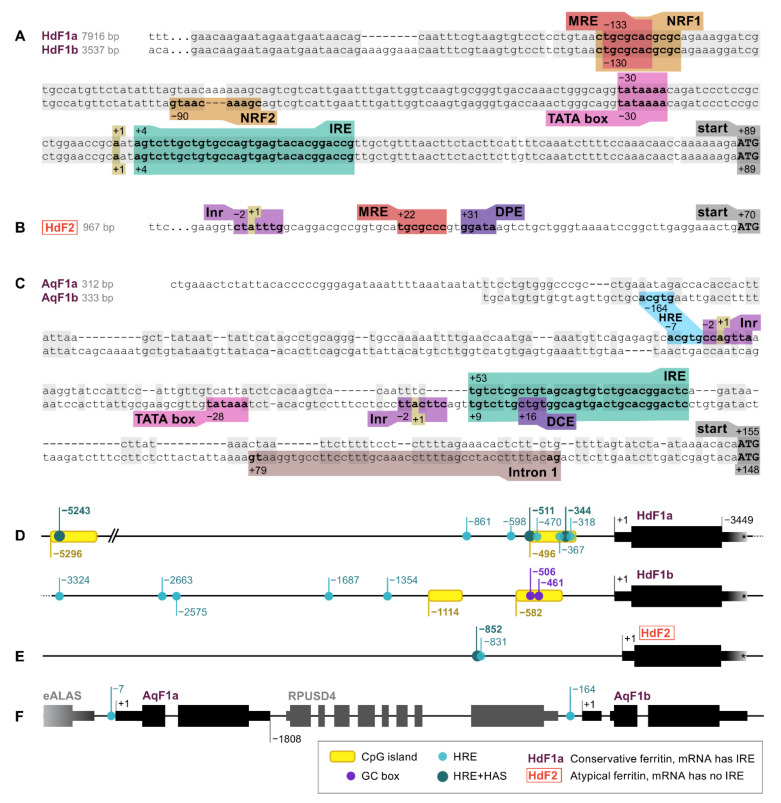
RNA and DNA regulatory elements in the ferritin genes of *H. dujardini* (*HdF1a, HdF1b*, and *HdF2*) and *A. queenslandica* (*AqF1a* and *AqF1b*). (**A**–**C**) 5′ sequences of sponge ferritin genes. (**D**–**F**) Arrangement of sponge ferritin gene copies, CpG islands, Hypoxia response elements (HRE) and Hypoxia ancillary sequences (HAS). 3′UTR lengths of *H. dujardini* ferritins are only shown schematically since unlike the 5′UTRs they have not been experimentally confirmed; the assembled transcripts are longer than depicted in the figure (Supplementary Table S1). MRE, metal regulatory element; IRE, iron-responsive element; Inr, initiator of transcription; DPE, downstream promotor element; DCE, downstream core element. See Supplementary Table S2 for motif sequences and other details.

Ferritin genes of marine sponges *H. dujardini*, *A. queenslandica*, and freshwater sponge *E. muelleri* were screened for RNA and DNA regulatory sequences, such as promoter elements and motifs involved in the regulation of ferritin expression. The identified elements are shown in Figure 1 and described in Supplementary Table S2. The 5′ upstream regions of *HdF1a/b* genes are highly similar up to 200 base pairs from the transcription start site (TSS) and, hence, contain a set of identified regulatory elements, whereas the pair of *A. queenslandica AqF1a/b* genes, which also originated from a recent duplication, have more diverse 5′ sequences (Figure 1A,C). The expression of *HdF1a/b* genes is controlled by a TATA-containing promoter located at position −30 upstream to the TSS. There is no TATA box in *HdF2*, instead, the CTATTT transcription initiation site (Inr) and downstream promoter element (DPE) were found at positions −2 and +31, respectively (Figure 1B). The promoter of *AqF1a/b* has a classical structure (Inr in *AqF1a*, Inr and TATA in *AqF1b*), and *AqF1b* features the downstream core element (DCE). EmF1 and EmF2 both have Inr. The *H. dujardini* 5′ upstream regions have CpG islands of length 204 and 206 bp (*HdF1a*), 206 and 269 bp (*HdF1b*), while short upstream regions of *AqF1a/b* and long upstream regions of EmF1 and EmF2 lack them. (Figure 1D,F). Two Sp1 transcription factor binding sites (GC box) were found in *HdF1b* at −506 and −461 inside a CpG island. There is a distal nuclear factor-κB (NFkB) motif located at position −5282 inside the CpG island of HdF1a. Metal-responsive elements (MRE) were found at various distances of the transcription start site, including proximal positions −133, −130, and +22 in *HdF1a, HdF1b*, and *HdF2*, respectively, while *A. queenslandica* ferritins lack them and *E muelleri’s* ferritins possess them only at distal positions (−6…−2 Kbp). Proximal MRE motifs found in *HdF1a/b* were contained within a larger motif of nuclear factor erythroid 2 (NFE2) -related factor 1 (NRF1), which is also present in EmF1 at −34. Interestingly, an NRF2 motif is identified in *HdF1b*, whereas in *HdF1a* it is disrupted by insertion of 3 bp. Hypoxia response element (HRE) motifs were found in all analyzed upstream regions, while more robust combinations of HRE and Hypoxia ancillary sequence (HAS), usually located by 7–15 bp downstream of HRE) were identified only in *HdF1a* (i.a. two are inside CpG island), *HdF2* and *EmF2* (Figure 1D–F, Supplementary Table S2). Weak CNC–sMaf binding element (CsMBE) motifs (also known as ARE and EpRE) were found at proximal positions in *HdF1b, EmF2* and distal positions in *HdF1a* and *EmF2*. Other putative motifs including Myb box, ABRE, NICE and Sph1 box were identified (Supplementary Table S2). Iron-responsive elements (IREs), mRNA hairpin motifs which are bound by the iron-regulatory protein IRP1, were found in the 5′ untranslated regions of *HdF1a/b, AqF1a/b*, and *EmF1*, as well as in *H. panicea HpF1* mRNA (Figure 1A,C, Supplementary Table S1).

The obtained transcriptome of *H. panicea* was supplemented with the data from another studies (NCBI project PRJNA394213, sample SAMN07484311; ENA project PRJEB43257). The only form of ferritin, designated earlier as *HpF1* (NCBI ID: QIZ30882.1), was found in all paired-end transcriptomic libraries. No other ferritin forms were found in the libraries except for contaminants. *H. panicea* ferritin transcripts form a large cluster of slightly different sequences (Supplementary Figure S1). To estimate the degree of ferritin polymorphism, we selected a single reference transcript and analyzed “transcriptomic SNP” frequencies in its coding part, mapping the reads from each library. It turned out that transcripts from different libraries had 30–40 polymorphic sites with frequencies more than 5% in the coding region of 513 nucleotides, with most of the SNP sites being consistent between all libraries (Supplementary Table S3). Thus, *H. panicea* ferritins are highly polymorphic having non-allelic differences.

### 2.2. Ferritin Proteins

The *H. dujardini* ferritins HdF1a and HdF1b each have 169 amino acids and differ slightly in the predicted molecular weights (MW): 19367.59 Da and 19353.56 Da, respectively. The isoelectric point pI for both is 4.91. The sequence of HdF2 protein differs significantly from the HdF1a/b, showing only 49% identity (69% similarity) with them and is shorter by two amino acids. Its predicted parameters are MW of 19425.84 Da and pI 5.87. The *H. panicea* ferritin HpF1 is closer to HdF1a/b than to HdF2: 60% identity (76% similarity) for HdF1a/b and 39% identity (60% similarity) for HdF2. HpF1 has 170 amino acids and a predicted MW of 19561.03 Da and pI 4.95.

**Figure 2:**
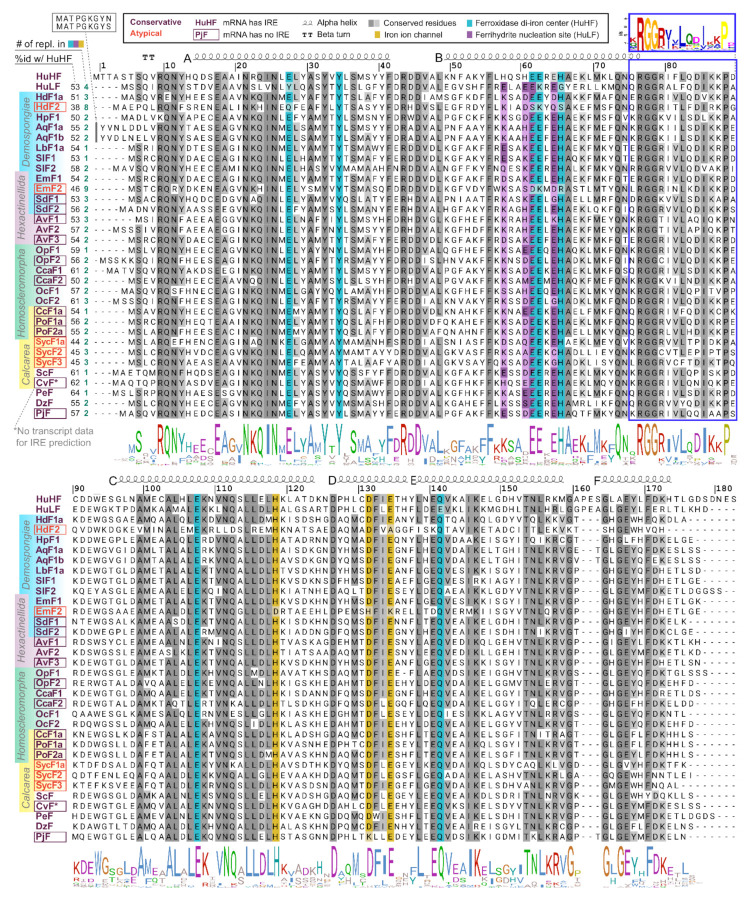
Domains and secondary structure of sponge ferritins. Alignment of ferritin amino acid sequences of *H. sapiens*, sponges of four classes, and five invertebrate species whose crystallographic data was recently obtained (the accession numbers are in Supplementary Table S1, for some species, only one representative gene copy product is shown). Amino acids numbering corresponds to conventional human HuHF numbering starting after the initial methionine. Highlighted are residues of three ferritin domains: iron ion channel (yellow), ferroxidase di-iron center (cyan), and ferrihydrite nucleation site (purple). To the left of the alignment are shown identity percentage level with human HuHF and the number of replacements in three domains. HuHF secondary structure is shown schematically above the alignment (PDB ID: 3AJO). A motif for the non-classical endosome secretion pathway is framed with blue.

To compare functional domains of sponge ferritins, we constructed multiple amino acid sequence alignment for a set of sponge, human, and several marine invertebrate ferritins, which 3D structures have been recently published (Figure 2, for identifiers see Supplementary Table S1). *H. dujardini* and *H. panicea* ferritins show very high sequence similarity with other sponges and invertebrates and contain known conserved functional domains of H chain ferritins, namely the iron ion channel and ferroxidase di-iron center. The residues corresponding to ferrihydrite nucleation center, a domain usually attributed to L chain ferritins, do not follow the domain consensus in the majority of analyzed sponges. *H. djuardini* ferritin HdF2 is the least conserved and has substitutions in the key residues of functional domains: E27Q, E61S, E62K, H65S (ferroxidase di-iron center/ion binding site), and E134A (iron ion channel); all numbers follow conventional human HuHF numbering starting after initial methionine (Figure 2). The N-glycosylation (GlcNAc) site Nx[ST] (residues 111–113) was found in AqF1a, AvF1, AvF2, OpF1, OcF1, OcF2, CcF1a, PoF1a, PoF2a, and SycF3. The conserved protein kinase C phosphorylation site [ST]x[RK] (residues 144–146) present in freshwater sponge ferritins EmF1, SlF1a/b, LbF1a/b, and among other sponges: AvF3, OpF1, OcF1. All sponge ferritins lack the signal peptide for classical ER-Golgi secretion but have a non-classical endosome secretion pathway motif of 15 amino acids starting from xRGG in the BC interhelical region (framed with blue in Figure 2, for motif q-values see Supplementary Table S1). A short turn between helices C and D (D126) is highly conserved in most studied invertebrates (Figure 2).

**Figure 3:**
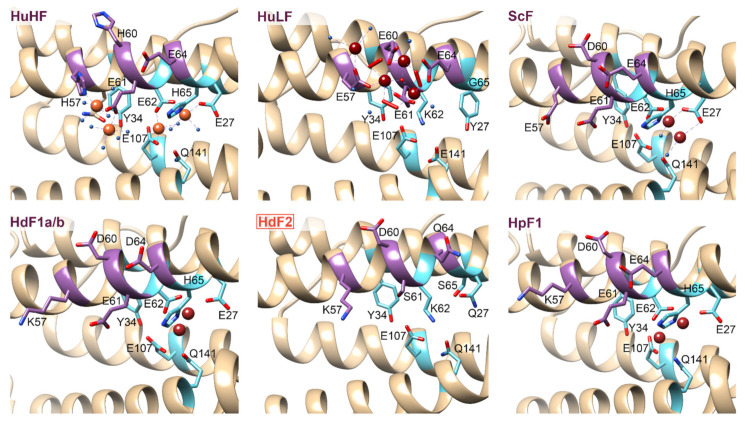
3D structures of the regions corresponding to ferroxidase di-iron center (cyan) and nucleation site (purple) of *H. sapiens* ferritins HuHF and HuLF (PDB IDs: 4OYN, 5LG8), bivalve *Sinonovacula constricta* ScF (PDB ID: 6LP5), and sponges *H. dujardini* and *H. panicea* ferritins. Sponge ferritins’ structures are modeled with SWISS-MODEL server [41] using ScF as a template. Molecular graphics was prepared using UCSF Chimera software [42]. Orange and brown spheres represent Fe^3+^ and Fe^2+^ ions, respectively. Amino acids numbers correspond to conventional human HuHF numbering starting after the initial methionine.

The 3D structures of *H. dujardini*, *H. panicea, A. queenslandica*, and *E. muelleri* ferritin domains complexed with iron ions were reconstructed by homology modeling using a structure of *Sinonovacula constricta* ferritin ScF (PDB ID: 6LP5) as a template, since it was the closest homologue with the known structure complexed with iron ions, having the sequence identity of 63.2%, 46.3%, and 55.1% with HdF1a/b, HdF2, and HpF1, respectively (Figure 3). Other possible templates included ferritins of marine worms *Phascolosoma esculenta* and *Dendrorhynchus zhejiangensis* (PDB IDs: 6LPD, 7EMK). The predicted models have good estimated quality (QMEAN scoring function values are 0.14, −1.77, and 0.08 for HdF1a/b, HdF2, and HpF1, respectively). In the HdF1a/b, HpF1, AqF1a/b, and EmF1 ferritins, the iron atoms in the structures are predicted to be connected only at the ferroxidase center. In HdF2 and EmF2, the iron-binding activity is not predicted at all (Supplementary Table S1). Transient heme binding was predicted for HdF1a/b, HdF2, HpF1, EmF1, and EmF2 ferritins for histidine residue at the position corresponding to L165 in HuHF (Supplementary Table S1).

### 2.3. Phylogenetic Analysis of Sponge Ferritins

In order to reveal the presence of atypical non-conservative ferritins similar to HdF2 in other invertebrate species, we compiled a large dataset from annotated and unannotated transcriptomic databases, collecting 533 ferritin sequences belonging to 264 invertebrate species from more than 30 classes (see Section 4, Supplementary Table S1). Surprisingly, the hits found in all available Ctenophore databases represent only distant homologues of ferritin (Supplementary Table S5), therefore, no ctenophore sequences were included in the dataset. Among analyzed ferritins, 70% of insects’ and only 25% of other invertebrates’ sequences have signal peptides (SP), while all analyzed sponge ferritins lack them.

**Figure 4:**
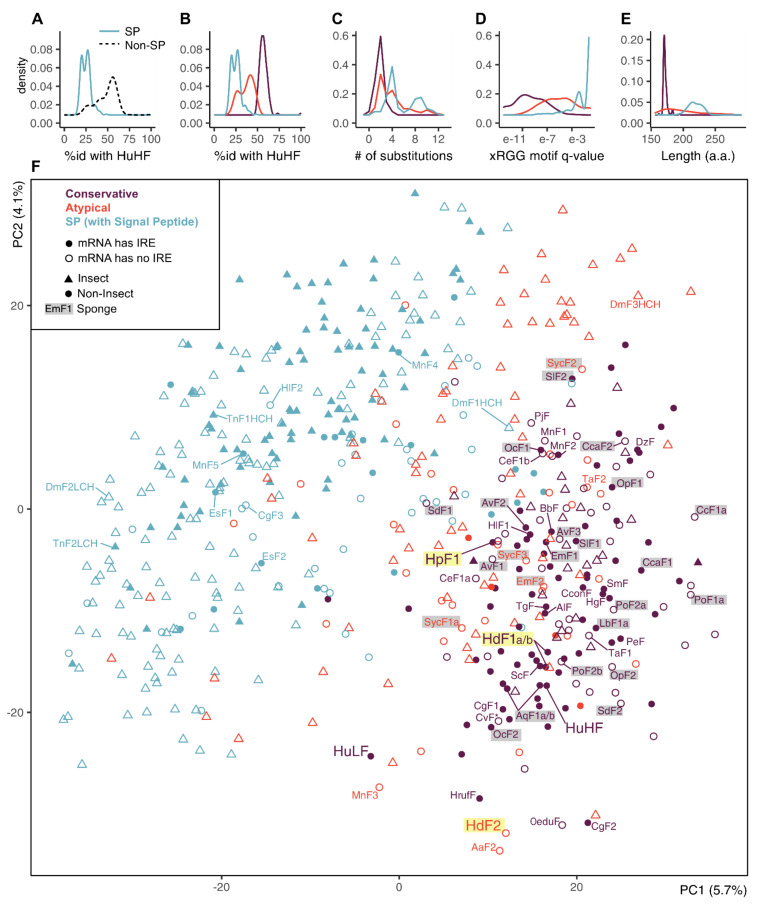
Principal component analysis (PCA) plot on a set of animal ferritins built using 474 sequence features selected by unsupervised algorithms. (**A**,**B**) Distribution of identity percentages with HuHF in ferritins of two classes of SP and SP-less ferritins and after dividing SP-less ferritins into conservative and atypical classes. (**C**–**E**) Distributions of the number of replacements in the three functional domains, the xRGG motif q-value, and protein sequence lengths in the ferritins of three classes. (**F**) PCA plot of 533 analyzed invertebrate and human ferritin sequences (Supplementary Table S1) built by selected feature subset (474 out of 6567 sequence features combined from top-100 features selected by five unsupervised feature selection algorithms, see Section 4, Supplementary Table S4) and showing prior class labels. Sponge ferritins are highlighted with grey, *H. dujardini* and *H. panicea* ferritins are highlighted with yellow. The large version of this plot with all the labels is shown in Supplementary Figure S2.

The SP-ferritins have low identity to human HuHF (Figure 4A). The SP-less ferritins generally have higher identity to HuHF, but the distribution of identity levels is skewed, suggesting non-uniform conservation and the presence of infraclasses. We defined two such infraclasses of SP-less ferritins, conservative and atypical (the atypical is defined as having less than 49% sequence identity with HuHF or more than five amino acid replacements in the three functional domains) (Figure 4B,C). The atypical ferritins occupy an intermediate position between the conserved non-SP ferritins and SP-ferritins in terms of the identity levels with HuHF, the xRGG motif enrichment, and the sequence length (Figure 4B–E). The proportion of conservative and atypical classes in analyzed ferritins differs for insects (8% and 23%) and other invertebrates (57% and 18%). In sponges, atypical ferritins are represented by HdF2 (37.7% identity with HuHF and 8 replacements), *E. muelleri* EmF2 (46.4% id; 9 repl.), and all *S. ciliatum* ferritins: SycF1a/b, SycF2, SycF3 (44.3–45.4% id; 2–3 repl.). Nearly all atypical ferritins lack iron-responsive element (IRE), the few exceptions can be considered as marginal cases of conservative ferritins (47–49% id; 2–3 repl.). The overall level of IRE presence is 47% for non-insect and 29% for insect ferritins. IREs are mostly found in the ferritin classes with the largest representation in the group: 63% of conservative ferritins outside of insects, and 41% of insect SP-ferritins (Supplementary Table S1, sheet Statistics).

To identify possible common characteristics shared by atypical invertebrate ferritins, we performed dimensionality reduction on analyzed sequences’ features. First, a comprehensive protein sequence feature set was obtained that includes simple, grouped, and pseudo amino acid composition, composition/distribution/transition, Geary, Moran, and normalized Moreau-Broto autocorrelation features for each sequence. Next, this set was reduced from 6567 to 474 features (7.2%) combining top-100 features selected by five unsupervised feature selection algorithms (see Section 4, Supplementary Table S4). We did not infer new clusters but transferred our class labels to a PCA projection built for the selected feature subset. The PCA demonstrates a relatively clear differentiation between the SP-less and SP-ferritins, but the differentiation between conservative and atypical ferritins is indistinct (Figure 4F, Supplementary Figure S2). The sponge ferritins, atypical HdF2, SycF1a/b, SycF2, conservative HpF1, SdF1, SdF2, SlF2 lie on the periphery of the conservative ferritin cluster, as well as the human HuLF. At the same time, atypical EmF2 and SycF3 are closer to the center of the cluster, together with the conservative HdF1a/b, AqF1a/b, and human HuHF. Bivalve ferritin ScF, which was used as a template for 3D modeling, is located very close to HdF1a/b.

**Figure 5:**
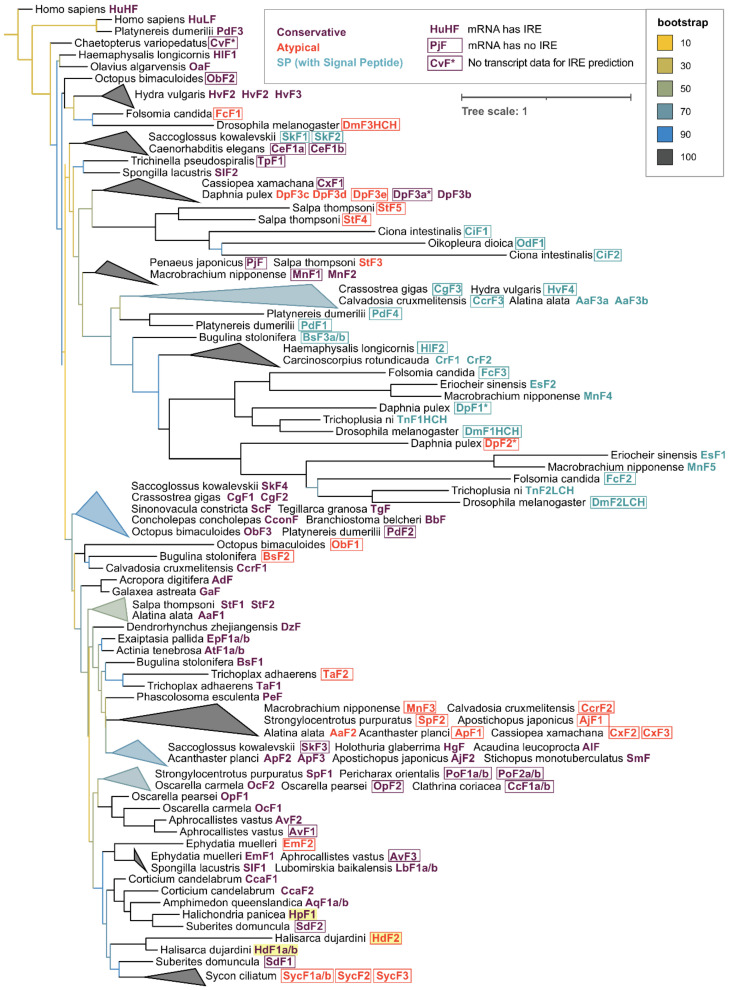
Phylogenetic tree of invertebrate and human ferritins. 131 representative ferritins from 56 species of 32 invertebrate classes (Supplementary Table S1) along with human ferritins were aligned using clustalo algorithm [43], the alignment was processed with Gappyout algorithm using trimal v. 1.2 software [44] to remove variable ends and regions of signal peptide (132 core residues left). The tree was constructed using the Maximum likelihood approach with 1000 fast bootstrap resamplings with IQ-Tree v. 1.6.12 [45] and visualized using iTOL server [46], with ferritin sequences of *Homo sapiens* used as an outgroup. Triangles represent collapsed clades with upper and lower points showing the range of branch lengths inside a clade. The full version of this tree without collapsed branches is shown in Supplementary Figure S3.

For phylogenetic analysis, the initial collection of 533 ferritins was reduced to 131 sequences while maintaining the representatives of all main classes since the majority of annotated ferritin sequences belonged to a single class Insecta (58% of the whole dataset). The phylogenetic tree built with the selected subset of 131 invertebrate ferritins with human ferritins used as an outgroup shows that the sequences tend to group not only by the respective taxonomy but also frequently with regard to the defined ferritin classes (Figure 5, Supplementary Figure S3). Interesting examples of grouping by ferritin class are mollusks’ (including bivalve ScF) and Cnidarian conservative ferritins, atypical ferritins from different phyla of marine invertebrates and a large cluster of SP-ferritins. Sponge ferritins form a distinct cluster in the tree, with the freshwater species clustering together. Among the most evolutionary distant sponge ferritins are atypical HdF2, EmF2, ferritins of Calcarea sponge *S. ciliatum*; conservative HpF1, SdF2, and Hexactinellida sponge *A. vastus* AvF1, AvF2.

### 2.4. Ferritin Complexes

**Figure 6:**
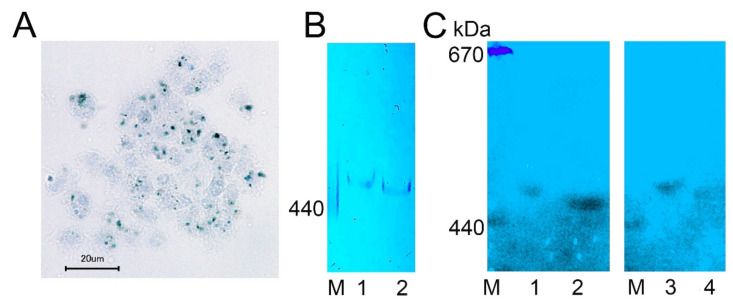
Ferritin complexes in sponges *H. dujardini* and *H. panicea*. (**A**) The ferric complexes detected in *H. dujardini* cells by Prussian blue staining. (**B**,**C**) Sponge cell extracts fractionated by native electrophoresis in polyacrylamide gel: staining with Prussian blue (**B**) and the UV 360 nm absorbance (**C**). Specimens of *H. panicea* collected in Autumn (1) and Summer (3). Specimens of *H. dujardini* collected in Autumn (2) and Summer (4). M, horse ferritin (440 kDa) and thyroglobulin (670 kDa).

We visualized complexes containing ferric ions in *H. dujardini* cells by using specific Prussian blue staining (Figure 6A, Supplementary Figure S4N). The ferric complexes were observed in cells as optically dense areas of different sizes. Fractionation of the cell extracts by a native electrophoresis in polyacrylamide gels followed by staining with Prussian blue revealed ferritin complexes in both sponges, *H. dujardini* (lacking spicules) and *H. panicea* (having spicules) (Figure 6B, Supplementary Figure S9). The *H. dujardini* ferritins migrated a little faster than that of *H. panicea*, but both complexes migrated slightly slower than the horse ferritin marker (440 kDa). As expected, the ferritin complexes in gels showed absorption of UV light near 360 nm wavelength (Figure 6C, Supplementary Figure S9).

**Figure 7:**
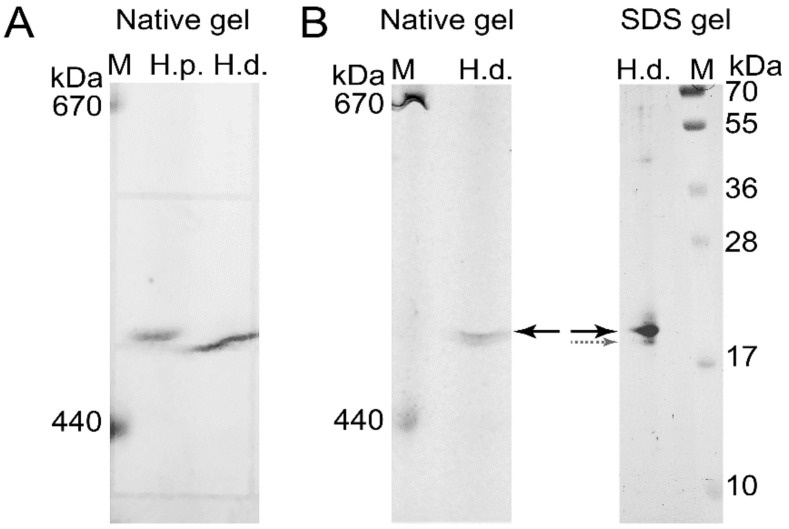
Isolation of *H. dujardini* and *H. panicea* ferritins by electrophoresis in a native polyacrylamide gel for mass spectrometry. (**A**) The native polyacrylamide gel stained with Coomassie blue without fixation. (**B**) The band corresponding to *H. dujardini* ferritin complex (marked by solid black arrow) was taken from the native gel, denatured, subjected to SDS-12% PAGE electrophoresis, and then stained with Coomassie blue. The major and minor bands indicated by solid black and dotted grey arrows were taken for MALDI/TOF mass spectrometry. M, horse ferritin (440 kDa) and thyroglobulin (670 kDa). H.p., *H. panicea;* H.d., *H. dujardini*.

To confirm the presence of *H. dujardini* and *H. panicea* ferritins in the high molecular mass complexes detected in native gels, the corresponding bands were cut from the native gel stained with Coomassie blue without fixation (Figure 7A) and then analyzed by MALDI/TOF mass spectrometry. The HpF1, HdF1a/b, and HdF2 ferritins were identified in these bands (Supplementary Table S10, Supplementary Figure S8). In addition, the ferritin band of *H. dujardini* (specimens collected in Summer) was cut from the native gel (Figure 7B), denatured in SDS-containing buffer and analyzed by the SDS-12% PAGE electrophoresis followed by Coomassie staining. One major band of approximately 20 kDa was revealed (Figure 7B) that well corresponds to a predicted molecular mass of HdF1a/b equal to 19.4 kDa. The MALDI/TOF mass spectrometry confirmed the presence of HdF1a/b in this band. The minor band of approximately 19 kDa that resolved by this analysis (Figure 7B) corresponds to *H. dujardini* neuroglobin (QEH04777.1, Supplementary Table S10).

### 2.5. Subcellular Localization of Ferritins

**Figure 8:**
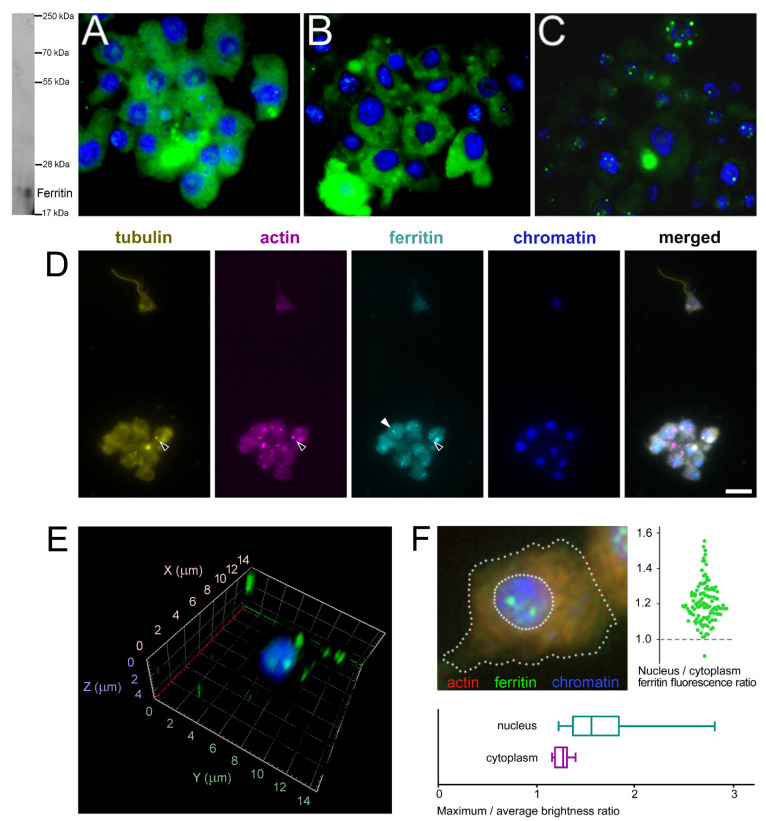
Subcellular localization of ferritin in *H. dujardini* cells. (**A**–**C**) Ferritin immune staining. Ferritin (green), Chromatin (blue). Western blot on the right shows the antibody specificity. Staining of cells collected at the end of sponge growth in Autumn (**A**), and at the beginning of sponge growth in Summer (**B**,**C**). The focal planes of maximum intensity presented. (**D**) Sponge cells, quadruple stained for tubulin, actin, ferritin and chromatin. Black arrows, cytoplasmic ferritin colocalized with both tubulin and actin; white arrow, cytoplasmic granule of ferritin without visible signs of colocalization with cytoskeletal proteins. Scale bar 10 μm. Confocal microscopy of intranuclear ferritin in the isolated nuclei, 3D reconstruction. (**E**) Ferritin (green), Chromatin (blue). Intranuclear ferritin granules are co-purified together with nuclei. (**F**) Sponge cell, triple stained for actin, chromatin and ferritin (dashed lines denote boundaries of the cell and the nucleus). Swarmplot shows the ratio of average fluorescence levels between ferritin localized in the nucleus and in the cytoplasm of each cell, *N* = 114. Boxplot compares the ratio of the maximum to the average fluorescence level of ferritin localized in the nucleus and in the cytoplasm of each cell, *N* = 114.

**Figure 9:**
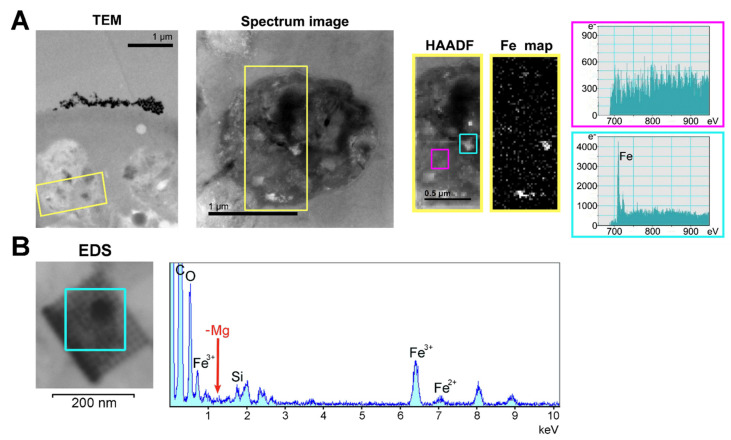
Identification of the ferric ion-containing granules in *H. dujardini* cells. (**A**) Transmission electron microscopy of the fragment of sponge cell with inclusions. HAADF and Fe map panels represent the area outlined in yellow on the panel Spectrum image which in turn is a section of the TEM panel. The spectra outlined in cyan and magenta were taken from the corresponding outlined areas in the HAADF panel. Scale 1 μm. (**B**) EDS-analysis of the region where the presence of iron atoms was confirmed by the HAADF method. TEM image of this area in higher resolution is shown in Figure S7. Spectrum data for the plot can be seen in Supplementary Table S9.

The subcellular localization of *H. dujardini* ferritin was studied using an immune fluorescence assay. The cytoskeletal proteins, actin, and tubulin, were visualized by specific antibodies. Ferritin shows predominantly diffuse distribution in the nucleus and cytoplasm of sponge cells (Figure 8A,B, Supplementary Figure S4A–M) with a number of speckles producing bright fluorescent spots under immunostaining (Figure 8C). The ratio of ferritin fluorescence in the nucleus and cytoplasm was 1.21 ± 0.11 (0.01) {mean ± Sd (Se)} for 114 cells (Figure 8F, swarmplot). This value indicates roughly similar spreading of ferritin between nucleus and cytoplasm in sponge cells where the nuclear area does not differ significantly in size from the cytoplasmic area in most cells. However, ferritin is distributed more heterogeneously inside the nucleus than in cytoplasm as illustrated by the higher ratio of maximum brightness to average brightness in the nucleus than in cytoplasm (nucleus: 1.80 ± 0.64 (Sd) vs. cytoplasm: 1.30 ± 0.09 (Sd) (Figure 8F, boxplot)). Moreover, the standard deviation values clearly indicate the higher variation in the nuclear staining than in cytoplasmic staining. The colocalization of ferritin with actin and tubulin was observed in cells with or without flagella as well as in dissociated and reaggregated cells (Figure 8D, Supplementary Figure S4A–H). The bright spots of ferritin complexes varied in numbers from 1–2 to 8 or more and were located largely in the nucleus (Figure 8E,F). The nuclear speckles of ferritin were more pronounced in cells at the beginning of sponge growth (specimens collected in August) (Figure 8C). The presence of the storage iron form, oxidized ferric ions (Fe^3+^), in the electron-dense subcellular structures presumably associated with ferritins in sponge cells was directly confirmed by the transmission electron microscopy accompanied by spectral analysis (Figure 9). The Fe^3+^ iron signal was clearly recognized in the spectrum of the electron-dense area lacking the magnesium atoms. This result confirmed the accumulation of ferric ions in specific subcellular structures in sponge cells revealed by staining with Prussian blue (Figure 6A).

### 2.6. Expression of Ferritins and the Ferritin Associated Factors during Different Periods of the Annual Cycle

**Figure 10:**
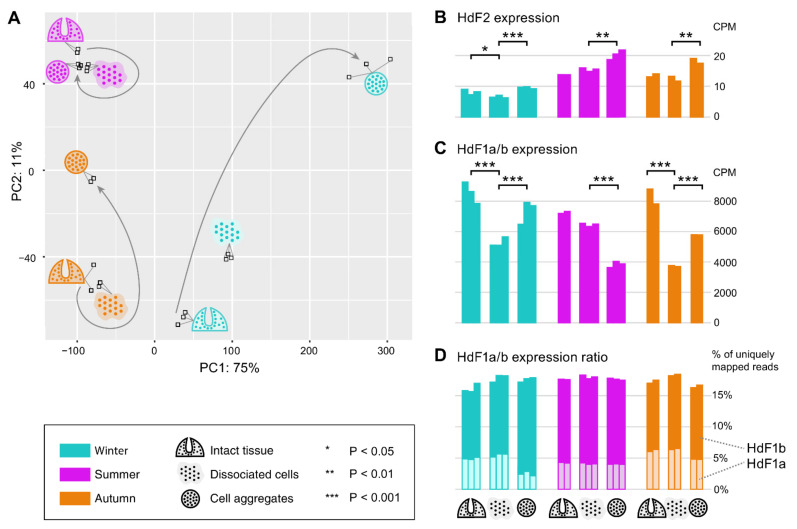
Expression of ferritin genes during the dissociation/reaggregation processes in sponge *H. dujardini* during the different periods of the annual cycle. (**A**) Principal component analysis (PCA) plot of RNA-Seq samples colored by the life cycle stages (Winter, cyan; Summer, purple; Autumn, orange) and marked by the pictograms of the stages of the dissociation/reaggregation experiment. (**B**,**C**) Ferritin *HdF2* and *HdF1a/b* transcript expression in CPM (calculated by edgeR after normalization), each bar represents one replicate. Statistical significance is shown for BH-adjusted *p*-values of the t-test carried out for replicates of each period. Since the *HdF1a* and *HdF1b* copies are nearly identical, their expression is shown in total (reads were mapped to one transcript). (**D**) Stacked barplot showing the percentage of the reads mapped uniquely to *HdF1a* (light boxes) or *HdF1b* (dark boxes) gene copy, out of the total number of reads mapped to these copies, that could be used as an estimate for the transcriptional ratio between two copies.

In order to reveal possible involvement of ferritin and other iron metabolic proteins in the morphogenetic processes in sponges, we carried out transcriptomic sequencing for samples of *H. dujardini* collected at different periods of their life cycle: Winter (the beginning of spermatogenesis and oogenesis), Summer (the beginning of sponge body tissue growth), and Autumn (the end of sponge body tissue growth), and at different reaggregation states (intact sponge body tissues, dissociated cells, and reaggregated cells). Our previous data for Autumn samples (NCBI PRJNA594150, samples SAMN13506244‒SAMN13506251) were combined with new data for the Summer and Winter samples that were deposited to the National Center for Biotechnology Information (NCBI PRJNA594150, samples SAMN20337311‒SAMN20337327). The average number of single-end 50 bp reads per sample was 28 M with the total number of 476 M reads for 17 new samples of *H. dujardini*. On average, 99.8% of reads passed the quality filter, of which 95.1% were successfully aligned to the transcriptome assembly constructed earlier (NCBI TSA: GIFI00000000.1) (Supplementary Table S6). Although the studied growth periods are represented predominantly by somatic, non-reproductive cells in the sponge body, the biological coefficient of expression variation showed the value of 0.73, thus confirming the transcriptional diversity of the studied samples. The PCA plot reveals clusters of samples associated with the aggregation states of sponge cells from each of the life cycle stages (Figure 10A). Additionally, the samples associated with the beginning of sponge growth (Summer) cluster more tightly than the other life cycle stages. The reaggregated cells from the Winter and Autumn periods tend to separate from the other aggregation states of the corresponding seasons.

Ferritins *HdF1a/b* are among the most expressed genes in *H. dujardini*, along with such genes as actin and tubulin (Supplementary Table S7). In the cells of intact sponges, their expression was similar in all studied life periods (Figure 10C). The *HdF1a/b* expression markedly decreases in the dissociated cells of sponges collected in Winter and Autumn, and partially recovers during reaggregation. In contrast, the *HdF1a/b* expression does not change significantly after dissociation in samples collected in the Summer season but markedly decreases under reaggregation (Figure 10C). Interestingly, the relative proportion of a/b copies was similar in all studied samples, except for the aggregated cells of the Winter samples, where the proportion of *HdF1a* was markedly decreased (Figure 10D). The *HdF2* expression in the Winter samples, associated with the onset of spermatogenesis and oogenesis, was markedly lower than in the Summer and Autumn samples. The aggregation of cells was accompanied by an increase in *HdF2* expression in all samples (Figure 10B).

The expression levels of other factors involved in iron metabolism, heme biosynthesis and transport, response to hypoxia and ferroptosis were lower than that of ferritins *HdF1a/b* (Figure S5, Supplementary Table S7). Actual expression levels are dependent on the seasons when the sponge samples were collected and affected by the dissociation/reaggregation processes. Most factors involved in the iron and heme metabolism (SQSTM1, NAALAD2-like 1, IPR1/ACO1, NFKB1, GAPDH, ABCG2, HRG1-like, globin NGB) have a higher expression level in body tissues in the Summer period than in Winter or Autumn (Supplementary Figure S5). The same was true for the hypoxia factors HIFa/SIM-like 2/3, anti-apoptotic protein BCL2, and ferroptosis factors, prominin 1, TGFBR1-like 2 and glutathione peroxidase GPX-like 2. Expression of BCL2 increases under reaggregation of cells, meanwhile SQSTM1 expression increases during reaggregation only in Winter and Autumn samples but remains unchanged in Summer samples. The metal-regulatory transcription factor 1 (MTF1) linked to the regulation of ferritins is expressed in sponge bodies at a low level at all seasons, but it is markedly induced during cell reaggregation in Winter samples. Reaggregation also increases expression of the heme transporter ABCB6 in all seasons and that of transporter ABCG2 in Winter. Among the heme biosynthesis enzymes (ALAS, ALAD, and FECH/hemH), expression of ALAS and ALAD decreases during reaggregation in all samples, but increases for FECH/hemH in Winter samples. Expression of neuroglobin NGB changes differently during dissociation/reaggregation processes in sponges collected in different seasons. Reaggregation is accompanied by an increase in NGB expression in the Winter and Autumn samples but not in the Summer samples. Another expression pattern was observed for Cathepsin D and Casp3: the basal expression level in sponge tissues in Winter and Autumn was lower than that in Summer, and it markedly decreases during the reaggregation process. The differential expression pattern of factors involved in iron metabolism at different periods of sponge life cycle briefly described above reveals intricate regulation of morphogenetic processes in sea sponges.

### 2.7. Ferritin Superfamily Members of the Microbial Community of H. dujardini

Bacterial representatives of the ferritin superfamily were identified in the transcriptomic data of *H. dujardini*, namely, rubrerythrins, non-heme ferritins, bacterioferritins, and DNA starvation/stationary phase protection proteins (Dps) (Supplementary Figure S10, Supplementary Table S8). The top hits obtained via blastp by querying against bacterial ferritin sequences of NCBI Protein database have good sequence coverage (68–100%) and identity values (44.6–100%) (Supplementary Table S8). All these microbial symbionts were Gram-negative bacteria that belong primarily to *Alphaproteobacteria* and *Gammaproteobacteria* in the phylum *Proteobacteria*. Some of them could be identified at the family level (*Rhodospirillaceae, Rhodobacteraceae, Rhizobiaceae, Xanthomonadaceae, Thiotrichaceae*) or at the genus and species levels: *Magnetospira* sp., *Magnetospirillum gryphiswaldense*, *Desulfovibrio desulfuricans*. *Labrenzia* sp., *Halovulum dunhuangense*, *Bosea* sp., *Pseudomonas asplenii*, *Albidovulum inexpectatum*, *Ruegeria pomeroyi*, *Brevundimonas bullata*, *Kiloniella litopenaeus*, *Nisaea denitrificans*, *Oceanibaculum nanhaiense*, *Chryseolinea flava*, *Notoacmeibacter marinus*, *Endozoicomonas* sp., *Vibrio cyclitrophicus*, *Magnetovibrio* sp. Interestingly, the expression of ferritin superfamily members of many bacterial phyla, except for Cyanobacteria, showed lower levels in the sponge bodies collected in Summer compared to sponges collected in Autumn (Supplementary Table S8).

## 3. Discussion

In this report, we characterized ferritins in two sea sponges, *H. dujardini* lacking spicules and *H. panicea* having spicules. Dissociation of the sponge bodies and cell reaggregation was used as a model of the morphogenetic processes in sponges. Three ferritin genes *HdF1a, HdF1b*, and *HdF2* were found in *H. dujardini*, and one gene *HpF1* in *H. panicea*. The level of similarity of *HdF1a* and *HdF1b* (only two substitutions in coding sequence) and their close colocalization on the same scaffold suggest the very recent duplication of the parental *HdF1a/b* gene. The *HdF2* gene markedly differs in sequence, but groups with the *HdF1a/b* in the phylogenetic trees, suggesting a more ancient duplication gave rise to the *HdF1* and *HdF2* genes. The highly polymorphic *H. panicea* ferritins have non-allelic differences (Supplementary Figure S1, Supplementary Table S3) and may indicate weakened genetic control over the *HpF1* gene. Each sponge species with a previously published genome possesses at least two ferritin genes, and at least one tandem duplication event was observed (Supplementary Table S1).

The ferritins of sponges form a well-supported cluster in the phylogenetic tree of invertebrate ferritins (except for SlF2) with the subclades of freshwater and Calcarea sponges (Figure 5). The *H. dujardini* ferritin genes *HdF1a* and *HdF1b* have an almost identical set of proximal regulatory elements, whereas *HdF2* regulatory and promotor elements are different (Supplementary Table S2), which likely explains their markedly contrasting expression levels (Figure 1A,B, Figure 10D). HRE motifs found in upstream regions of *HdF1a* and *HdF2* (i.a. within CpG islands) presumably make them sensitive to hypoxia [15]; in turn, we have previously shown that hypoxic response is important for sponge reaggregation process [24]. *HdF1a*, *HdF1b*, and *H. panicea*’s *HpF1*, like most Demospongiae and non-insect invertebrate ferritins, have mRNA hairpin motifs of iron-responsive elements (IREs) interacting with the iron-regulatory protein IRP1 (Supplementary Table S1). However, no IRE was predicted for minor *H. dujardini HdF2*, as well as for *Ephydatia muelleri EmF2*, ferritins of *Suberites domuncula* and all Calcarea ferritins. The lack of IRE correlates with evolutionary distance, placing such ferritins into longer branches and on the periphery of the conservative ferritins cluster (Figure 4 and Figure 5). The combined presence of IREs and MREs in the 5′UTRs of *HdF1a* and *HdF1b* suggests that their expression is under strict regulation by iron ions.

The 3D modeling predicts that the *H. dujardini* and *H. panicea* ferritins HdF1a, HdF1b, and HpF1 are capable of binding iron atoms only in the ferroxidase center (Figure 3, Supplementary Table S1). Atypical HdF2 and *E. muelleri*’s EmF2 have multiple amino acid replacements in this domain, and no iron-binding activity is predicted for them (Supplementary Table S1). HdF2 also has E134A substitution in the iron ion channel, which was shown to decrease the capacity of the protein to incorporate iron [47]. HdF2 presumably is a minor ferritin in *H. dujardini* that lost the iron-binding capacity in the evolution and is exploited for novel functions in sponges. The iron-binding activity in the ferrihydrite nucleation site is not predicted for all studied sponge ferritins. However, heme-binding is predicted for HdF1a/b, HdF2, HpF1, EmF1, and EmF2 ferritins. The inclusions of different sizes containing ferric ions were observed in *H. dujardini* cells after specific staining with Prussian blue (Figure 6A), and the presence of ferric ions in these inclusions was directly confirmed by the spectroscopy analysis (Figure 9). Moreover, the high-resolution TEM image of the Fe-containing region shows heterogeneity due to numerous structures similar to ferritin beads [48] (Supplementary Figure S7). According to the mobility in native polyacrylamide gels, the sponge ferritins form a stable complex of 24 subunits as ferritins in other animals (Figure 6B,C, Figure 7). Association of Fe^3+^ ions with these multisubunit forms of ferritins was confirmed by staining with Prussian blue (Figure 6B). The MALDI/TOF mass spectrometry confirmed the presence of the major ferritin forms HdF1a/b and the minor form HdF2 in the complexes isolated from native gels (Figure 7, Supplementary Table S10), making plausible the existence of native ferritin complexes comprising a mixture of the major and minor forms. Such inclusion of not iron-binding HdF2 into the complexes might be used for fine-tuning their iron-binding capacity. HdF2 may also facilitate the exchange of iron ions between the ferritin complexes and cellular media.

We suggest that ferritins of *H. dujardini* and *H. panicea* are secreted from cells by a non-classical endosome secretion pathway. Ferritins of *H. dujardini* and *H. panicea* lack signal peptides (SP), as well as the N-glycosylation sites and the protein kinase C phosphorylation sites, involved in the ferritins secretion in Polychaeta and Malacostraca [49]. Instead, sponge ferritins contain xRGG-motif that is highly enriched in all SP-less ferritins presumably secreted by the non-classical secretion pathway [50]. The amino acids in that interhelical motif are mostly exposed to the solvent and likely participate in protein–protein interactions. In this respect, the sponge ferritins could be similar to ChF ferritin of the marine polychaete worm *Chaetopterus* sp. that also lacks SP and still is mostly secreted [21].

The tissues cells of *H. dujardini* highly express ferritin genes *HdF1a/b* in all studied periods of the annual cycle and keep both ferritins HdF1a and HdF1b as constituents of the cellular proteome (Figure 7 and Figure 10). Expression of *HdF1a/b* changes specifically during the dissociation and reaggregation processes depending on the sponge life cycle period (Figure 10), indicating involvement of ferritins in the metabolic rewiring of cells in the course of morphogenesis. The beginning of the sponge growth period (Summer) is characterized by high expression of factors involved in the heme and iron metabolism, NAALAD2, ABCG2, GAPDH, and NGB, as well as transcription factors HIFa/SIM-like and NFkB (Supplementary Figure S5). The sponge cells are exposed to oxygen and metabolite gradients during the body dissociation and cells reaggregation. During these transformations resembling transflammation [51], ferritins presumably regulate the redox processes in cells by maintaining the balance of iron ions between intracellular metal-binding complexes (ferritins, heme, Fe-S clusters) and the extracellular pool. Secretion of iron ions should prevent ferroptosis and cell death. Expression of the autophagy and apoptosis regulator SQSTM1 and ferroptosis factors, prominin 1, TGFBR1-like 2, and GPX-like 2, may support the viability of cells under differentiation and sponge remodeling [52,53].

Some sponge ferritin complexes may be associated with endosymbionts. It has been shown that bacteria are present inside *H. dujardini* cells [54], whereas *H. panicea* exemplifies low microbial abundance sponges and is dominated by an extracellular alphaproteobacterial symbiont [55]. The microbial symbionts may affect the iron metabolism in sponges depending on annual seasons. Iron-chelating Cyanobacteria are more abundant in summer when they bloom in surface waters [56]. Iron is essential for the successful colonization of biotopes by Gram-negative bacteria [57], which are highly enriched in the sponge microbiome (Supplementary Figure S10, Supplementary Table S8). These bacteria release iron from heme, which can then be trafficked to the intracellular space. Gram-negative bacteria regulate the innate immunity of invertebrates and vertebrates by mechanisms involving ferritins [58,59]. Interestingly, ferritin MnF5 of *M. nipponense*, which like HdF2 lacks iron-binding capacity was up-regulated following the bacterial challenge [60]. A similar mechanism may be responsible for the induction of *HdF2* expression in the course of cells reaggregation (Figure 10). Ctenophora develop immune tolerance to the microbial community [61,62] and retain NGB, ADGB, and other factors involved in the iron metabolism, which show approximately 40% to 50% identity to the sponges’ homologs (Table S5), but appear to lack genes for ferritins or genes for NFkB and BCL2 [63]. Many heme-containing factors including ancient neuroglobin NGB and ADGB may functionally and physically interact with ferritins. The gel electrophoresis and mass spectrometry confirmed the association of the heme-containing NGB with sponge *H. dujardini* ferritin complex (Figure 7B, Supplementary Table S1). The immunofluorescence assay of sponge cells confirmed partial colocalization of ferritin with cytoskeletal proteins, actin and tubulin (Figure 8), which has been found in neurons, macrophages, liver and kidney cells of mammals [64,65], as well as in Honeybee trophocytes [20]. The ferritin complexes are also found in the nucleus of sponge cells (Figure 8C–F, Supplementary Figure S4), where they may submit iron ions to the Fe-S clusters in enzymes participating in replication and repair of nuclear DNA. Our research confirmed that the sea sponges serve as a promising model for investigation of ferritins and iron metabolism in evolutionary distant animals.

## 4. Materials and Methods

### 4.1. Specimen Collection

Specimens of the cold-water sea sponges *Halisarca dujardini* and *Halichondria panicea* were collected in the sublittoral at a low tide near the N.A. Pertsov White Sea Biological Station of Lomonosov Moscow State University (66°340 N 33°080 E). The water temperature at the time of collection was and 0 + 2 °C (January), +4 + 5 °C (November) and +12 + 15 °C (August). The sampling was done in a way that sponges remained attached to the substrate (alga), allowing sponge regeneration [24]. Sponges were kept within 7–8 specimens in 5 L aquariums, natural seawater, 2–4 °C or 10–12 °C, and transported to the Koltzov Institute of Developmental Biology (Moscow, Russia). Before the experiments, sponges were kept up to 5 days in 5 L aquariums with natural seawater in compliance with the temperature and light/dark cycle of the collection regime. We used 10 specimens of *H*. *panicea* and *H*. *dujardini* collected in August 2019, and 10 specimens of *H*. *dujardini* collected in January 2020. The use of sponges in the laboratory does not raise any ethical issues, and, therefore, approval from regional and local research ethics committees is not required. The field sampling did not involve endangered or protected species. No specific permissions were required for the samplings, locations or activities in accordance with local guidelines.

### 4.2. Sponge Body Dissociation and Reaggregation Procedures

The dissociation/reaggregation experiments were carried out with sponge *H. dujardini* because its body contains mostly the mesohyl cells and has no spicules. The three annual seasons (summer, autumn, and winter) correspond to two periods of the sponge life cycle: the growth period (beginning in August and ending in November) and the beginning of spermatogenesis and oogenesis (January) [66]. Before preparing the cell suspension, the structural and functional integrity of sponges was checked by maintaining the water filtration through the oscula [67]. The dissociated sponge cells were cultivated in compliance with the temperature collection regime in the filtered seawater (FSW) sterilized with the Millex-GP syringe filter units 0.22 μm (Merck KGaA, Darmstadt, Germany), as described earlier [24]. In order to obtain reliable data, cell suspensions and cell aggregates were obtained from the intact body tissue of the same specimens.

### 4.3. DNA Libraries

#### 4.3.1. DNA Isolation, Genomic Library Construction and Sequencing

Genomic DNA was isolated from sponge cells using Qiagen DNA Mini kit (Qiagen, Hilden, Germany) following the manufacturer’s instructions. The concentration of isolated DNA was quantified using Implen NP 40 nanophotometer (Implen, Munich, Germany) or Qubit 3.0 fluorometer (Thermo Fisher Scientific, Waltham, MA, USA). Total DNA (500 ng) was fragmented using Covaris M220 Ultrasonicator (Covaris, Woburn, MA, USA), followed by the preparation of DNA library using NEBNext Ultra DNA Library Prep Kit for Illumina (New England Biolabs, Ipswich, MA, USA) according to the manufacturer’s instructions. Both efficiency of DNA fragmentation and DNA library preparation were controlled using a 2100 Bioanalyzer (Agilent Technologies, Santa Clara, CA, USA) and a High Sensitivity DNA Kit (Agilent Technologies, Santa Clara, CA, USA). Library sequencing was performed using Hiseq 2500 (Illumina, San Diego, CA, USA) with paired-end (2 × 250 bp read length) reads.

#### 4.3.2. Draft Genome Assembly

A library of approximately 36 million paired-end DNA reads (150 + 150, insert size about 450 bp) gave us about 44-x genome coverage, as it was estimated by Kmergenie [68]. The total genome size was estimated as 194 Mb. Draft genome assembly was performed using MaSuRCA assembler, v. 3.3.0 [69]. In order to remove assembly heterozygosity, Redundans pipeline [70] was used. Statistical metrics of continuity and completeness of the resulting assembly were calculated using Quast v. 4.6.3 [71] and Busco v. 4.0.6 [72].

#### 4.3.3. Genomic Features Identification

Gene predictions were made with Augustus v. 3.3.3 [73], GeneMark ES v. 4.62 [74], and MAKER v. 3.01.03 [75] software, using de novo transcriptomes for the support of gene models. CpG islands were searched using the Cpgplot program from the EMBOSS suite v. 6.5.7 [76]. Motifs of DNA regulatory elements were taken from the literature [77,78,79,80,81,82,83,84,85,86,87,88,89,90,91] (detailed references are listed in Supplementary Table S2) and JASPAR database [92] using nucfuzz program from the EMBOSS suite v. 6.5.7 [76]. Iron-responsive elements (IREs) in mRNAs were predicted using the SIREs web-server [93].

### 4.4. RNA Libraries

#### 4.4.1. RNA Isolation

Total RNA was isolated from the sponge body, cell suspensions or cell aggregates with a TRI Reagent (Molecular Research Center, Inc., Cincinnati, OH, USA) according to the manufacturer’s instructions.

#### 4.4.2. cDNA Library Construction, Quality Detection, and Illumina Sequencing

The RNA was purified, the libraries were constructed and sequenced, as described earlier [24].

#### 4.4.3. Differential Expression Analysis for *H. dujardini* Dissociated and Reaggregated Cells

Single-end reads for the three reaggregation states (intact sponge body tissues, dissociated cells, and aggregates) and three annual periods (Winter, Summer, and Autumn) were mapped to the transcriptome assembly and the read counts were quantified with RSEM v. 1.3.1 [94]. The expression correlation matrix was plotted to check the expression consistency between samples, and Autumn replicates tissue#3 and cells#3 were excluded from the subsequent analyses as they deviated significantly from their counterparts (Supplementary Figure S6). The PCA plot of RNA-Seq samples using top-2000 differentially expressed transcripts was made by plotPCA() function of DESeq2 R package [95]. The R package edgeR v. 3.34 [96] was used to analyze differential expression between reaggregation states and growth stages. These two factors, each having three values, give nine combinations total. We chose the grouped model design ‘~0+Group’ with contrasts based on the growth stage (e.g., ‘Winter dissociated cells vs. Winter tissues’) instead of the general two-factor design ‘~0+season*cond’ since its interaction term took over the most part of significance thus complicating the interpretation. First, low-expressed genes were filtered out, resulting in 179,966 out of 357,155 transcripts left for downstream analyses. After library size recalculation and TMM-normalization, the dispersion parameters were estimated (BCV = 0.73) and GLM Quasi-Likelihood model was fitted. *p*-values were BH-corrected and 0.001 was used as a threshold. The R package ComplexHeatmap v.2.7.7 [97] was used to visualize differential expression for curated gene sets.

#### 4.4.4. RACE Analysis

Full-size *H. dujardini* ferritins cDNA flanked by adapter sequences was obtained using Mint technology with the Mint RACE cDNA amplification set (Eurogen, Moscow, Russia) [98]. The first cDNA strand was used in PCR (Step-Out PCR) [99] with a specific primer and a Step-Out primer mix kit (Eurogen, Moscow, Russia). The resulting PCR products were cloned into the pAL2-T plasmid (Eurogen, Moscow, Russia) and sequenced.

#### 4.4.5. Identification of Bacterial Ferritin Superfamily Members

The representatives of bacterial ferritin superfamily members were found in *H. dujardini* transcriptome using *diamond-blastp* search [100] against all the members of bacterial ferritin superfamily present in NCBI protein database. Alignment and tree were constructed in Mega-X software [101] using a maximum likelihood algorithm with default parameters.

### 4.5. Protein Analyses

#### 4.5.1. Native Gel Electrophoresis

Electrophoresis of sponge proteins in a native polyacrylamide gel followed by detection of ferritin in the gel was performed, as described earlier [102] with some modifications. Sponge specimens were homogenized in two volumes of buffer containing 20 mM EDTA, 50 mM Hepes-Na, pH 7.2, 200 mM NaCl. All procedures were performed at 4 °C. After adjustment of the Bis-Tris-glycine buffer concentration to 50 mM, the homogenate was centrifuged at 16,100× *g* for 1 h, and the supernatant was collected. Sucrose solution (50% in water), 0.1 volume, was added, and a 10-μL portion from the sample was loaded onto a polyacrylamide gel. The discontinuous gel (100 × 80 × 0.75 mm^3^) was divided into two portions, the upper one (10 mm) contained 3% polyacrylamide, the lower portion (90 mm) contained a gradient of 3.5–10% polyacrylamide in 50 mM Bis-Tris-glycine, pH 8.0, 10 mM EDTA. The gradient gel was stabilized by a gradient of 0–8% sucrose. The electrophoresis was carried out in a buffer containing 50 mM Bis-Tris-glycine, pH 8.0, 1 mM EDTA at 60 V for 14 h (this step provides optimum resolution from microsomes), 140 V for 8 h and then at 260 V for 14 h. Extended electrophoresis allowed protein complexes to reach positions in the polyacrylamide gradient according to their masses. The electrophoresis was terminated when two colored and differently charged thyroglobulin markers labelled with Cy-3.5 (Lumiprobe, Hunt Valley, MD, USA) reached nearly identical positions in the gel. For detection of ferritin absorbance, the gel was soaked with 0.5 M Bis-Tris-HCl, pH 7.0 at 37 °C. Ferritin has a broad absorption band in the ultraviolet region that tails into the visible region of the spectrum [103,104]. The absorbance under illumination at 365 nm was used to monitor ferritin in the native gel of sponge body tissue homogenates and photographed in a dark room.

#### 4.5.2. Iron Staining

To analyze the ferritin complexes structure and their iron-binding capacity, the sponge body tissue homogenates were subjected to a native gel electrophoresis followed by Prussian blue staining. The Prussian blue staining was performed as described [105], except the staining solution was 2% HCl. Horse spleen ferritin (Merck KGaA, Darmstadt, Germany) was used as a control for gel electrophoresis.

#### 4.5.3. Matrix-Assisted Laser Desorption/Ionization Time-of-Flight Mass (MALDI-TOF)

The ferritin bands were isolated from the native or SDS containing polyacrylamide gels stained with Coomassie blue. Mass spectra of the tryptic peptides were obtained by using the matrix-assisted laser desorption/ionization (MALDI) time-off light mass (TOF) spectrometer Ultraflextreme (Bruker, Billerica, MA, USA), equipped with a UV laser (Nd) and reflectron. Briefly, proteins of interest in pieces (2 × 2 mm^2^) from a polyacrylamide gel were washed twice in 100 μL 40% acetonitrile in 0.1 M NH_4_HCO_3_, once in 100 μL of acetonitrile and were hydrolyzed with 4 μL of the modified trypsin (Promega, Madison, WI, USA) (15 μg/mL in 0.05 M NH_4_HCO_3_) at 37 °C for 18 h. After mixing with 7 μL 0.5% trifluoroacetic acid (TFA), the portion of 0.5 μL from the sample was mixed with 0.5 μL 2.5-dihydroxy benzoic acid (Merck KGaA, Darmstadt, Germany) 20 mg/mL in 30% acetonitrile and 0.5% TFA, spotted on a MALDI plate and air-dried. The monoisotopic mass of the tryptic peptides as positive ions was measured with an accuracy of 30 ppm. The spectra of peptide fragmentation were obtained in the Lift mode with the accuracy of 1 Da for daughter ions. Identification of sponge proteins was performed by using Mascot software v.2.2.2 (Matrix Science Ltd., London, UK) in the *H. dujardini* and *H. panicea* transcriptomes, NCBI database and database of invertebrate EST taking into account possible oxidation of methionines and modification of cysteines by acrylamide.

#### 4.5.4. Liquid Chromatography-Tandem Mass Spectrometry (LC-MS/MS)

The bands with electrophoretic mobility of the ferritin complex (above 440 kDa) were cut from the gel, subjected to tryptic digest and analyzed by nanoLC-MS/MS as described previously [106]. Briefly, peptides extracted by two washes with 0.5% formic acid in 100% acetonitrile, dried down in a Speed Vac Concentrator (Eppendorf, Hamburg, Germany), and resuspended in 20 μL of 0.1% formic acid (FA) in water. The tryptic peptide fraction (injection volume 2 μL) was analyzed in triplicate on a nano-HPLC Agilent 1100 system (Agilent Technologies, Santa Clara, CA, USA) coupled to a 7 T LTQ-FT Ultra mass-spectrometer (Thermo Electron, Bremen, Germany) using a nanospray ion source (positive ion mode, spray voltage +2.3 kV). HPLC separation was performed on a homemade capillary column (75 μm id × 12 cm fused silica capillary filled with Reprosil-Pur Basic C18, 3 μm, 100 Å; Dr. Maisch HPLC GmbH, Ammerbuch-Entringen, Germany) at a flow rate of 0.3 μL/min by gradient elution with the mobile phase A being 0.1% formic acid in water and mobile phase B, 0.1% formic acid in acetonitrile. After pre-equilibration with 3% (*v/v*) solvent B, a 30 min linear gradient from 3% to 50% was applied, followed by a 5 min gradient from 50% to 90% and then a 10 min isocratic elution with 90% solvent B. MS and MS/MS data were obtained in data-dependent mode using Xcalibur (Thermo Finnigan, San Jose, CA, USA) software. The precursor ion scan MS spectra (m/z range 300–1600) were acquired in the FTICR with resolution R = 50,000 at m/z 400 (number of accumulated ions: 5 × 10^6^). Five most intensive ions from each parent scan were isolated and fragmented in the LTQ by collision-induced dissociation (CID) using 3 × 10^4^ accumulated ions. Dynamic exclusion was used with a 30 s duration period.

#### 4.5.5. Data Analysis and Proteins Identification after LC-MS/MS

The resulting data were searched against a sponge transcriptome database (*H. dujardini* (PRJNA594150) and *H. panicea* (PRJNA594151), using the Mascot v.2.2.2 search engine (Matrix Science Ltd., London, UK) and PEAKS Studio 8.5 software packages against the Uniprot KB database. In all searches, the initial mass tolerance for full scans was set to 15 ppm and a mass tolerance of 0.3 Da was set for the precursor and product ions. In a more focused analysis, the mass spectrometry data were searched against a database consisting of only the two sponge ferritins. Possible natural and artefact modifications, such as oxidation of glutamine residues were considered as variable modifications for the first database search. Additionally, a separate modification search with a wide range of possible modifications was carried out. A maximum of up to 10 variable modifications was allowed. The cut-off false discovery rate (FDR) was set to 0.1%. At least one unique peptide and one or two identification peptides per protein were required. Label-free quantitative analysis was performed using the Q module in PEAKS studio in order to determine the significant protein hits distinguishing the separate gel bands.

#### 4.5.6. Ferritin, Actin, and Tubulin Immunofluorescent Microscopy and Cell Imaging

The immunofluorescent staining with polyclonal rabbit antibody to HF (ferritin heavy chain 1) (4393, Cell Signaling Technology, Danvers, MA, USA) was used to examine the ferritin subcellular localization in the *H. dujardini* samples. Staining with the monoclonal mouse antibody against alpha-tubulin (T9026, Sigma-Aldrich/Merck KGaA, Darmstadt, Germany) was used for flagella detection and with F-actin (phalloidin) (A22283, Alexa Fluor 546, Thermo Fisher Scientific, Waltham, MA, USA) for detection of cell edges. The cells were mounted on the glasses and fixed by 4% paraformaldehyde solution (on filtered seawater) for 2–4 h and then were consecutively incubated in 0.5% Triton X-100 with 5% fetal bovine serum in PBS for 40 min at 20 °C; then with the first polyclonal rabbit antibodies to HF (1:500) prepared in PBS with the addition of 0.5% Triton X-100 and 5% FBS for 18 h at 8–10 °C; then they were washed for 10 min three times in PBS and incubated with the second antibodies Alexa Fluor 488 goat anti-rabbit IgG (Invitrogen/Thermo Fisher Scientific, Waltham, MA, USA) (1:700), then after several washes in PBS were incubated with antibody against alpha-tubulin (1:1000) prepared in PBS with the addition of 0.5% Triton X-100 and 5% FBS for 18 h at 8–10 °C; then they were washed for 10 min three times in PBS and incubated with the second antibodies Alexa Fluor 633 donkey anti-mouse IgG (Invitrogen/Thermo Fisher Scientific, Waltham, MA, USA) (1:800) prepared in PBS with the addition of 0.3% Triton X-100 and 5% fetal bovine serum for 2 h at 20 °C, also F-actin (phalloidin) was added in this period. Alternatively, when the actin staining was not needed, the cells were fixed by methanol at −20 °C for 10 min and post-fixed by 3% paraformaldehyde at +4 °C for 30 min without additional permeabilization. The Hoechst-33342 (Invitrogen/Thermo Fisher Scientific, Waltham, MA, USA) staining for the nucleus was done for 5 min after incubation with the last second antibodies. Then, the slides were washed in PBS and placed in Mowiol (Calbiochem/Merck KGaA, Darmstadt, Germany) for analysis using an Olympus IX71 microscope (Olympus, Tokyo, Japan) supplied with a 14-bit CCD-camera Olympus XM10 (Olympus, Tokyo, Japan) and the corresponding licensed CellSense software (Olympus, Tokyo, Japan); the microscope was equipped with a shutter controlled by micro-manager software. Fluorescence images of the cells were obtained by using fluorescence microscopy (Leica DM RXA2, Leica Camera AG, Wetzlar, Germany) at λexc = 495 nm and λem = 517 nm for HF, at λexc = 520 nm and λem = 560 nm for phalloidin, as well as at λexc = 618 nm and λem = 633 nm for alpha-tubulin. The specificity of the first antibodies was confirmed by control experiments when the procedure was performed in their absence. For immune staining of nuclei, the cell suspension in FSW at a concentration of 1 × 10^6^ cells per 1 mL was treated with freshly made formaldehyde solution added to a final concentration of 1 % (*v*/*v*) under incubation at room temperature for 10 min while mixing. The reaction was quenched by the addition of 2.5 M glycine solution to a final concentration of 0.2 M. After incubation at room temperature for 5 min on a rocker, the suspension was centrifuged for 5 min at 300× *g* and 4 °C, and the supernatant was discarded into several slides for 10–15 min. The nuclei were processed for the FTH1 immune staining, as described above. The confocal images of the nuclei were obtained using the Carl Zeiss LSM 880 microscope (Carl Zeiss AG, Oberkochen, Germany) and manufacturer’s software Zen blue.

#### 4.5.7. Transmission Electron Microscopy with Iron Detection

For electron microscopy, the sponge body cells placed on coverslips were fixed in 2.5% glutaraldehyde in seawater filtered using Millipore membrane (instead of 0.1 M Sörensen’s phosphate buffer) for 1.5 h. After rinsing 3 times in filtered seawater, the samples were postfixed in 1% OsO_4_, dehydrated according to standard techniques and embedded in Epon 812 (Fluka/Sigma-Aldrich Holding AG, Buchs, Switzerland). Ultrathin (70 nm) sections were stained with uranyl acetate. Transmission electron microscopy was performed with JEM-2100 200 kV electron microscope (JEOL, Tokyo, Japan) equipped with LaB6 electron source. Electron energy loss spectroscopy data for the Fe L2,3 peak were obtained with GIF Quantum ER spectrometer (Gatan, Inc., Pleasanton, CA, USA) in STEM mode. The spectrum acquisition parameters were set up to maximize the signal to noise ratio while reducing the specimen contamination and shrinkage due to radiation damage. EELS spectra were acquired for each 20 × 20 nm STEM pixel with 0.25 eV spectrometer dispersion, 6 mrad collection angle and 708 eV energy shift. Each spectrum was background-subtracted with a power-law background window, positioned at 680–705 eV region. Plural scattering effects corrected with Fourier-ratio deconvolution with zero-loss spectrum data were obtained from the same STEM area. The signal window for the Fe mapping was set to 708–758 eV. Images and spectra were obtained and processed with Gatan Digital Micrograph software (Gatan, Inc., Pleasanton, CA, USA).

### 4.6. Functional Annotation of Proteins

#### 4.6.1. Multiple Sequence Alignment and Tree Construction

All protein sequences of ferritins were aligned together using the clustalo algorithm [43]. Before phylogenetic tree construction, the alignment was processed with the Gappyout algorithm using trimal v. 1.2 software [44], leaving 132 core residues in the alignment. The tree was constructed using Maximum likelihood approach with 1000 fast bootstrap resamplings with IQ-Tree v. 1.6.12 [45] and visualized using iTOL server [46] with ferritin sequences of *Homo sapiens* used as a root. Visualization of protein alignments with secondary structure elements was made with ESPript web-server v. 3.0 [107] and JalView v. 2.11 [108].

#### 4.6.2. Homology Modeling

Homology modeling was performed using the SWISS-MODEL web-server [41] using a structure of *Sinonovacula constricta* ferritin (PDB: 6LP5) as the main template. Template search with BLAST and HHBlits has been performed against the SWISS-MODEL template library [109,110]. Only wild-type structures with iron atoms bound were considered as templates. Additional modeling was performed using the I-TASSER web-server [111]. The global and per-residue model quality has been assessed using the QMEAN scoring function [112]. Molecular graphics was prepared based on SWISS-MODEL modeling result using UCSF Chimera software v. 1.15 [42].

#### 4.6.3. Sequence Features Mining and Selection

Amino acid sequences of invertebrate and human non-mitochondrial ferritins were downloaded from the NCBI protein database using Entrez Direct software v. 15.0. [113]. Several categories of sequences were removed:

1. Highly similar sequences having more than 95% identity after clustering by CD-HIT v. 4.8.1 [114];
2. Manually selected sequences which introduced long singleton regions to the total alignment;
3. Sequences annotated as partial and of low-quality;
4. Sequences shorter than 150 or longer than 300 amino acids;
5. Sequences of Daphnia magna species (more than 40 sequences annotated as ferritins);
6. Sequences that have only coding mRNA sequence without UTRs (so it is impossible to screen it for iron-responsive elements).

In order to represent invertebrate phyla more uniformly, ferritin sequences of several organisms were added manually from various transcriptomic and genomic assemblies [22,23,37,55,60,115,116,117,118,119,120,121,122,123,124,125,126,127,128,129,130,131,132,133,134,135,136,137,138,139,140,141,142,143,144,145,146,147] using blastp from NCBI BLAST+ package v. 2.10 [109] and exonerate v. 2.2 [148] searches (see detailed references in Supplementary Table S1). An additional search was done in Ctenophore databases [149]. xRGG motif enrichment analysis was performed using FIMO tool from MEME Suite v. 5.3.3 [150]. Signal peptides were predicted with SignalP software v. 5.0 [151]. The percentage of identity and similarity in global pairwise alignments with HuHF was calculated with Needle tool from EMBOSS suite v. 6.6 [76]. Molecular weights and isoelectric points were predicted using ExPASy web-server [152]. Transient heme binding was predicted using HeMoQuest web-server [153]. Protein sequences’ features were mined with iLearnPlus software [154]. The feature set was reduced by uniting the top 100 features obtained from each of five unsupervised feature selection algorithms implemented in scikit-feature Python package [155]: Laplacian score, SPEC, MCFS, NDFS, and UDFS. The PCA dimensionality reduction over remaining feature space was performed using built-in R function *prcomp()*.

### 4.7. Statistical Analyses

The relative amount of HdF1a/b in the nucleus versus cytoplasm was estimated by measuring the fluorescence level after immunostaining of cells with anti-rabbit HF antibodies. The boundaries of the nucleus in each cell were circled in Hoechst-33342 channel, and the average fluorescence and maximum fluorescence level of HdF1a/b were measured in this region. Then, the region of cytoplasm outside the nucleus was circled using the phalloidin channel, and both the average and maximum fluorescence of HdF1a/b were measured. The non-parametric data on ferritin fluorescence were presented as median, minimum, and maximum values. A comparison between the group samples was performed using Mann Whitney’s test. All other data were parametric and shown as mean and standard deviation. Comparisons between the groups were performed by using the ANOVA variance test, followed by Tukey’s test. The level of significance was set to 1%.

## 5. Conclusions

This study revealed that the major ferritins *HdF1a/b* and *HpF1* of the sea cold-water sponges *H. dujardini* and *H. panicea* belong to the most highly expressed genes at transcriptional and proteome levels in sponge cells. The iron-binding HdF1a/b and atypical HdF2 ferritins are differentially expressed during reaggregation and hence participate in the regulation of morphogenetic processes in sponges. Transient heme binding and the absence of iron-binding activity at the ferrihydrite nucleation site are predicted for all studied sponge ferritins. The *H. dujardini* minor ferritin HdF2 and the freshwater sponge *E. muelleri*’s EmF2 exemplify atypical ferritins which are widely distributed among invertebrate species, regulated differently due to lack of iron-responsive elements, and still have unknown function. The roles of ferritins in ancient multicellular species, sea sponges, deserve further research.

## Data Availability

All relevant data are within the paper and its Supplementary Materials. Draft genomic scaffolds containing *H. dujardini* ferritin genes are available via DOI:10.6084/m9.figshare.15029361.v1. Raw single-end RNA-seq reads of *H. dujardini* are deposited to NCBI SRA database (Sequence Read Archive of the National Center for Biotechnology Information) under accession number PRJNA594150, sample numbers SAMN20337311—SAMN20337327 (see details in Table S6). Previously assembled de novo transcriptomes are available at NCBI TSA database (Transcriptome Shotgun Assembly of the National Center for Biotechnology Information) under accession numbers GIFI00000000.1 (*H. dujardini*) and GIFJ00000000.1 (*H. panicea*), with a curated set of sequences of iron metabolic pathways being deposited directly to GenBank (see details in Table S7). References for transcriptomes and genomes of other species used in the analyses are listed in Table S1.

## Supplementary Materials

The following are available online at https://www.mdpi.com/article/10.3390/ijms22168635/s1: Figure S1: Phylogenetic tree of H. panicea ferritin transcripts along with other ferritins of sponges and model organisms. Figure S2: PCA on a set of animal ferritins built using 474 sequence features selected by unsupervised algorithms (large version). Figure S3: Maximum likelihood phylogenetic tree of invertebrate ferritins (version with all the branches expanded). Figure S4: Extended microscopy images. Figure S5: Heatmap of expression levels of iron metabolic proteins of *H. dujardini*. Figure S6: Expression correlation of RNA-Seq samples of *H. dujardini*. Figure S7: Transmission electron microscopy of the fragment of sponge cell with inclusions in higher resolution. Figure S8: Extended mass-spectrometry results: peptide coverage. Figure S9: Ferritin complexes native gels. Figure S10: Phylogenetic tree of bacterial ferritin superfamily members present in H. dujardini transcriptome. Table S1: Invertebrate ferritins dataset: accession numbers, features, mRNA and protein sequences. Table S2: Regulatory sequences in the 5′ region of the ferritin genes of *H. dujardini*. Table S3: *H. panicea* ferritin variant calls. Table S4: Amino acid sequence features of invertebrate ferritins used for feature selection and PCA. Table S5: Ferritins and iron metabolism proteins found in Ctenophores. Table S6: RNA-Seq samples statistics and accession numbers. Table S7: *H. dujardini* reaggregation experiment: expression table and accession numbers. Table S8: Expression table for bacterial representatives of the ferritin superfamily. Table S9: Spectrum data for identification of the ferric ion-containing granules in *H. dujardini* cells. Table S10: Extended mass-spectrometry results: summary and supporting peptides.
