# Supplementary material for: Conservative and Atypical Ferritins of Sponges
Source: Int J Mol Sci. 2021 Aug 11;22(16):8635. doi: 10.3390/ijms22168635 (PMC8395497; doi:10.3390/ijms22168635)
Supplement: Supplementary file 1 [file ijms-22-08635-s001.zip › suppl_figures/Figure_S01. Phylogenetic tree of H. panicea ferritin transcripts along with other ferritins.pdf]

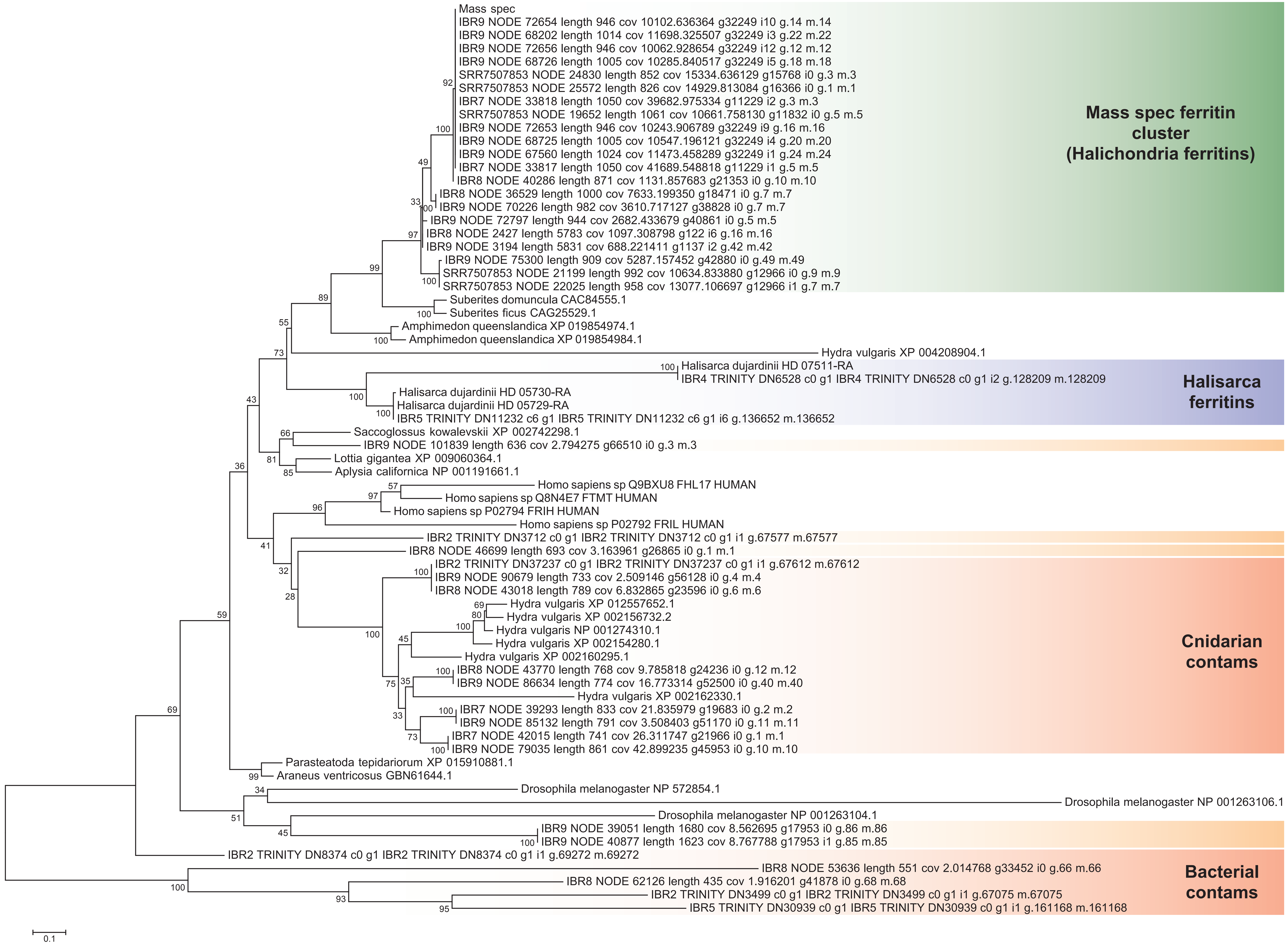
Mass spec
IBR9 NODE 72654 length 946 cov 10102.636364 g32249 i10 g.14 m.14
IBR9 NODE 68202 length 1014 cov 11698.325507 g32249 i3 g.22 m.22
IBR9 NODE 72656 length 946 cov 10062.928654 g32249 i12 g.12 m.12
IBR9 NODE 68726 length 1005 cov 10285.840517 g32249 i5 g.18 m.18
SRR7507853 NODE 24830 length 852 cov 15334.636129 g15768 i0 g.3 m.3
SRR7507853 NODE 25572 length 826 cov 14929.813084 g16366 i0 g.1 m.1
IBR7 NODE 33818 length 1050 cov 39682.975334 g11229 i2 g.3 m.3
SRR7507853 NODE 19652 length 1061 cov 10661.758130 g11832 i0 g.5 m.5
IBR9 NODE 72653 length 946 cov 10243.906789 g32249 i9 g.16 m.16
IBR9 NODE 68725 length 1005 cov 10547.196121 g32249 i4 g.20 m.20
IBR9 NODE 67560 length 1024 cov 11473.458289 g32249 i1 g.24 m.24
IBR7 NODE 33817 length 1050 cov 41689.548818 g11229 i1 g.5 m.5
IBR8 NODE 40286 length 871 cov 1131.857683 g21353 i0 g.10 m.10
IBR8 NODE 36529 length 1000 cov 7633.199350 g18471 i0 g.7 m.7
IBR9 NODE 70226 length 982 cov 3610.717127 g38828 i0 g.7 m.7
IBR9 NODE 72797 length 944 cov 2682.433679 g40861 i0 g.5 m.5
IBR8 NODE 2427 length 5783 cov 1097.308798 g122 i6 g.16 m.16
IBR9 NODE 3194 length 5831 cov 688.221411 g1137 i2 g.42 m.42
IBR9 NODE 75300 length 909 cov 5287.157452 g42880 i0 g.49 m.49
SRR7507853 NODE 21199 length 992 cov 10634.833880 g12966 i0 g.9 m.9
SRR7507853 NODE 22025 length 958 cov 13077.106697 g12966 i1 g.7 m.7
Suberites domuncula CAC84555.1
Suberites ficus CAG25529.1
Amphimedon queenslandica XP 019854974.1
Amphimedon queenslandica XP 019854984.1
Hydra vulgaris XP 004208904.1
Halisarca dujardinii HD 07511-RA
IBR4 TRINITY DN6528 c0 g1 IBR4 TRINITY DN6528 c0 g1 i2 g.128209 m.128209
Halisarca dujardinii HD 05730-RA
Halisarca dujardinii HD 05729-RA
IBR5 TRINITY DN11232 c6 g1 IBR5 TRINITY DN11232 c6 g1 i6 g.136652 m.136652
Saccoglossus kowalevskii XP 002742298.1
IBR9 NODE 101839 length 636 cov 2.794275 g66510 i0 g.3 m.3
Lottia gigantea XP 009060364.1
Aplysia californica NP 001191661.1
Homo sapiens sp Q9BXU8 FHL17 HUMAN
Homo sapiens sp Q8N4E7 FTMT HUMAN
Homo sapiens sp P02794 FRIH HUMAN
Homo sapiens sp P02792 FRIL HUMAN
IBR2 TRINITY DN3712 c0 g1 IBR2 TRINITY DN3712 c0 g1 i1 g.67577 m.67577
IBR8 NODE 46699 length 693 cov 3.163961 g26865 i0 g.1 m.1
IBR2 TRINITY DN37237 c0 g1 IBR2 TRINITY DN37237 c0 g1 i1 g.67612 m.67612
IBR9 NODE 90679 length 733 cov 2.509146 g56128 i0 g.4 m.4
IBR8 NODE 43018 length 789 cov 6.832865 g23596 i0 g.6 m.6
Hydra vulgaris XP 012557652.1
Hydra vulgaris XP 002156732.2
Hydra vulgaris NP 001274310.1
Hydra vulgaris XP 002154280.1
Hydra vulgaris XP 002160295.1
IBR8 NODE 43770 length 768 cov 9.785818 g24236 i0 g.12 m.12
IBR9 NODE 86634 length 774 cov 16.773314 g52500 i0 g.40 m.40
Hydra vulgaris XP 002162330.1
IBR7 NODE 39293 length 833 cov 21.835979 g19683 i0 g.2 m.2
IBR9 NODE 85132 length 791 cov 3.508403 g51170 i0 g.11 m.11
IBR7 NODE 42015 length 741 cov 26.311747 g21966 i0 g.1 m.1
IBR9 NODE 79035 length 861 cov 42.899235 g45953 i0 g.10 m.10
Parasteatoda tepidariorum XP 015910881.1
Araneus ventricosus GBN61644.1
Drosophila melanogaster NP 572854.1
Drosophila melanogaster NP 001263106.1
Drosophila melanogaster NP 001263104.1
IBR9 NODE 39051 length 1680 cov 8.562695 g17953 i0 g.86 m.86
IBR9 NODE 40877 length 1623 cov 8.767788 g17953 i1 g.85 m.85
IBR2 TRINITY DN8374 c0 g1 IBR2 TRINITY DN8374 c0 g1 i1 g.69272 m.69272
IBR8 NODE 53636 length 551 cov 2.014768 g33452 i0 g.66 m.66
IBR8 NODE 62126 length 435 cov 1.916201 g41878 i0 g.68 m.68
IBR2 TRINITY DN3499 c0 g1 IBR2 TRINITY DN3499 c0 g1 i1 g.67075 m.67075
IBR5 TRINITY DN30939 c0 g1 IBR5 TRINITY DN30939 c0 g1 i1 g.161168 m.161168
Mass spec ferritin cluster (Halichondria ferritins)
Halisarca ferritins
Cnidarian contams
Bacterial contams
0.1
