# Supplementary material for: Conservative and Atypical Ferritins of Sponges
Source: Int J Mol Sci. 2021 Aug 11;22(16):8635. doi: 10.3390/ijms22168635 (PMC8395497; doi:10.3390/ijms22168635)
Supplement: Supplementary file 1 [file ijms-22-08635-s001.zip › suppl_figures/Figure_S03. Maximum likelihood phylogenetic tree of invertebrate ferritins (version with all branches uncollapsed).pdf]

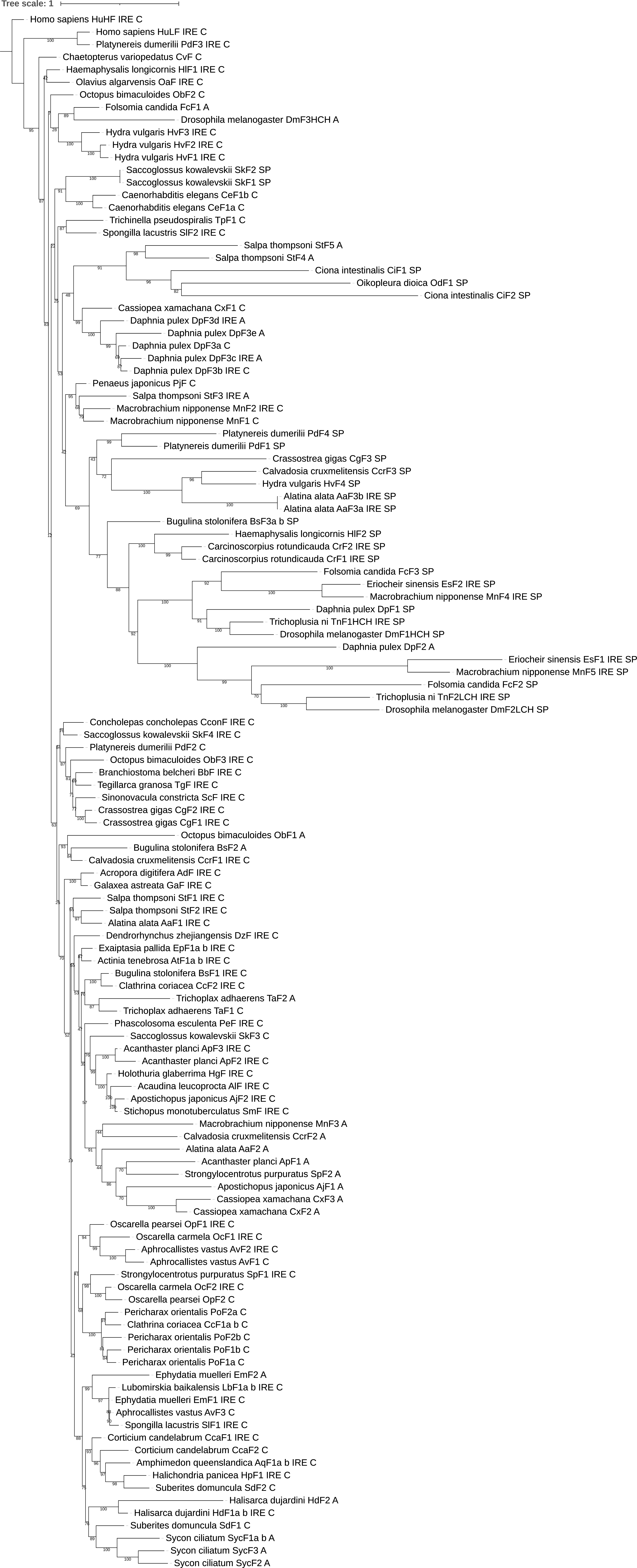
Tree scale: 1
Homo sapiens HuHF IRE C
Homo sapiens HuLF IRE C
Platynereis dumerilii PdF3 IRE C
Chaetopterus variopedatus CvF C
Haemaphysalis longicornis HlF1 IRE C
Olavius algarvensis OaF IRE C
Octopus bimaculoides ObF2 C
Folsomia candida FcF1 A
Drosophila melanogaster DmF3HCH A
Hydra vulgaris HvF3 IRE C
Hydra vulgaris HvF2 IRE C
Hydra vulgaris HvF1 IRE C
Saccoglossus kowalevskii SkF2 SP
Saccoglossus kowalevskii SkF1 SP
Caenorhabditis elegans CeF1b C
Caenorhabditis elegans CeF1a C
Trichinella pseudospiralis TpF1 C
Spongilla lacustris SlF2 IRE C
Salpa thompsoni StF5 A
Salpa thompsoni StF4 A
Ciona intestinalis CiF1 SP
Oikopleura dioica OdF1 SP
Ciona intestinalis CiF2 SP
Cassiopea xamachana CxF1 C
Daphnia pulex DpF3d IRE A
Daphnia pulex DpF3e A
Daphnia pulex DpF3a C
Daphnia pulex DpF3c IRE A
Daphnia pulex DpF3b IRE C
Penaeus japonicus PjF C
Salpa thompsoni StF3 IRE A
Macrobrachium nipponense MnF2 IRE C
Macrobrachium nipponense MnF1 C
Platynereis dumerilii PdF4 SP
Platynereis dumerilii PdF1 SP
Crassostrea gigas CgF3 SP
Calvadosia cruxmelitensis CcrF3 SP
Hydra vulgaris HvF4 SP
Alatina alata AaF3b IRE SP
Alatina alata AaF3a IRE SP
Bugulina stolonifera BsF3a b SP
Haemaphysalis longicornis HlF2 SP
Carcinoscorpius rotundicauda CrF2 IRE SP
Carcinoscorpius rotundicauda CrF1 IRE SP
Folsomia candida FcF3 SP
Eriocheir sinensis EsF2 IRE SP
Macrobrachium nipponense MnF4 IRE SP
Daphnia pulex DpF1 SP
Trichoplusia ni TnF1HCH IRE SP
Drosophila melanogaster DmF1HCH SP
Daphnia pulex DpF2 A
Eriocheir sinensis EsF1 IRE SP
Macrobrachium nipponense MnF5 IRE SP
Folsomia candida FcF2 SP
Trichoplusia ni TnF2LCH IRE SP
Drosophila melanogaster DmF2LCH SP
Concholepas concholepas CconF IRE C
Saccoglossus kowalevskii SkF4 IRE C
Platynereis dumerilii PdF2 C
Octopus bimaculoides ObF3 IRE C
Branchiostoma belcheri BbF IRE C
Tegillarca granosa TgF IRE C
Sinonovacula constricta ScF IRE C
Crassostrea gigas CgF2 IRE C
Crassostrea gigas CgF1 IRE C
Octopus bimaculoides ObF1 A
Bugulina stolonifera BsF2 A
Calvadosia cruxmelitensis CcrF1 IRE C
Acropora digitifera AdF IRE C
Galaxea astreata GaF IRE C
Salpa thompsoni StF1 IRE C
Salpa thompsoni StF2 IRE C
Alatina alata AaF1 IRE C
Dendrorhynchus zhejiangensis DzF IRE C
Exaiptasia pallida EpF1a b IRE C
Actinia tenebrosa AtF1a b IRE C
Bugulina stolonifera BsF1 IRE C
Clathrina coriacea CcF2 IRE C
Trichoplax adhaerens TaF2 A
Trichoplax adhaerens TaF1 C
Phascolosoma esculenta PeF IRE C
Saccoglossus kowalevskii SkF3 C
Acanthaster planci ApF3 IRE C
Acanthaster planci ApF2 IRE C
Holothuria glaberrima HgF IRE C
Acaudina leucoprocta AlF IRE C
Apostichopus japonicus AjF2 IRE C
Stichopus monotuberculatus SmF IRE C
Macrobrachium nipponense MnF3 A
Calvadosia cruxmelitensis CcrF2 A
Alatina alata AaF2 A
Acanthaster planci ApF1 A
Strongylocentrotus purpuratus SpF2 A
Apostichopus japonicus AjF1 A
Cassiopea xamachana CxF3 A
Cassiopea xamachana CxF2 A
Oscarella pearsei OpF1 IRE C
Oscarella carmela OcF1 IRE C
Aphrocallistes vastus AvF2 IRE C
Aphrocallistes vastus AvF1 C
Strongylocentrotus purpuratus SpF1 IRE C
Oscarella carmela OcF2 IRE C
Oscarella pearsei OpF2 C
Pericharax orientalis PoF2a C
Clathrina coriacea CcF1a b C
Pericharax orientalis PoF2b C
Pericharax orientalis PoF1b C
Pericharax orientalis PoF1a C
Ephydatia muelleri EmF2 A
Lubomirskia baikalensis LbF1a b IRE C
Ephydatia muelleri EmF1 IRE C
Aphrocallistes vastus AvF3 C
Spongilla lacustris SlF1 IRE C
Corticium candelabrum CcaF1 IRE C
Corticium candelabrum CcaF2 C
Amphimedon queenslandica AqF1a b IRE C
Halichondria panicea HpF1 IRE C
Suberites domuncula SdF2 C
Halisarca dujardini HdF2 A
Halisarca dujardini HdF1a b IRE C
Suberites domuncula SdF1 C
Sycon ciliatum SycF1a b A
Sycon ciliatum SycF3 A
Sycon ciliatum SycF2 A
