# Supplementary material for: Conservative and Atypical Ferritins of Sponges
Source: Int J Mol Sci. 2021 Aug 11;22(16):8635. doi: 10.3390/ijms22168635 (PMC8395497; doi:10.3390/ijms22168635)
Supplement: Supplementary file 1 [file ijms-22-08635-s001.zip › suppl_figures/Figure_S04. Extended microscopy images.pdf]

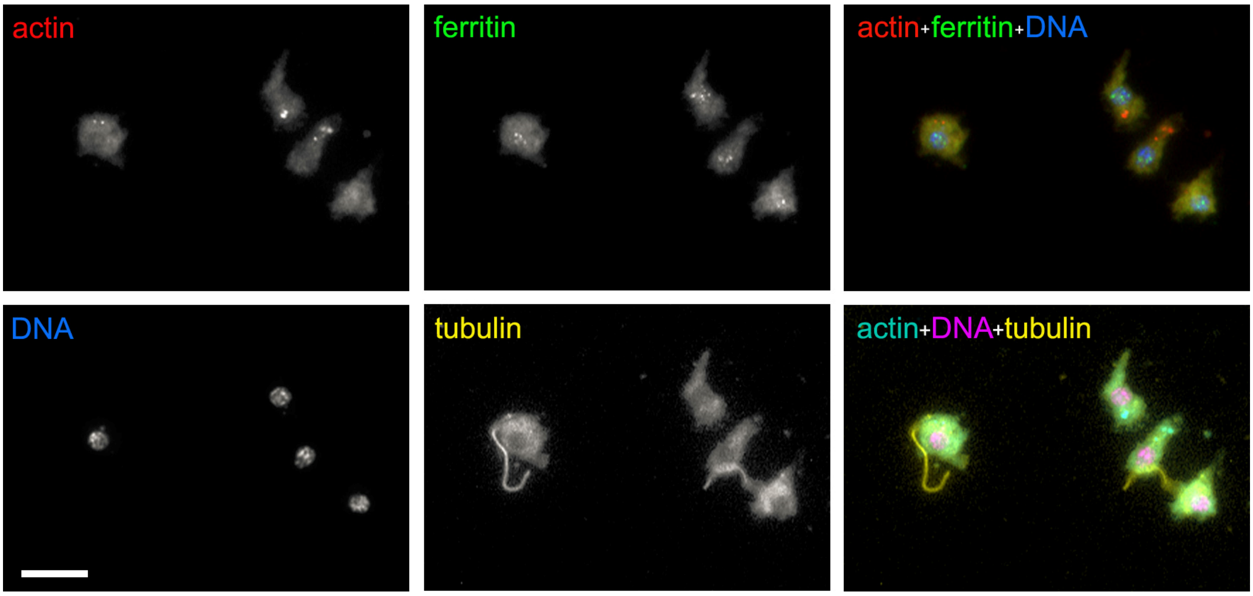

Figure S4A. Confocal microscopy of *H. dujardini* cells, quadruple stained for tubulin, actin, ferritin and chromatin. Scale bar 10 µm.

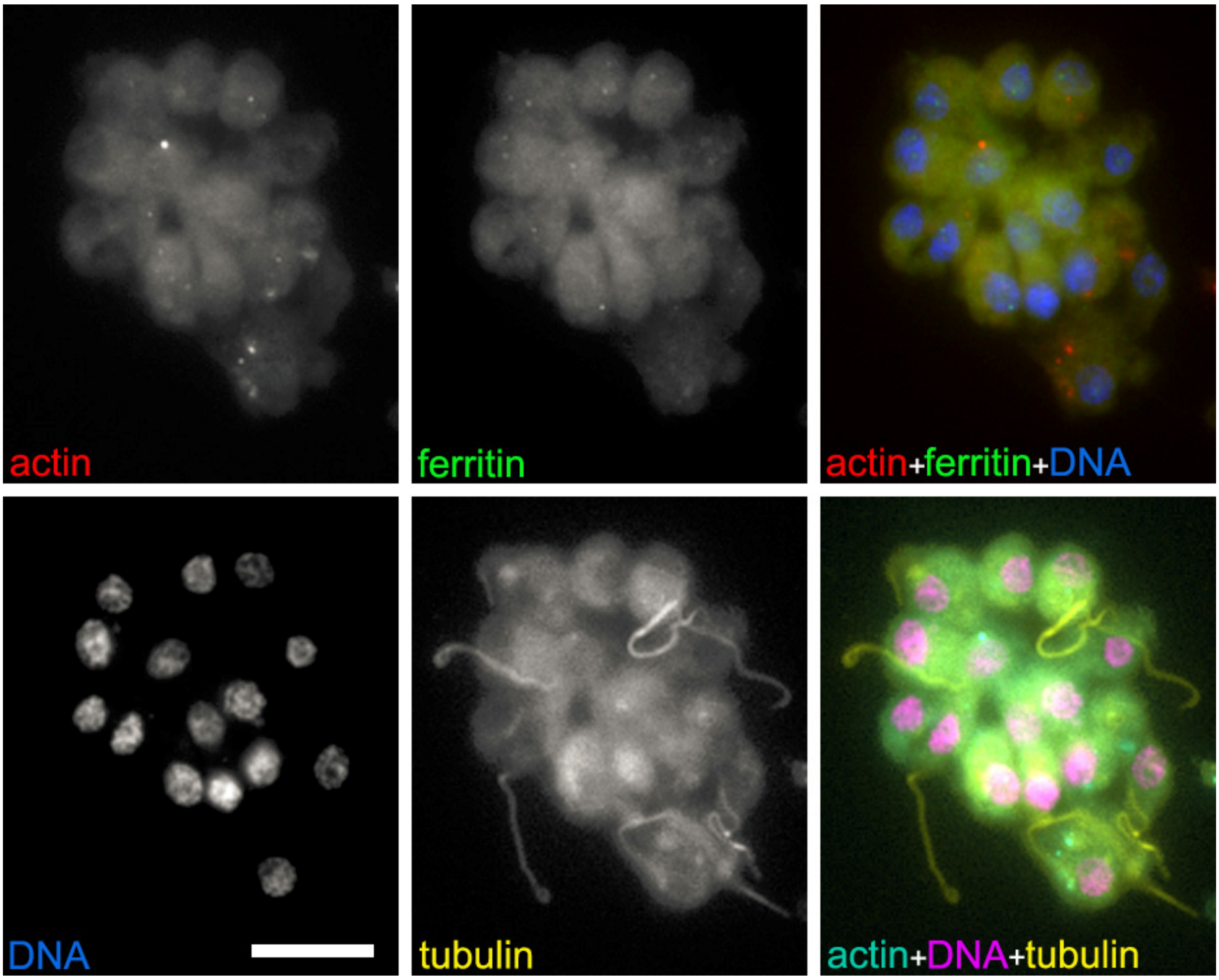

Figure S4B. Confocal microscopy of *H. dujardini* cell aggregates, quadruple stained for tubulin, actin, ferritin and chromatin. Scale bar 10 µm.

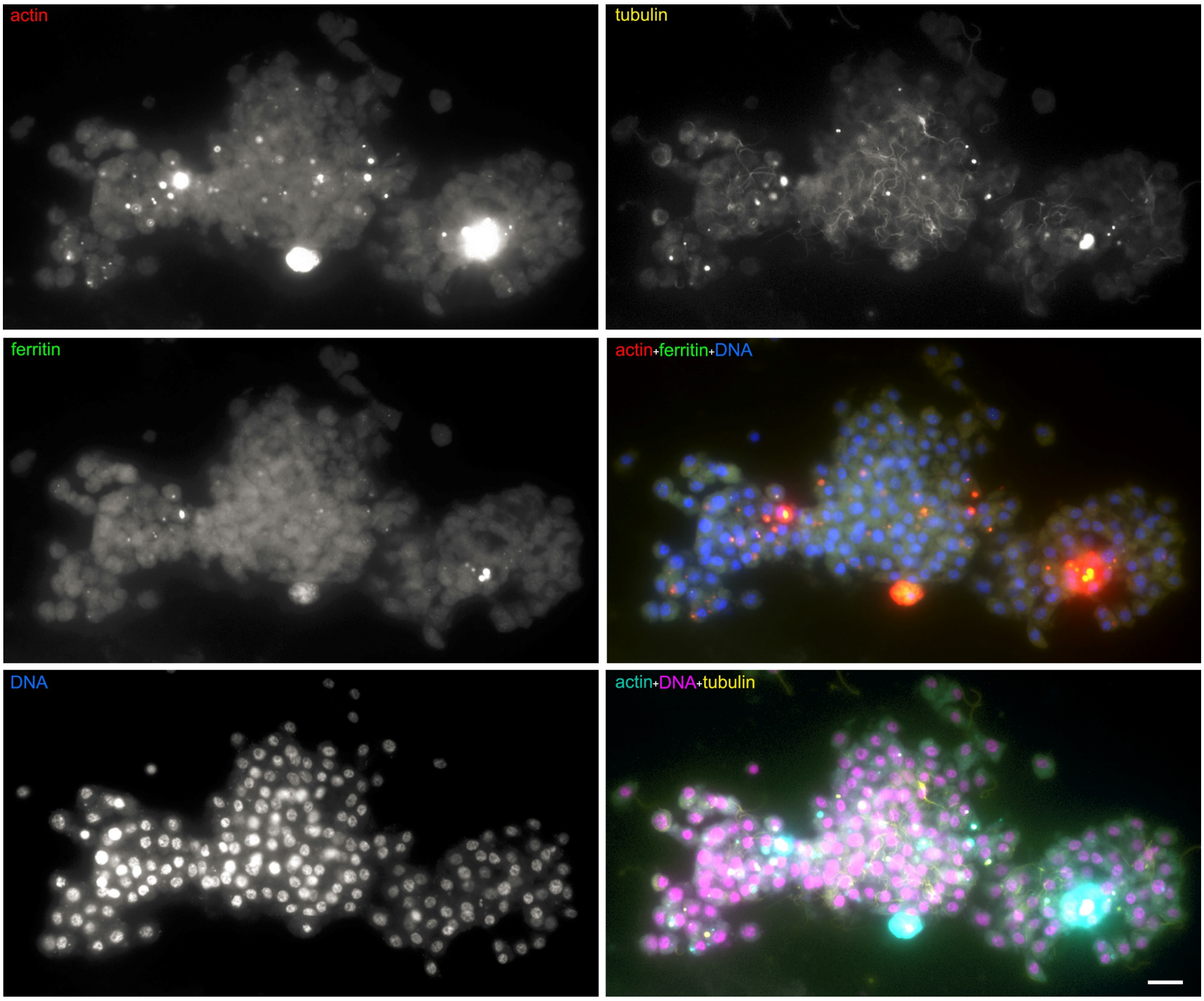

Figure S4C. Confocal microscopy of *H. dujardini* tissues, quadruple stained for tubulin, actin, ferritin and chromatin. Scale bar 10 µm.

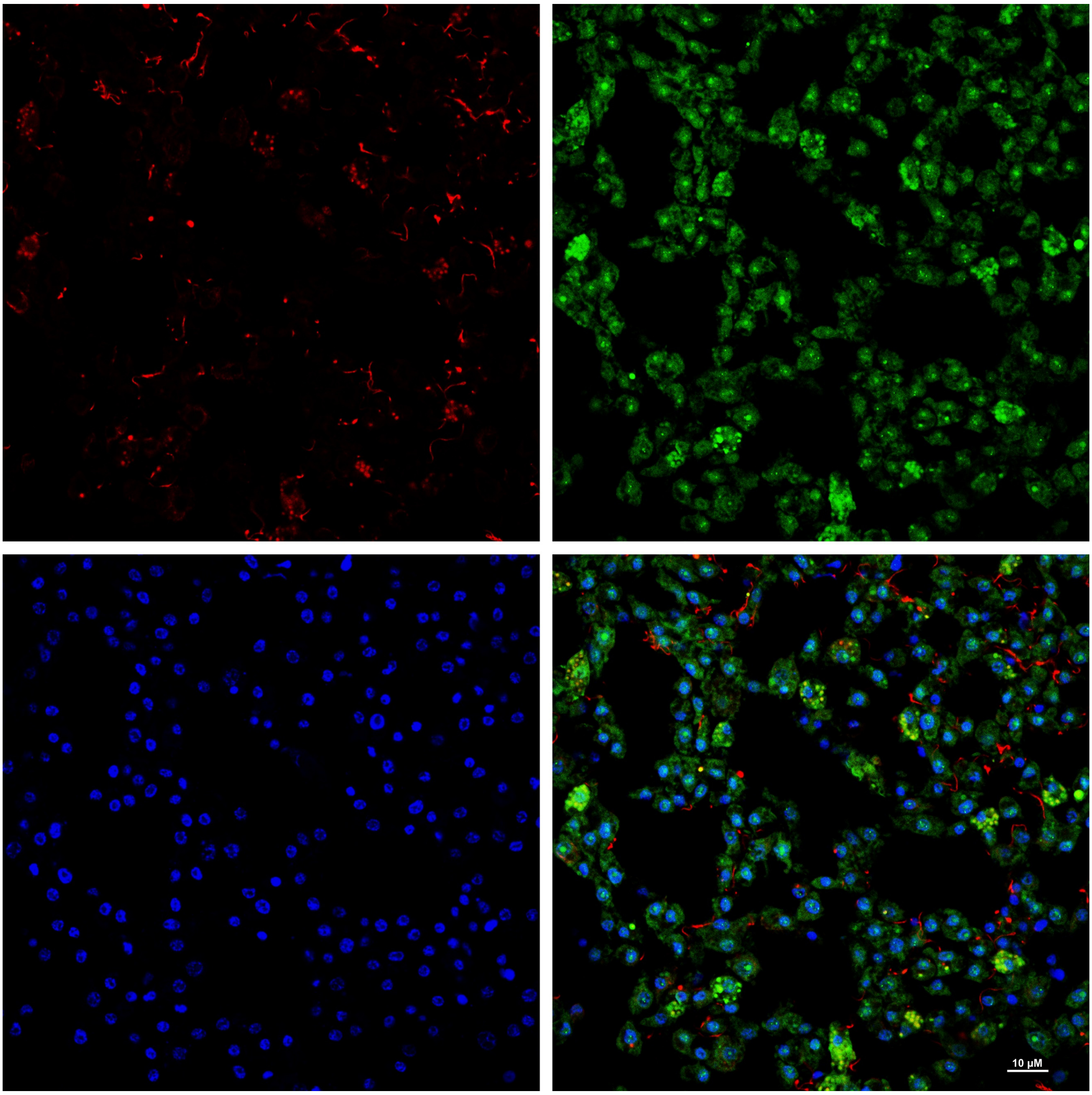

Figure S4D. Confocal microscopy of *H. dujardini* tissues, triple stained for tubulin (red), ferritin (green) and chromatin (blue). Scale bar 10 μm.

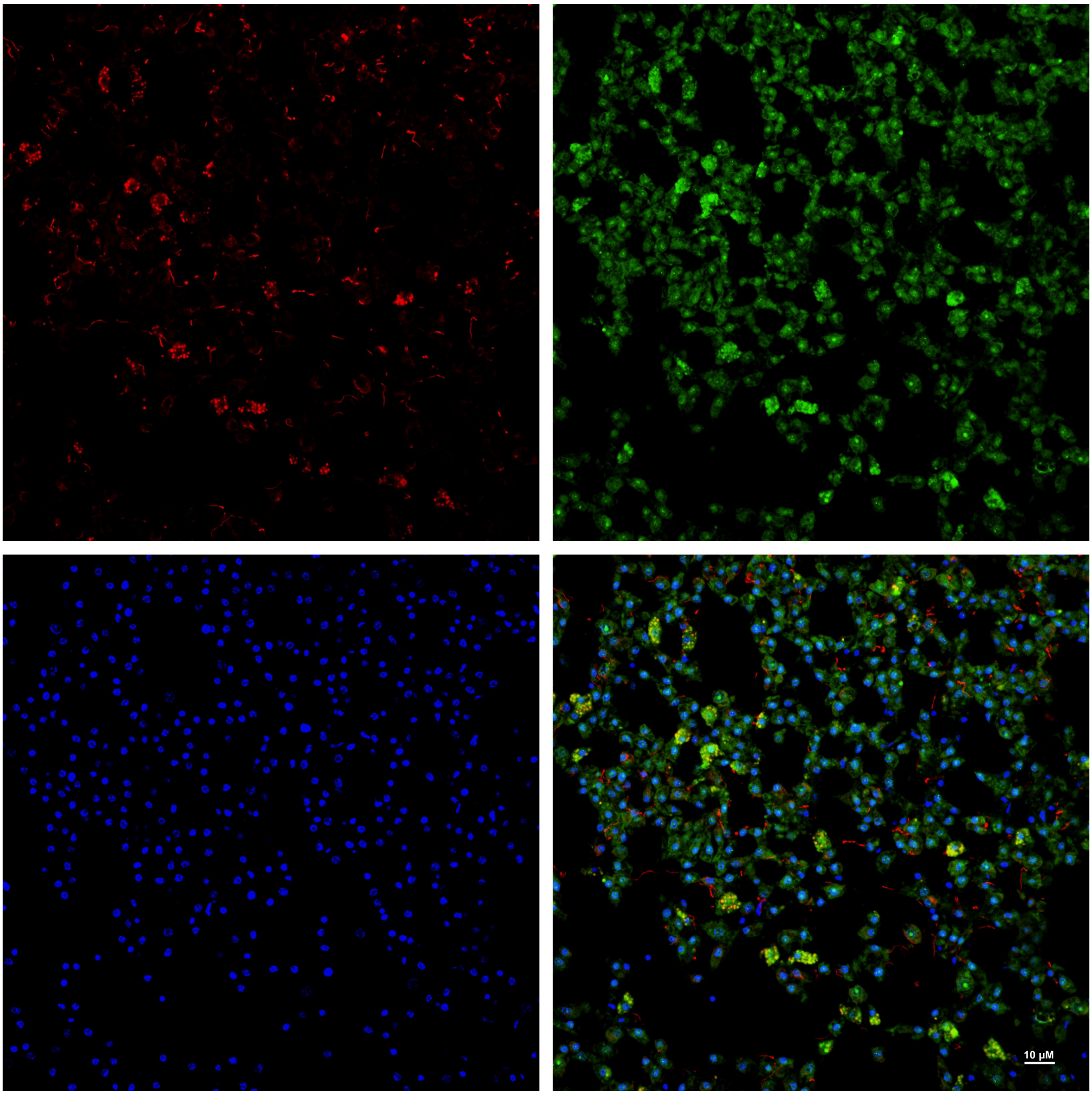

Figure S4E. Confocal microscopy of *H. dujardini* tissues, triple stained for tubulin (red), ferritin (green) and chromatin (blue). Scale bar 10 μm.

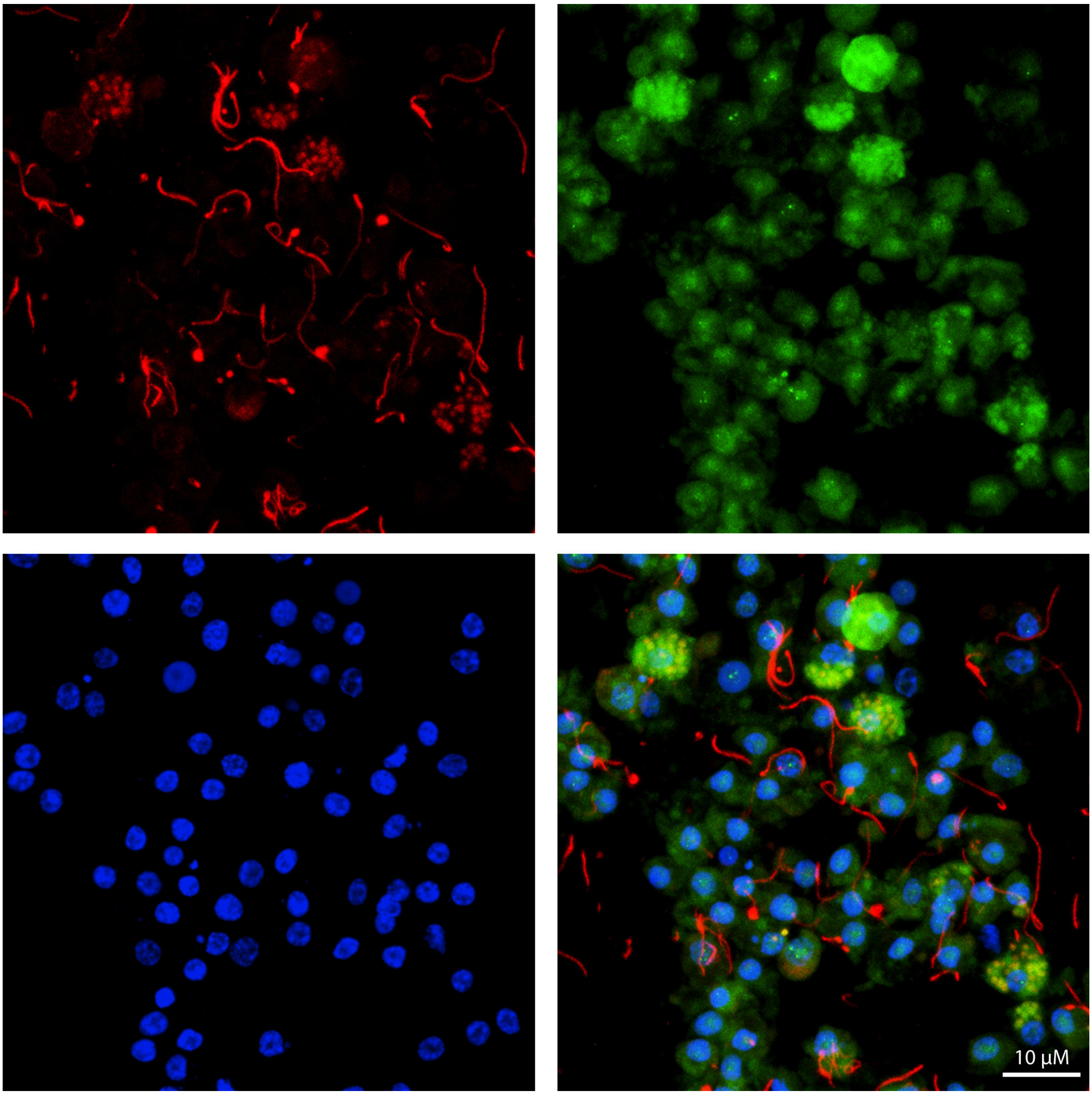

Figure S4F. Confocal microscopy of *H. dujardini* tissues, triple stained for tubulin (red), ferritin (green) and chromatin (blue). Scale bar 10 μm.

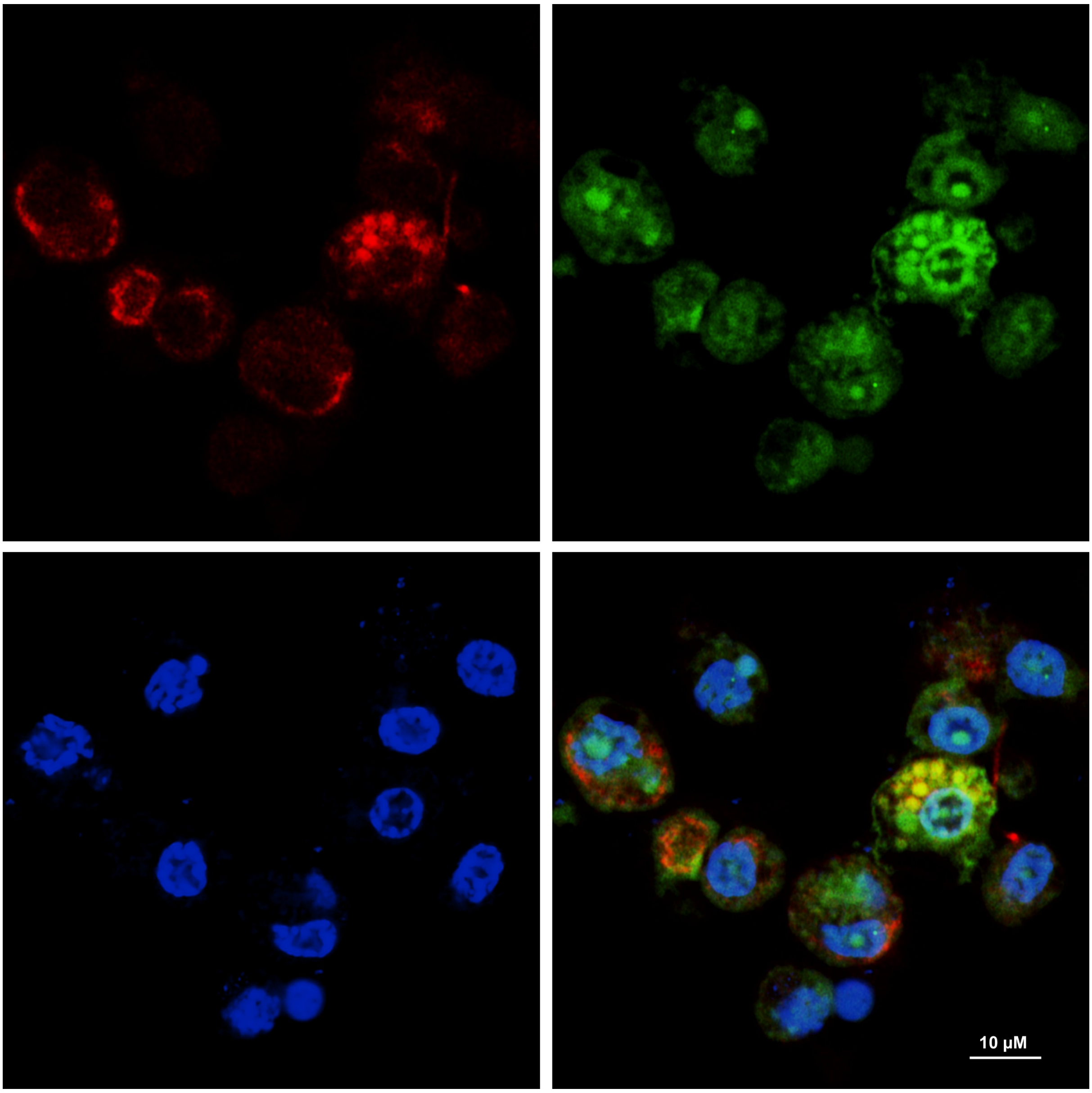

Figure S4G. Confocal microscopy of *H. dujardini* cells, triple stained for tubulin (red), ferritin (green) and chromatin (blue). Scale bar 10 µm.

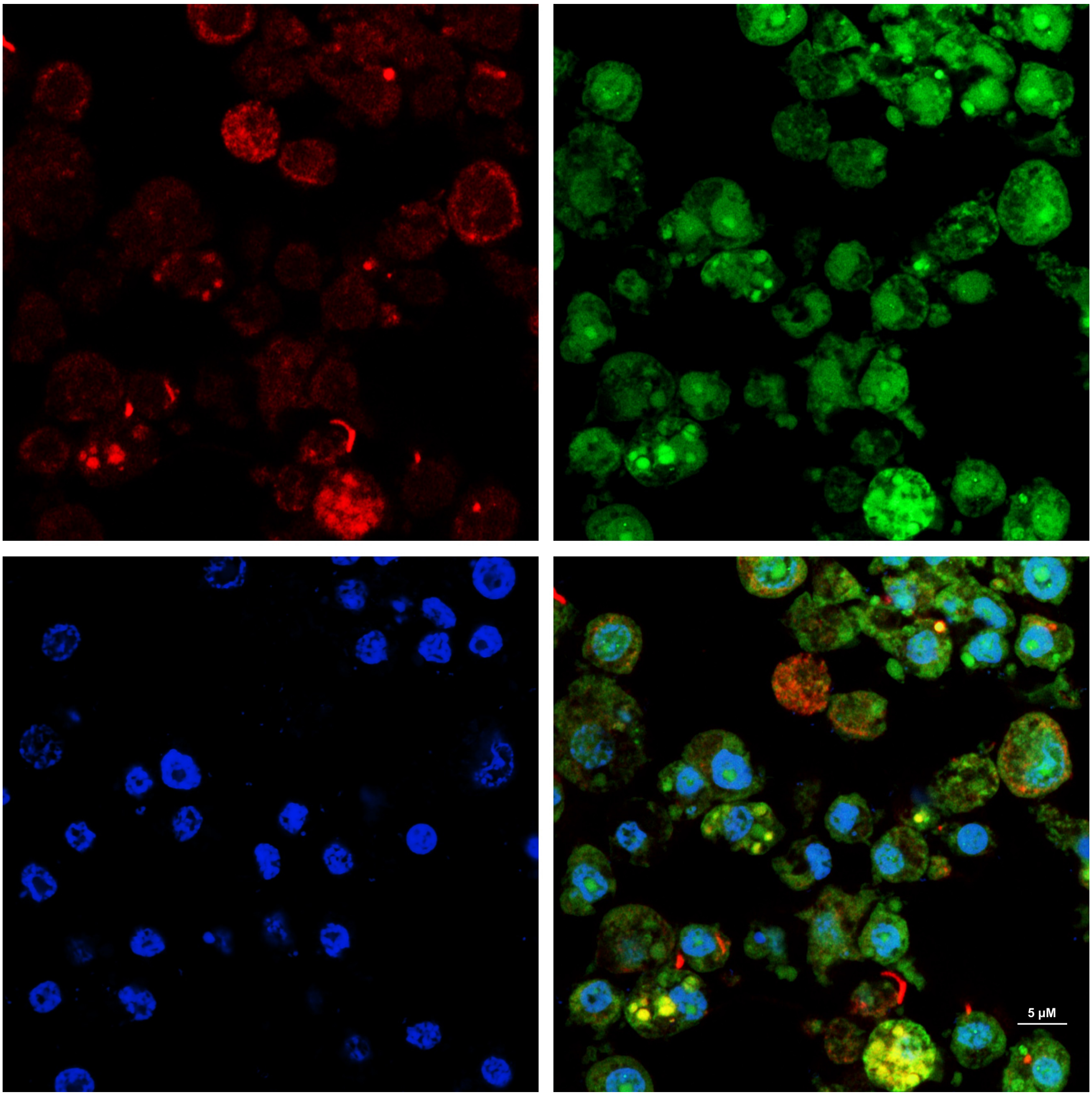

Figure S4H. Confocal microscopy of *H. dujardini* tissues, triple stained for tubulin (red), ferritin (green) and chromatin (blue). Scale bar 5 µm.

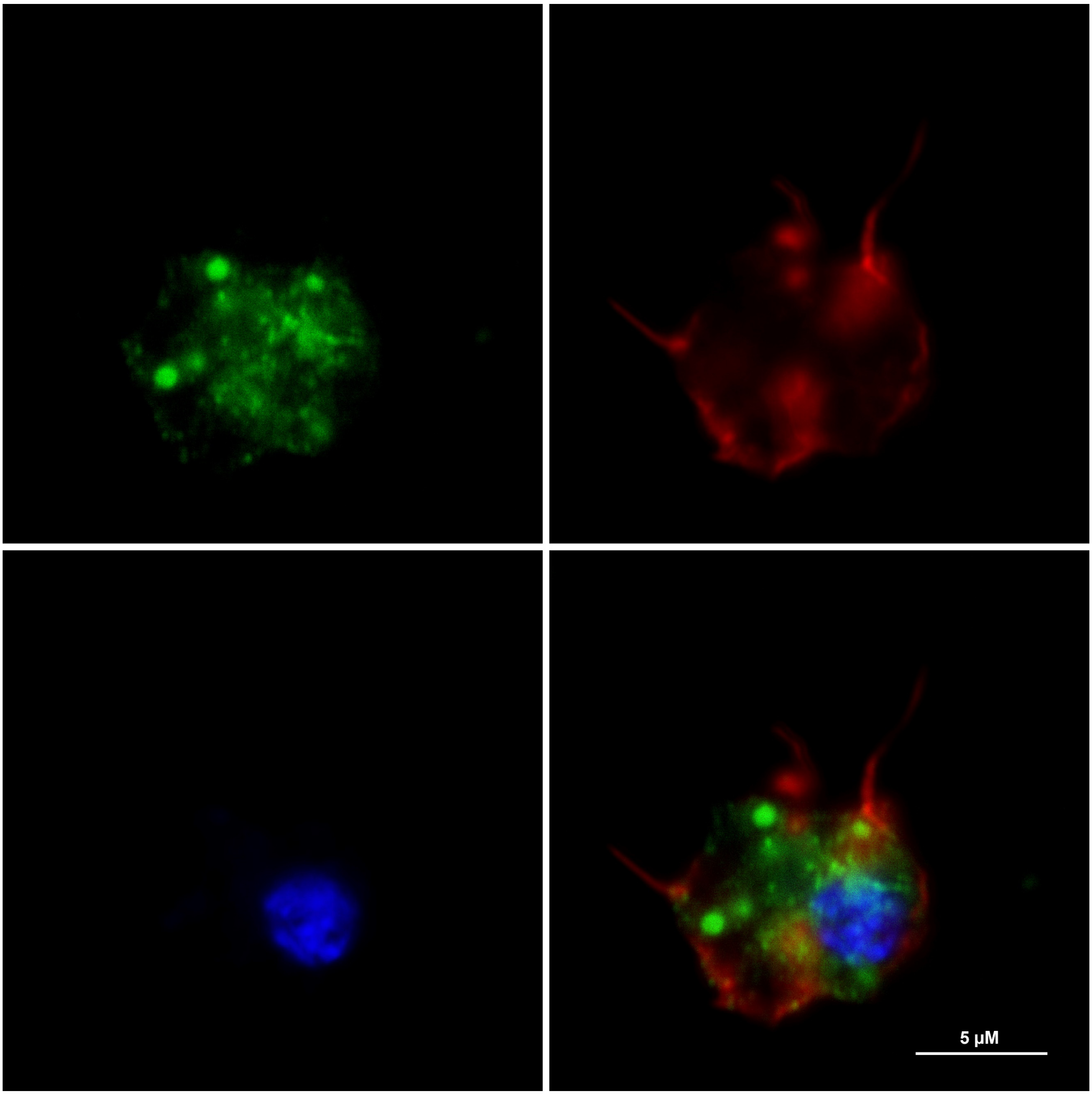

Figure S4I. Confocal microscopy of *H. dujardini* cells triple stained for actin (red), ferritin (green) and chromatin (blue). Scale bar 5 µm.

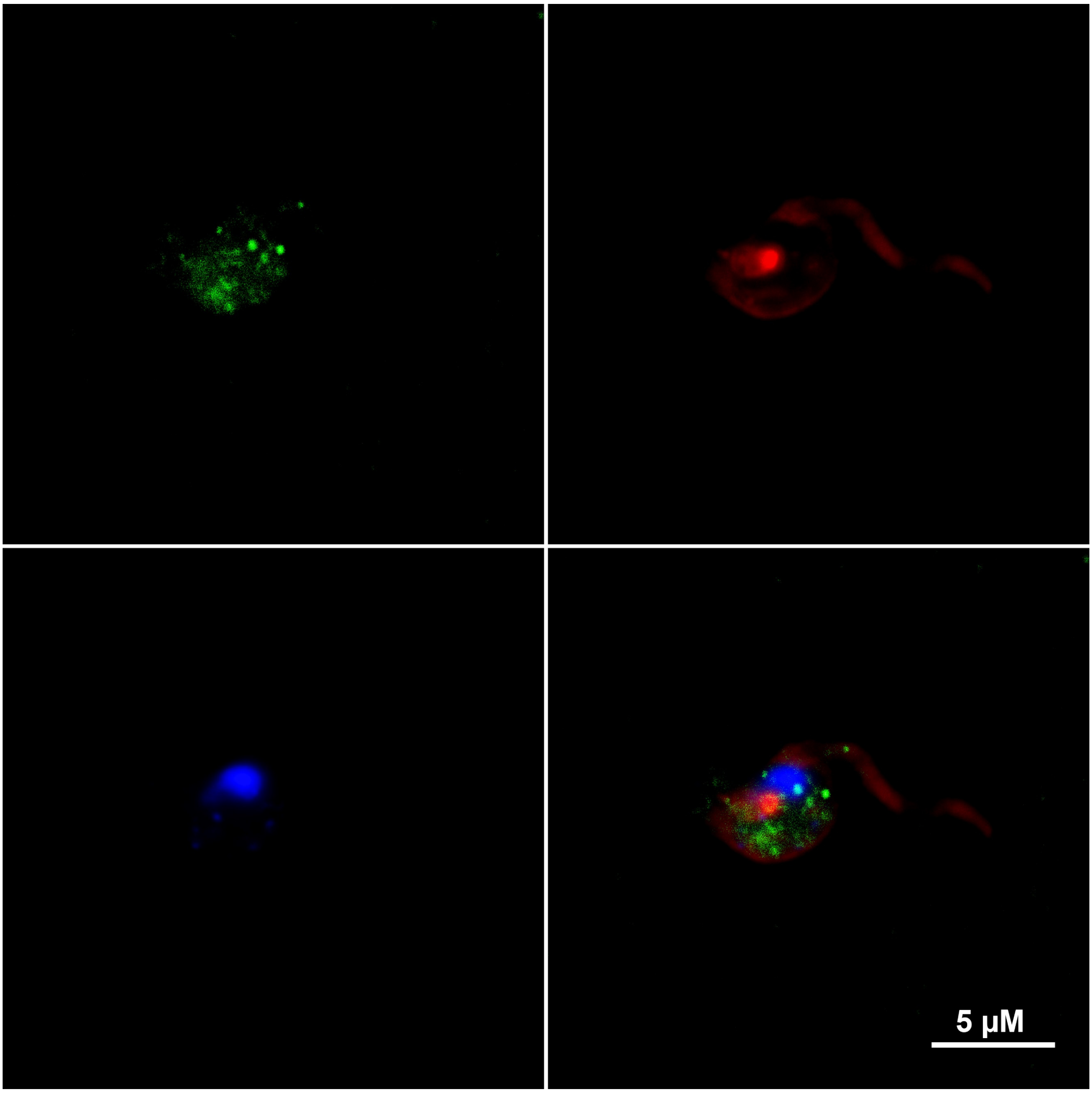

Figure S4J. Confocal microscopy of *H. dujardini* cells triple stained for actin (red), ferritin (green) and chromatin (blue). Scale bar 5 µm.

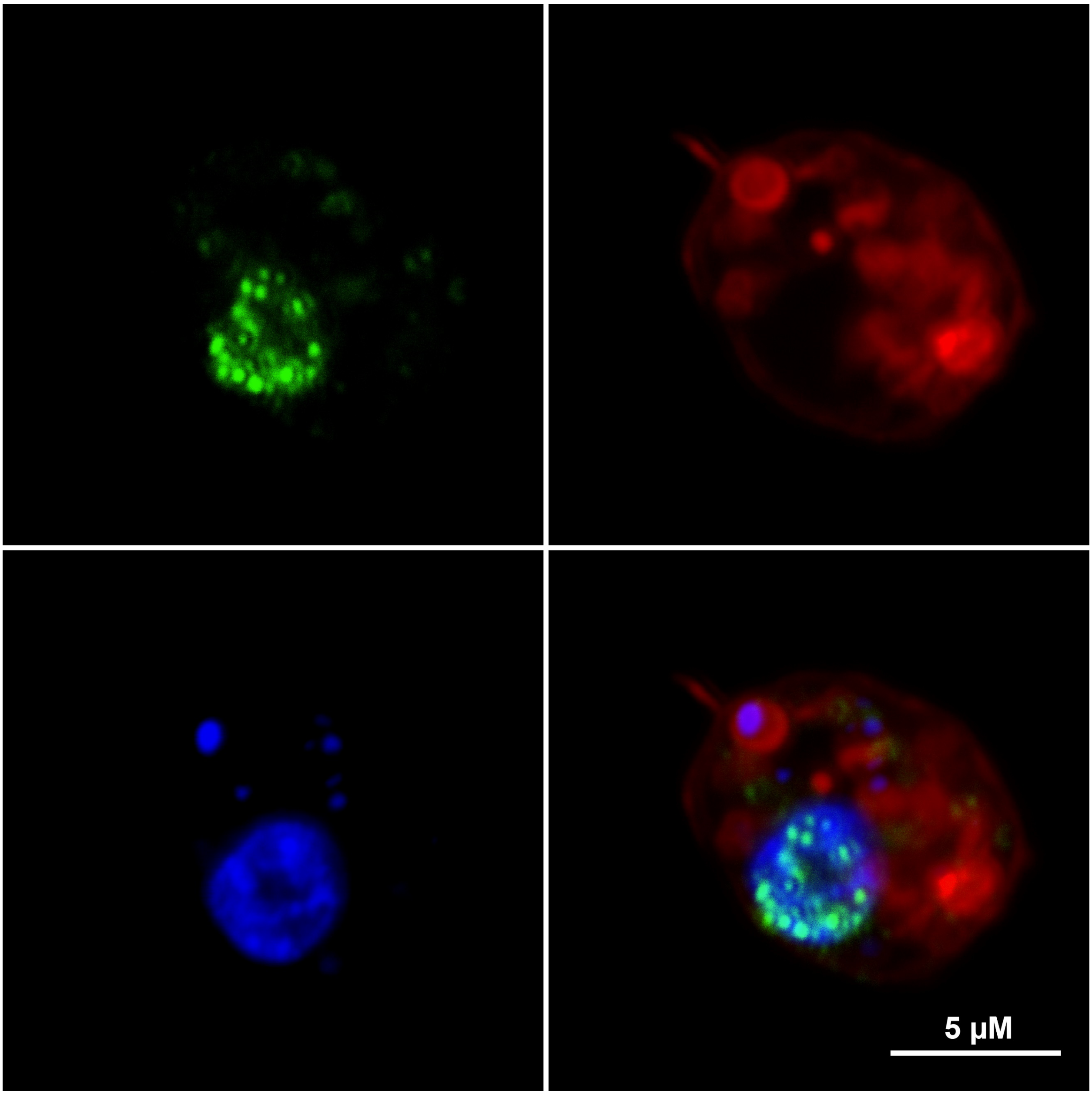

Figure S4K. Confocal microscopy of *H. dujardini* cells triple stained for actin (red), ferritin (green) and chromatin (blue). Scale bar 5 µm.

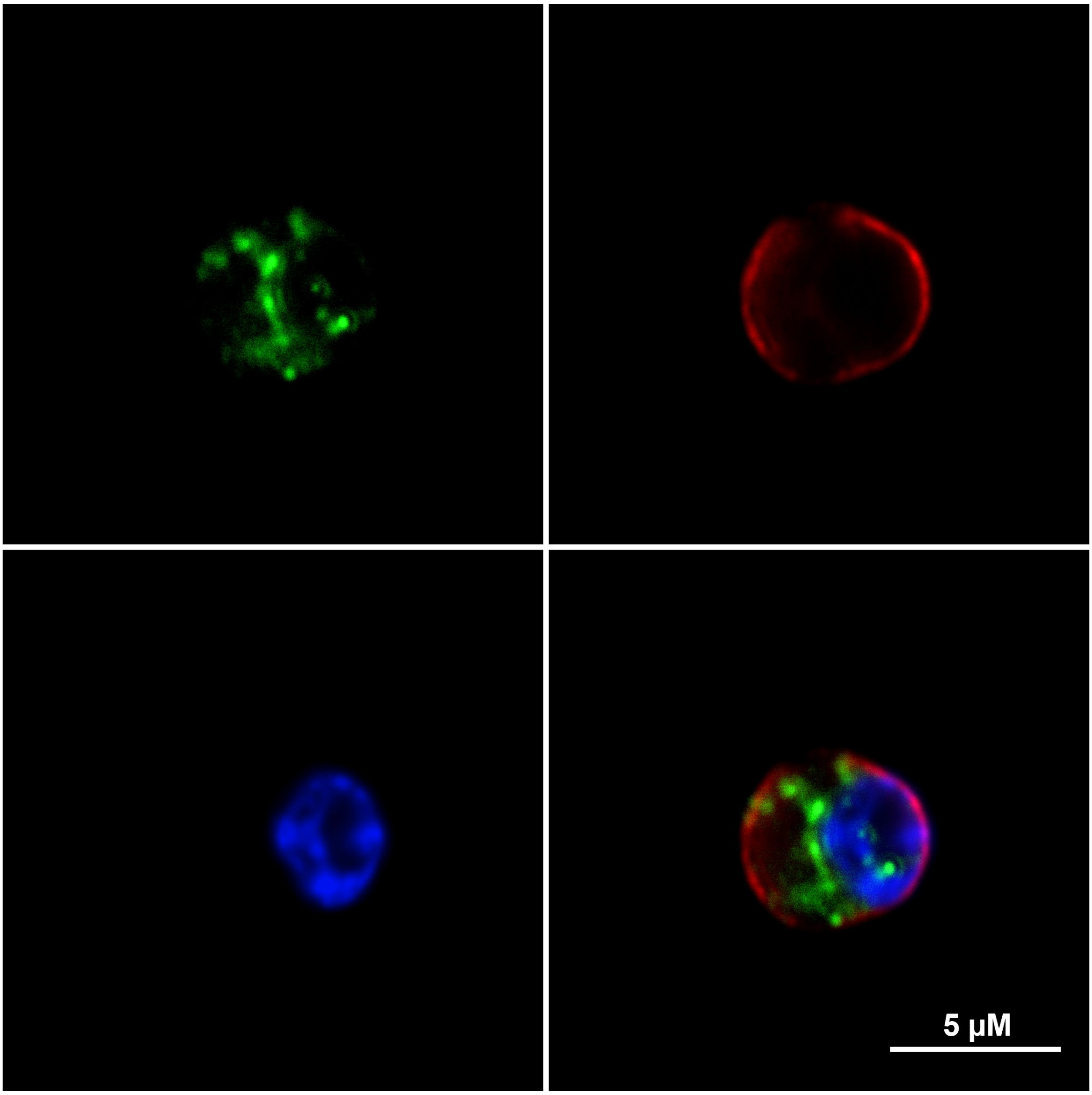

Figure S4L. Confocal microscopy of *H. dujardini* cells triple stained for actin (red), ferritin (green) and chromatin (blue). Scale bar 5 µm.

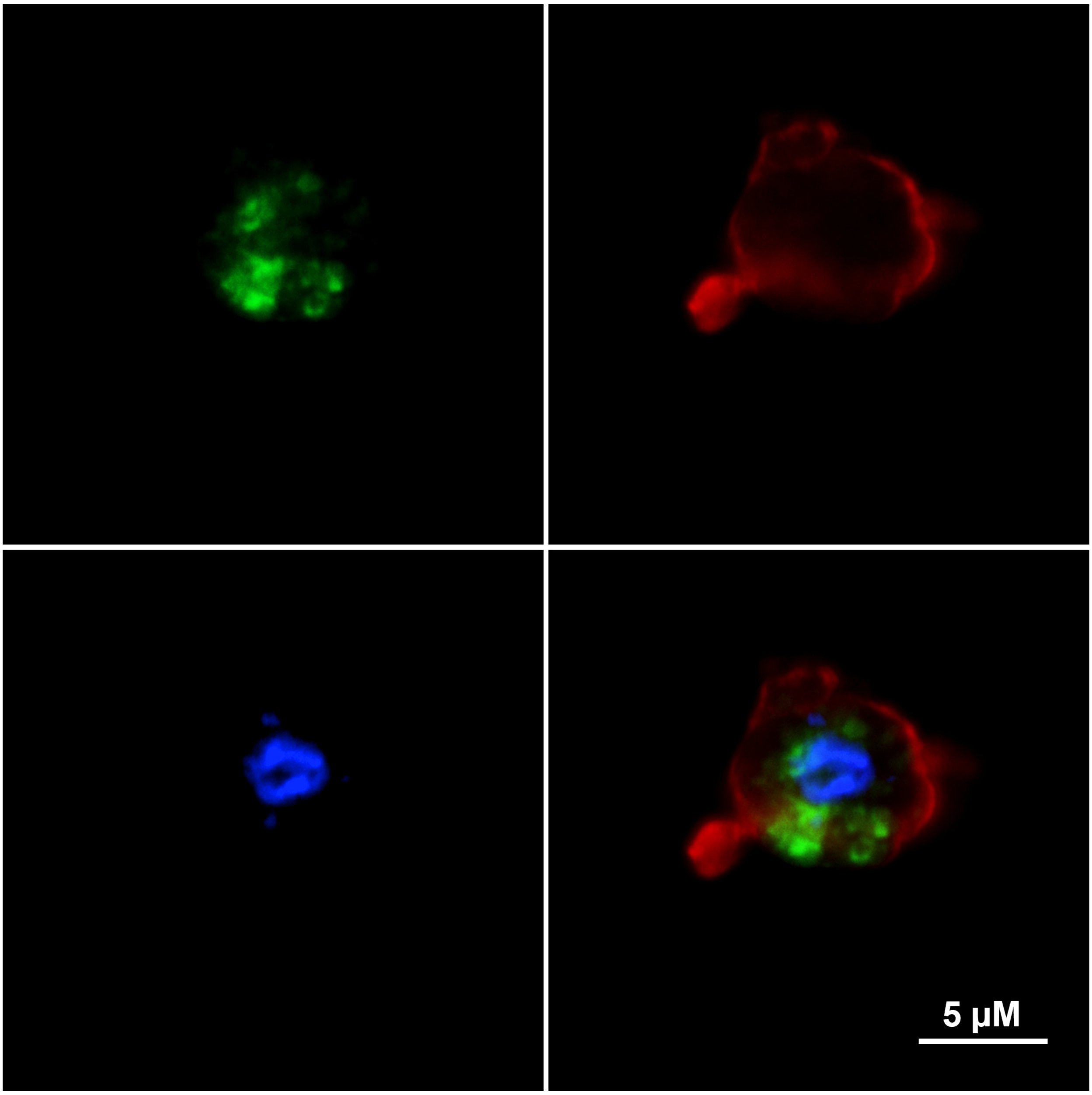

Figure S4M. Confocal microscopy of *H. dujardini* cells triple stained for actin (red), ferritin (green) and chromatin (blue). Scale bar 5 µm.

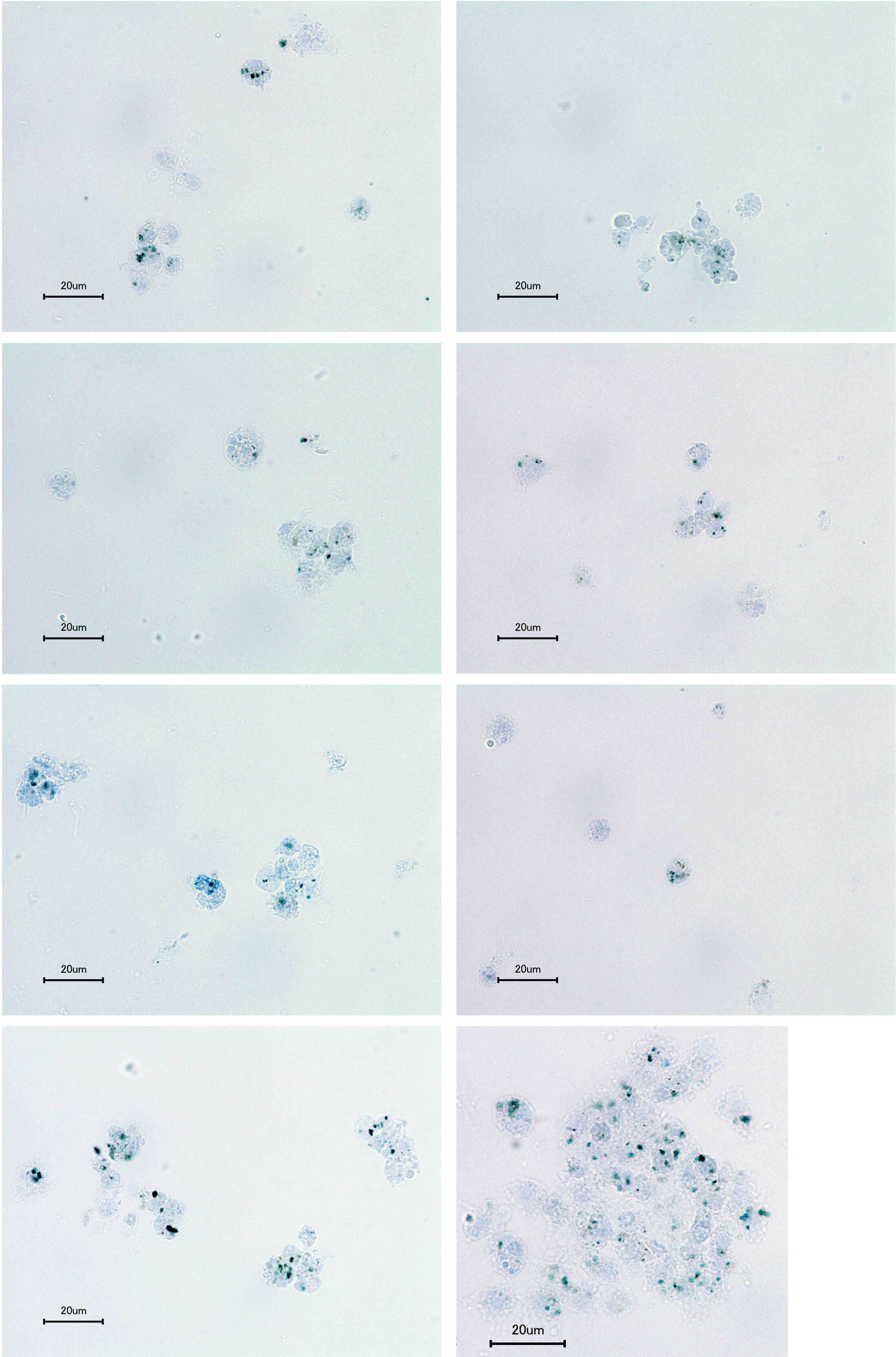

Figure S4N. The ferric complexes detected in *H. dujardini* sponge cells by Prussian blue staining
