# Supplementary material for: Conservative and Atypical Ferritins of Sponges
Source: Int J Mol Sci. 2021 Aug 11;22(16):8635. doi: 10.3390/ijms22168635 (PMC8395497; doi:10.3390/ijms22168635)
Supplement: Supplementary file 1 [file ijms-22-08635-s001.zip › suppl_figures/Figure_S08. Extended mass-spectrometry results - peptide coverage.pdf]

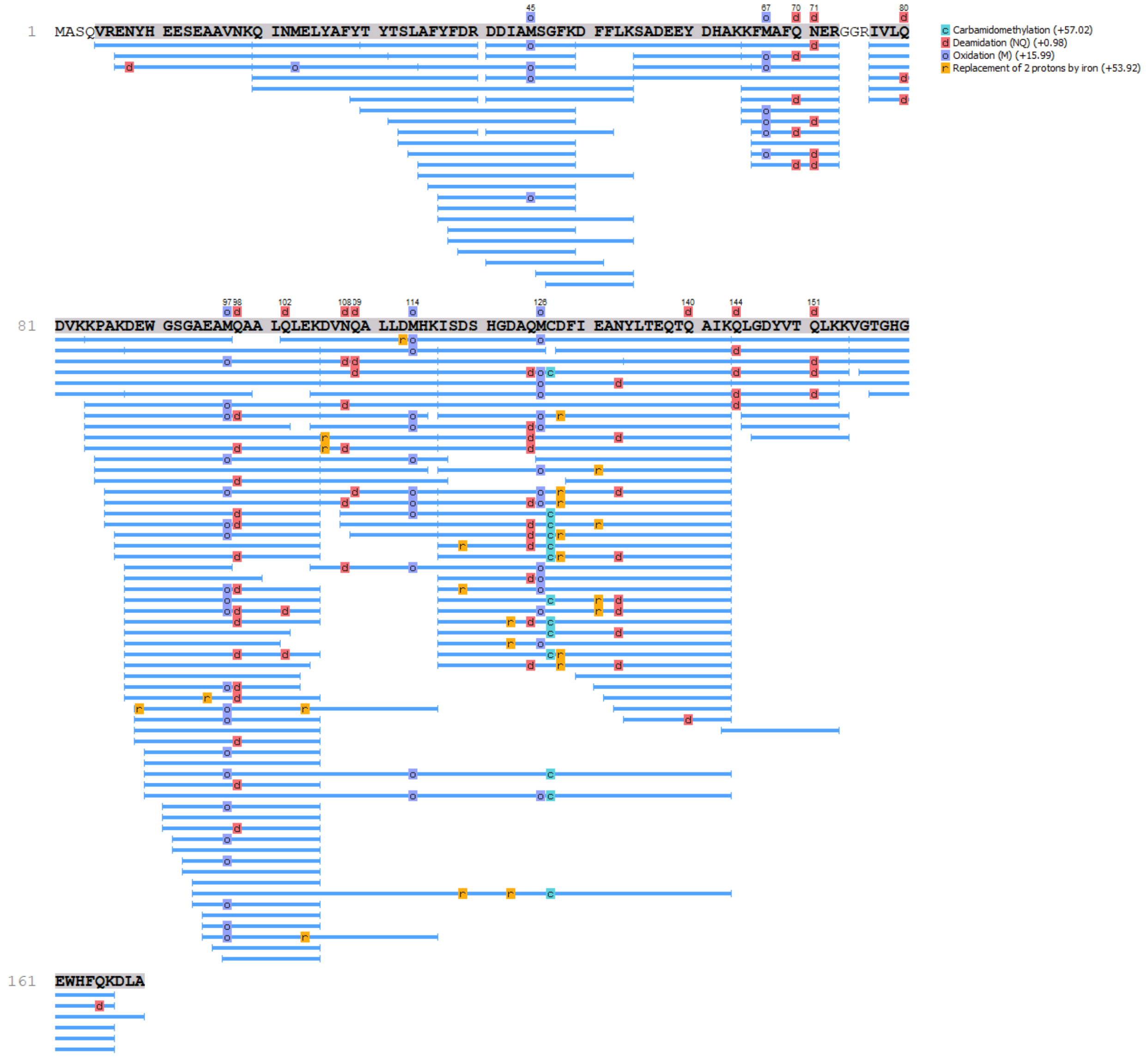

Figure S8A. Extended mass-spectrometry data for HdF1a: peptide coverage

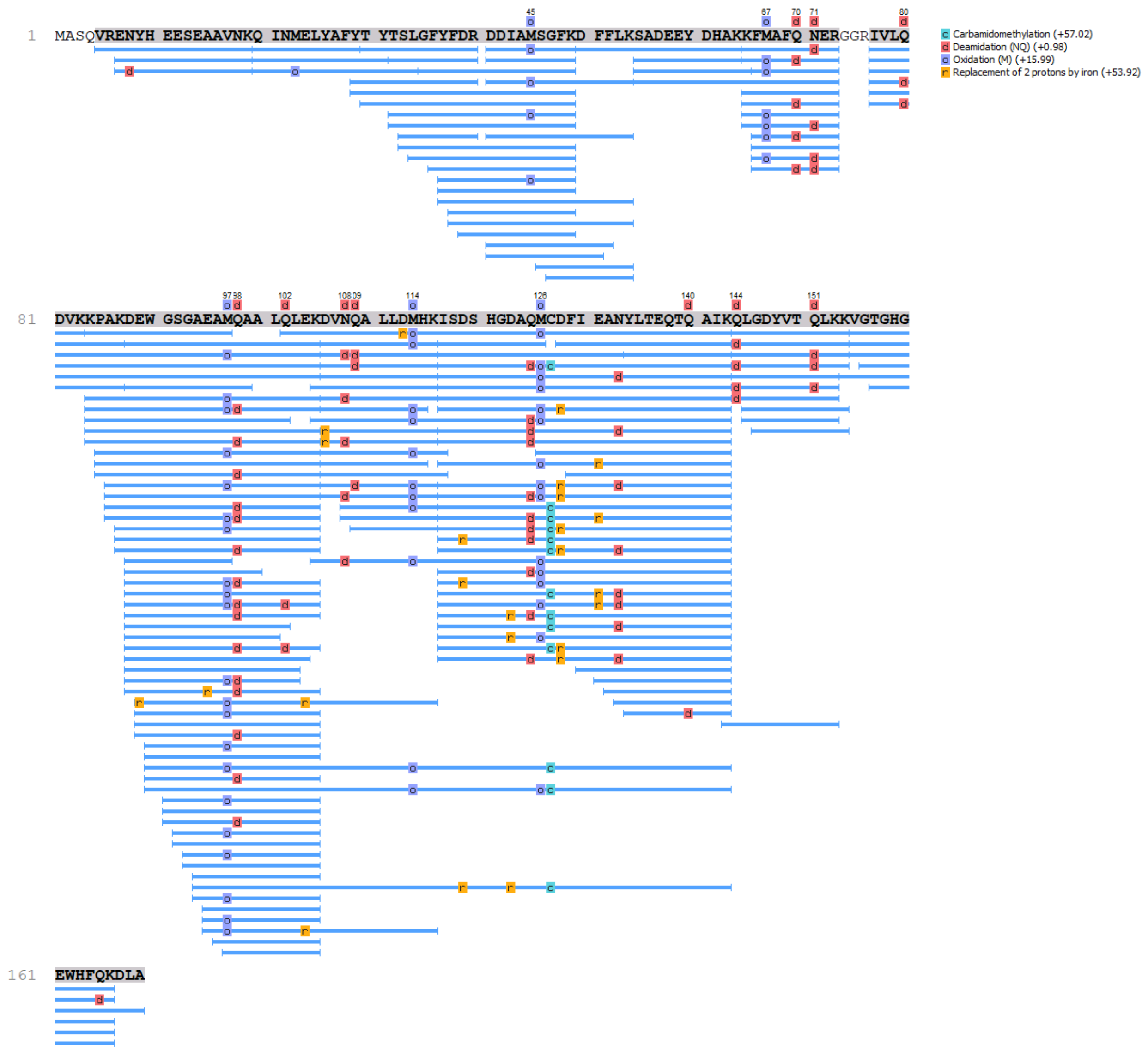

Figure S8B. Extended mass-spectrometry data for HdF1b: peptide coverage

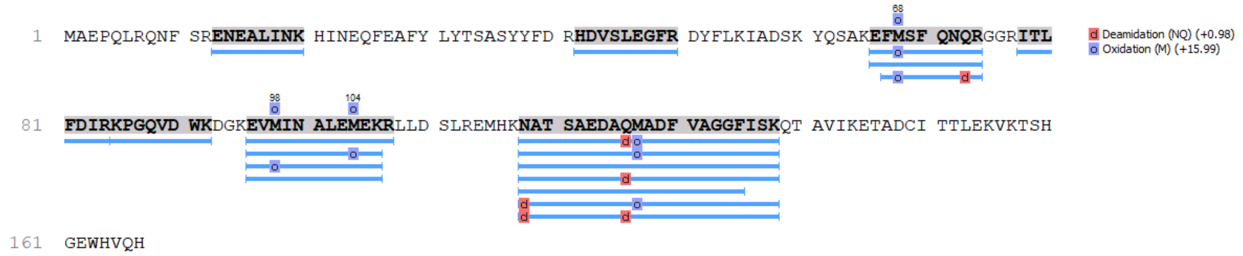

Figure S8C. Extended mass-spectrometry data for HdF2: peptide coverage

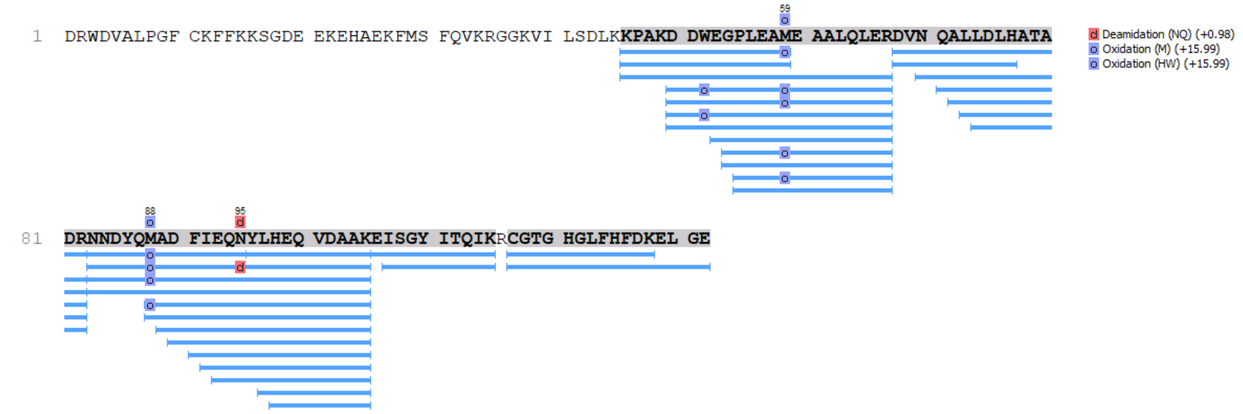

Figure S8D. Extended mass-spectrometry data for HpF1: peptide coverage

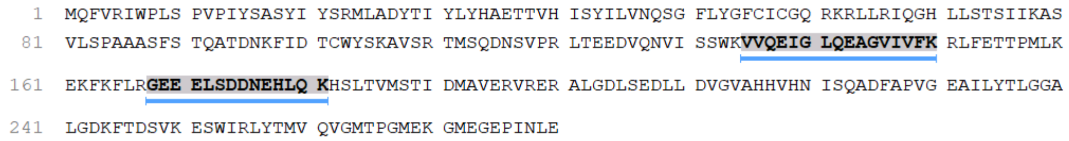

Figure S8E. Extended mass-spectrometry data for H.d. neuroglobin: peptide coverage
