# Supplementary material for: Conservative and Atypical Ferritins of Sponges
Source: Int J Mol Sci. 2021 Aug 11;22(16):8635. doi: 10.3390/ijms22168635 (PMC8395497; doi:10.3390/ijms22168635)
Supplement: Supplementary file 1 [file ijms-22-08635-s001.zip › suppl_figures/Figure_S09. Ferritin complexes native gels.pdf]

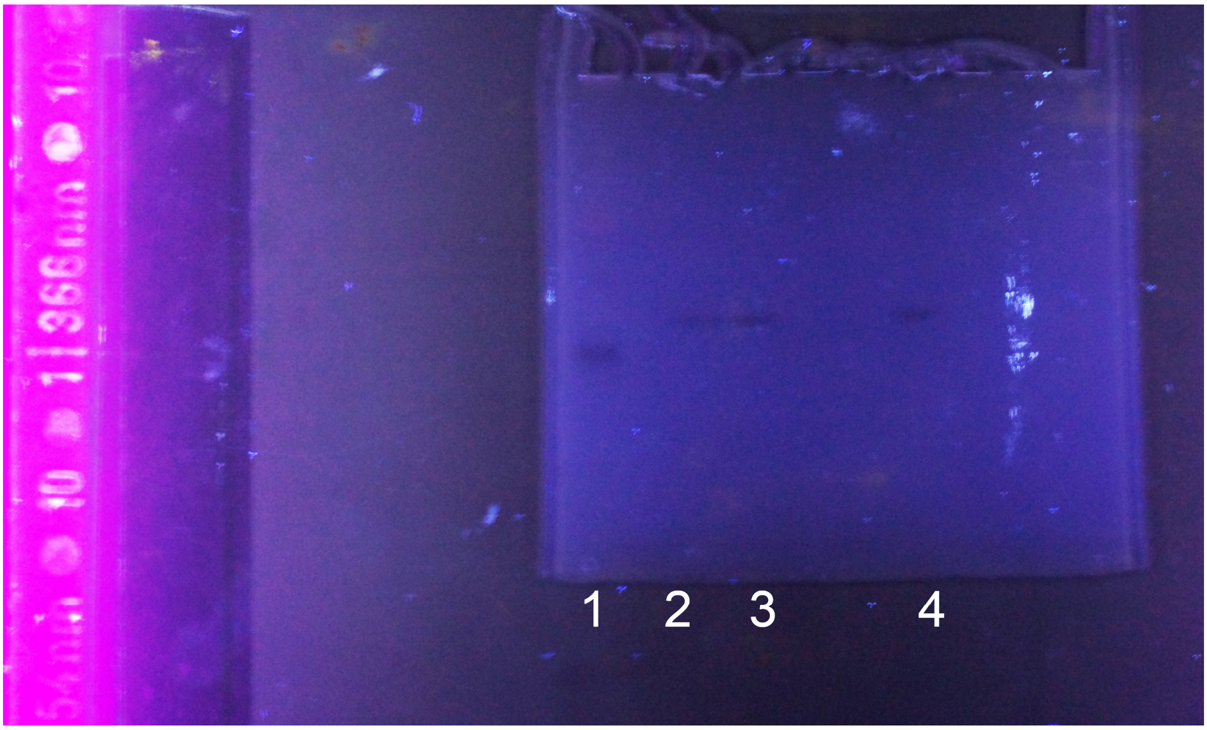

Gradient Native electrophoresis of clarified sponge homogenates: lane 1 – Red – Tyroblobulin (670 kDa); Black –Horse ferritin (440 kDa); lane 2 – Halichondria panicea body, lane 3 - Halichondria panicea cells; lane 4 – Halisarca dujardini body

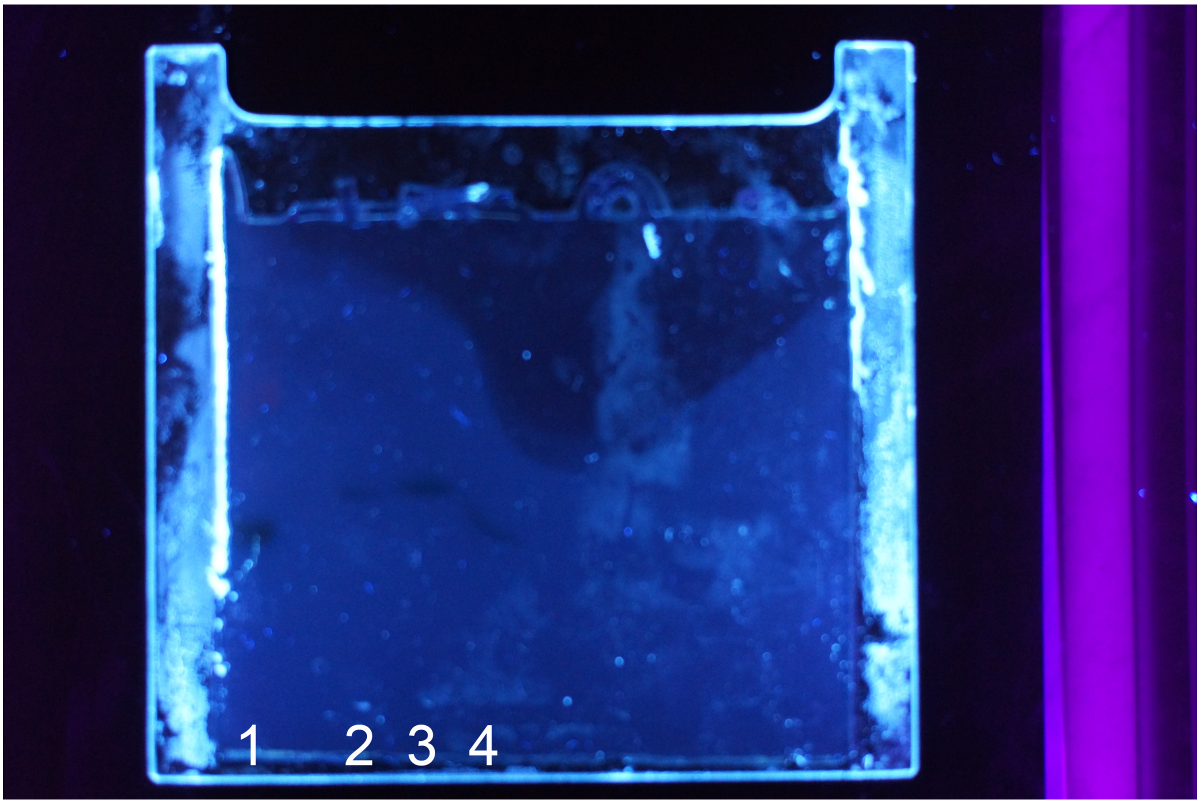

Gradient Native electrophoresis of clarified sponge homogenates: lane 1 – Red –Tyroblobulin (670 kDa); Black –Horse ferritin (440 kDa); lane 2 – Halichondria panicea body, lane 3 - Halichondria panicea cells; lane 4 – Halisarca dujardini body

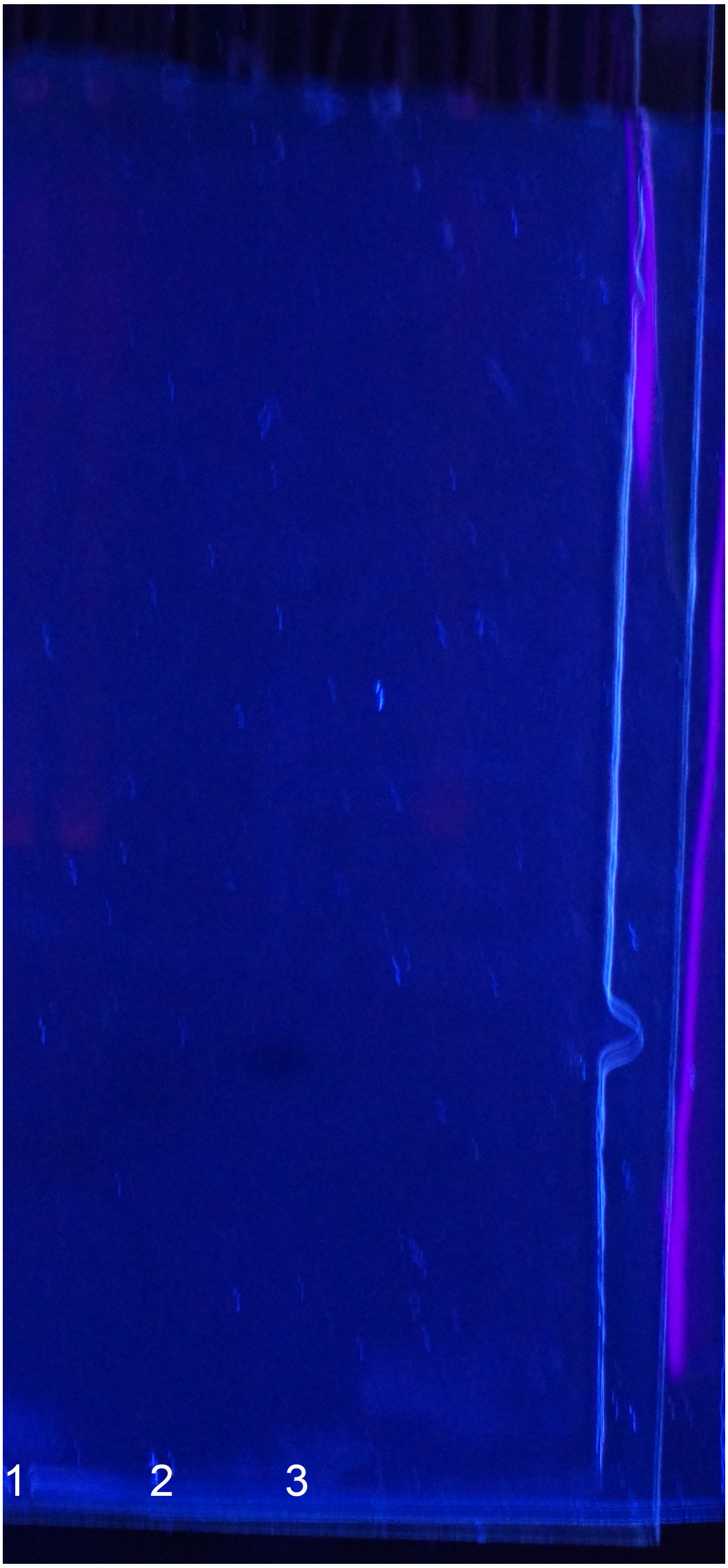

Gradient Native electrophoresis of clarified sponge homogenates: lane 1 – Red –Tyroblobulin (670 kDa); Black –Horse ferritin (440 kDa); lane 2 – Halisarca dujardini body cells and lane 3 – Halichondria panicea body cells (Summer).

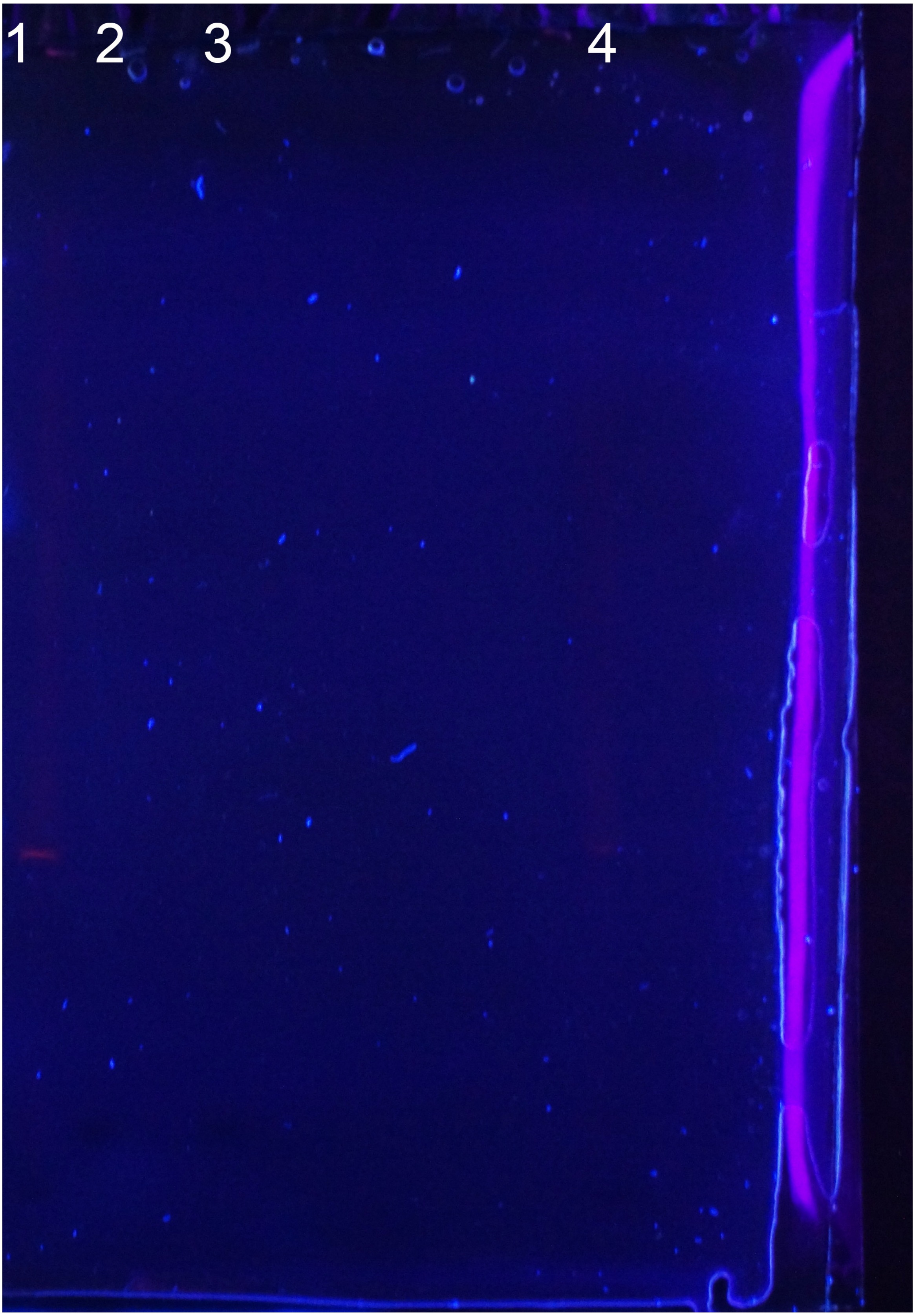

Gradient Native electrophoresis of clarified sponge homogenates: lanes 1, 4 – Red –Tyroblobulin (670 kDa); Black –Horse ferritin (440 kDa); lane 2 – Halisarca dujardini body and lane 3 – Halisarca dujardini body cells (Autumn).

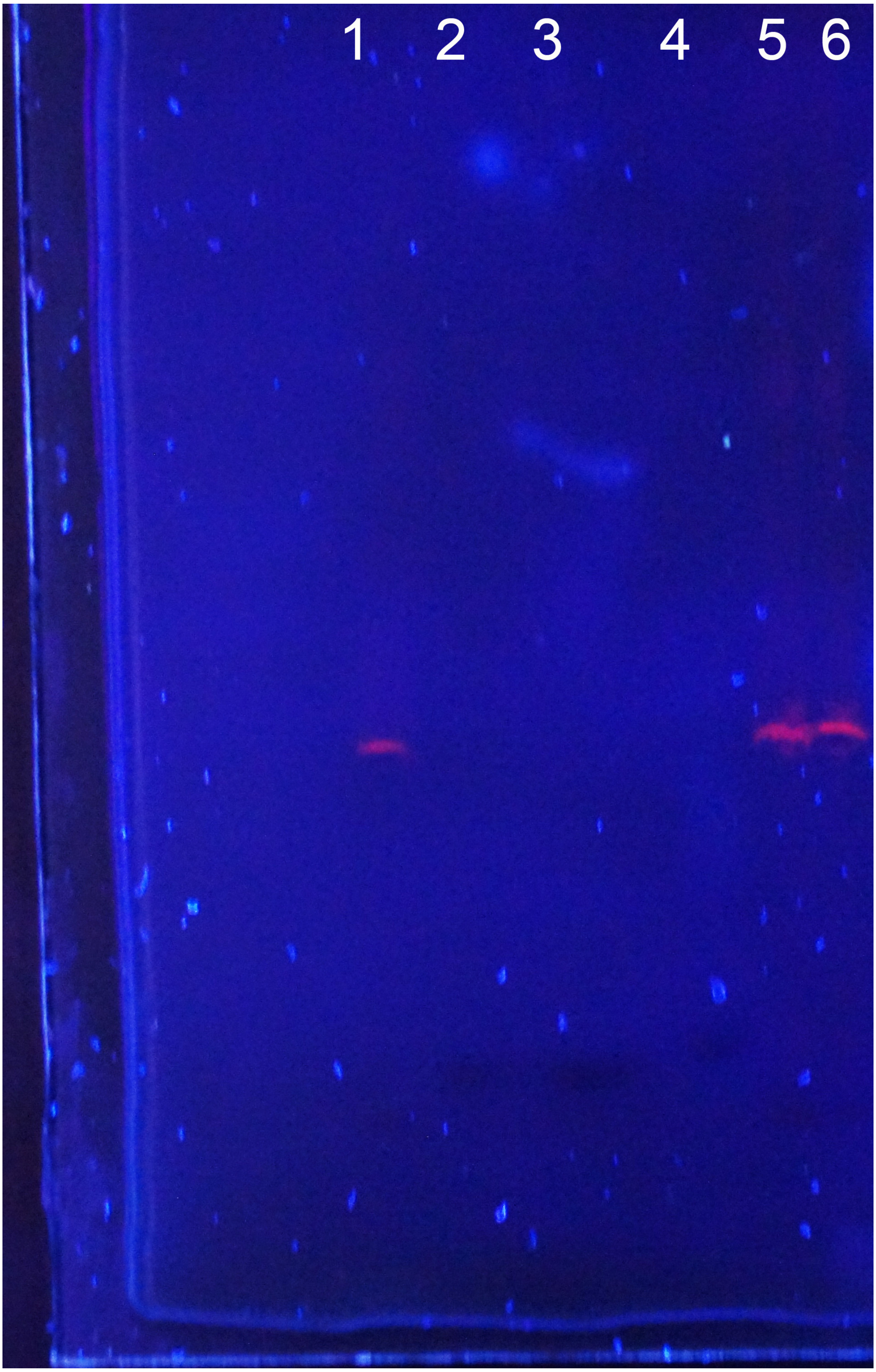

Gradient Native electrophoresis of clarified sponge homogenates: lanes 1, 5, 6 – Red – Tyroblobulin (670 kDa); Black – Horse ferritin (440 kDa); lane 2 – Halisarca dujardini body and lane 3 – Halisarca dujardini body cells and lane 4 – Halichondria panicea body cells (Autumn).

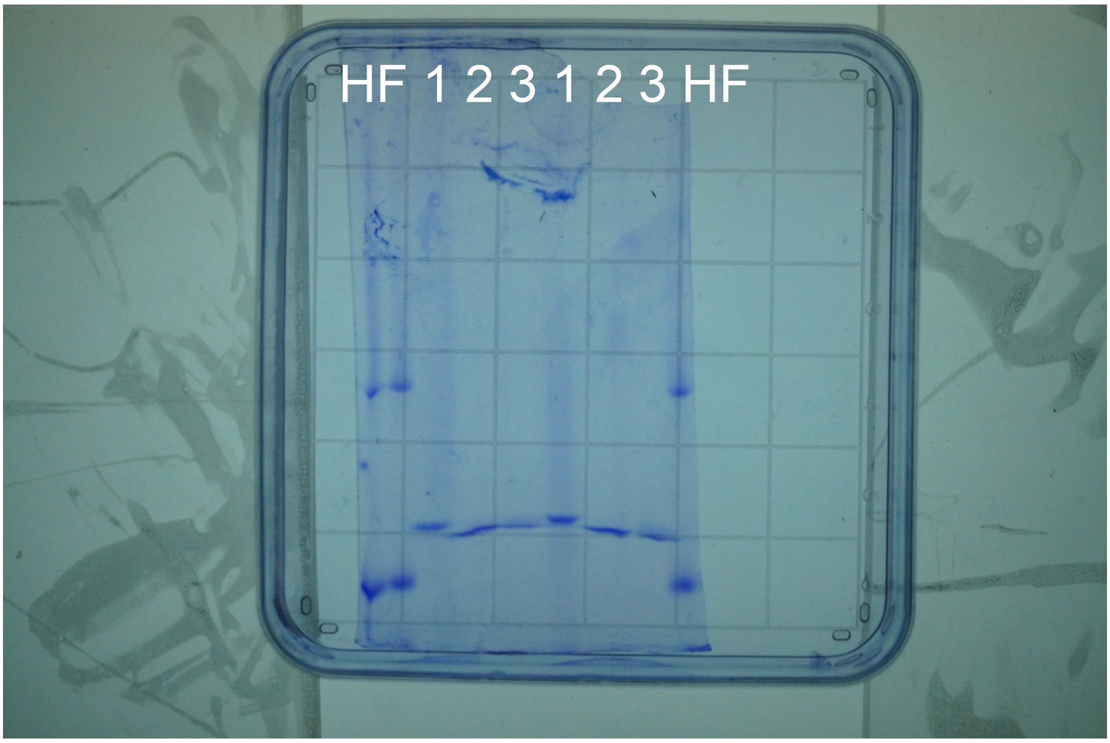

Gradient Native electrophoresis of clarified sponge homogenates: HF – (440 kDa); lane 1 - Halichondria panicea body cells; lane 2 – Halisarca dujardini body and lane 3 – Halichondria dujardini body cells (Summer).

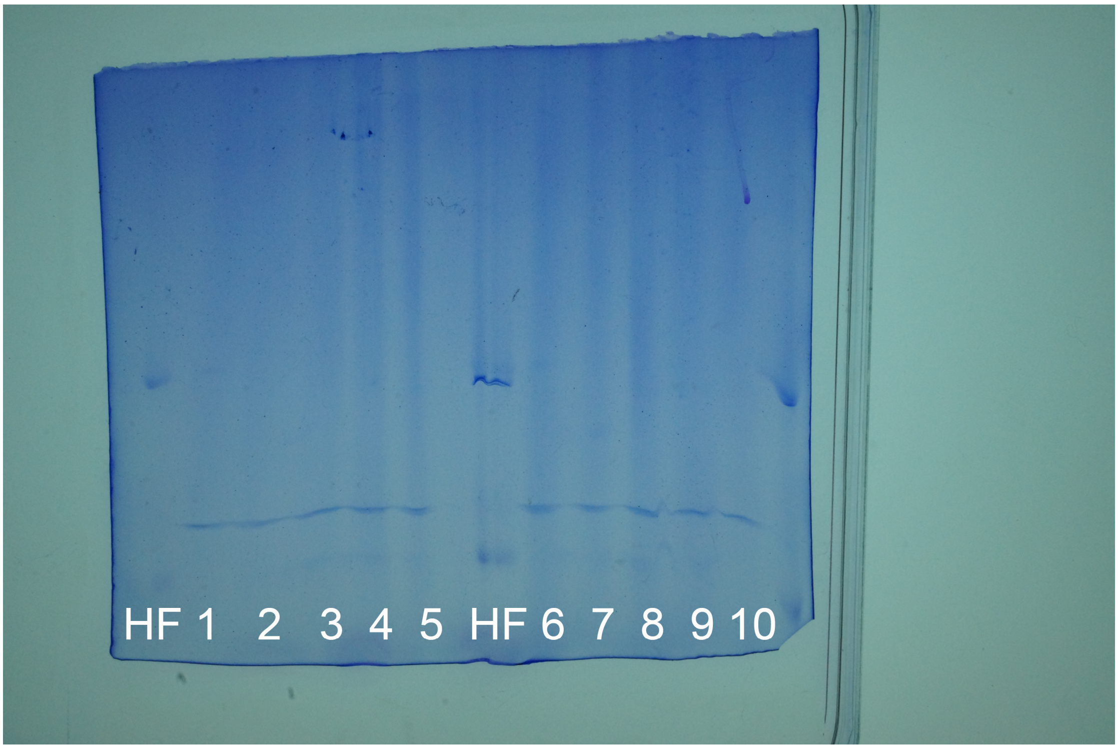

Gradient Native electrophoresis of clarified sponge homogenates: Native electrophoresis of clarified sponge homogenates: HF – (440 kDa); lanes 1, 2, 3, 4, 5, 6, 7, 8, 9, 10 - Halisarca dujardini cells

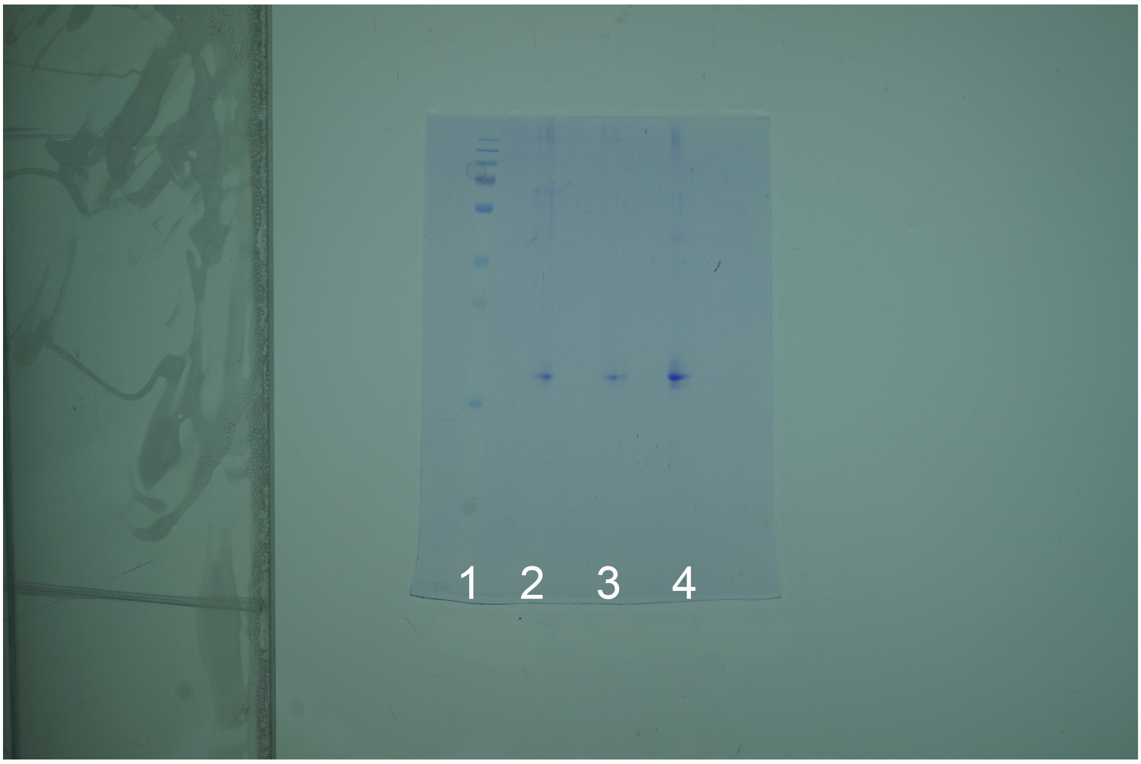

13 % SDS electrophoresis of clarified sponge homogenates:1 – Protein ladder; 2, 3, 4 – Halisarca dujardini body cell ferritin (3, 2, 5 lanes from Native gel)

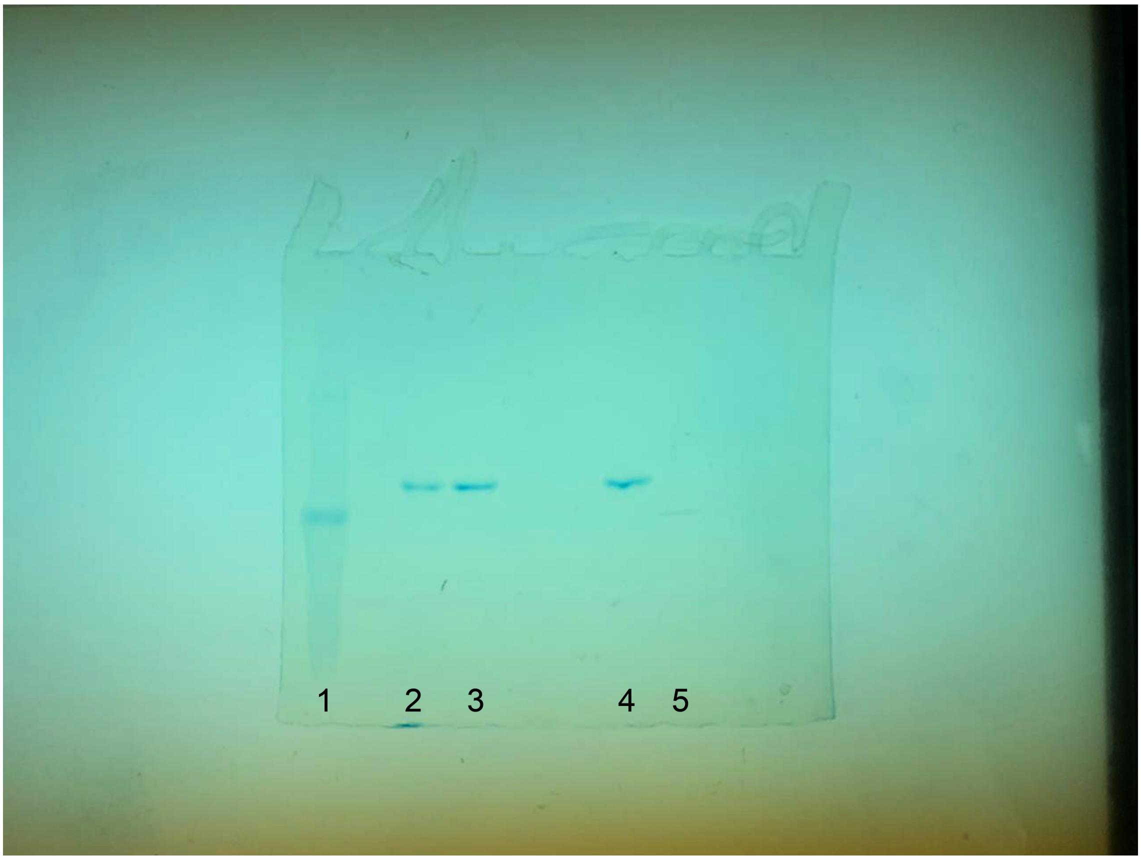

Gradient Native electrophoresis and Prussian blue staining of clarified sponge homogenates: lane 1 – Horse ferritin, lanes 2, 3 – Halichondria panicea bodies, lane 3 – Halichondria panicea body cells; lane 4 – Halisarca dujardini body cells.

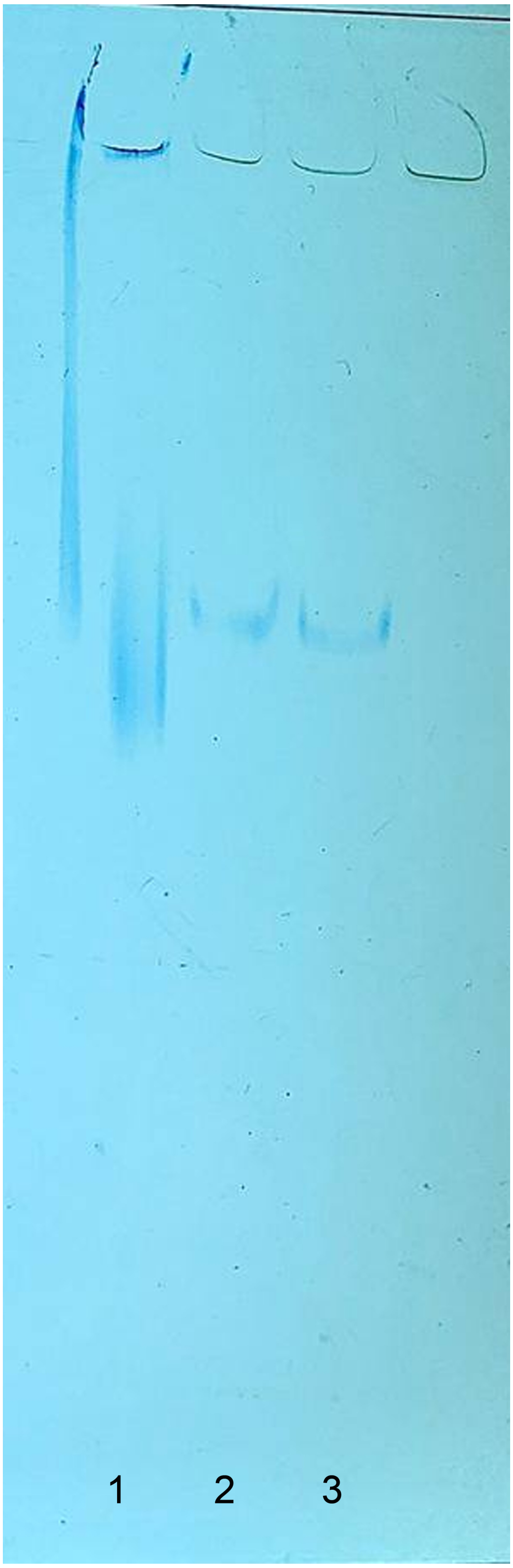

Native electrophoresis and Prussian blue staining of clarified sponge homogenates: lane 1 – Horse ferritin, lane 2 – Halichondria panicea body cells, lane 3 – Halisarca dujardini body cells.

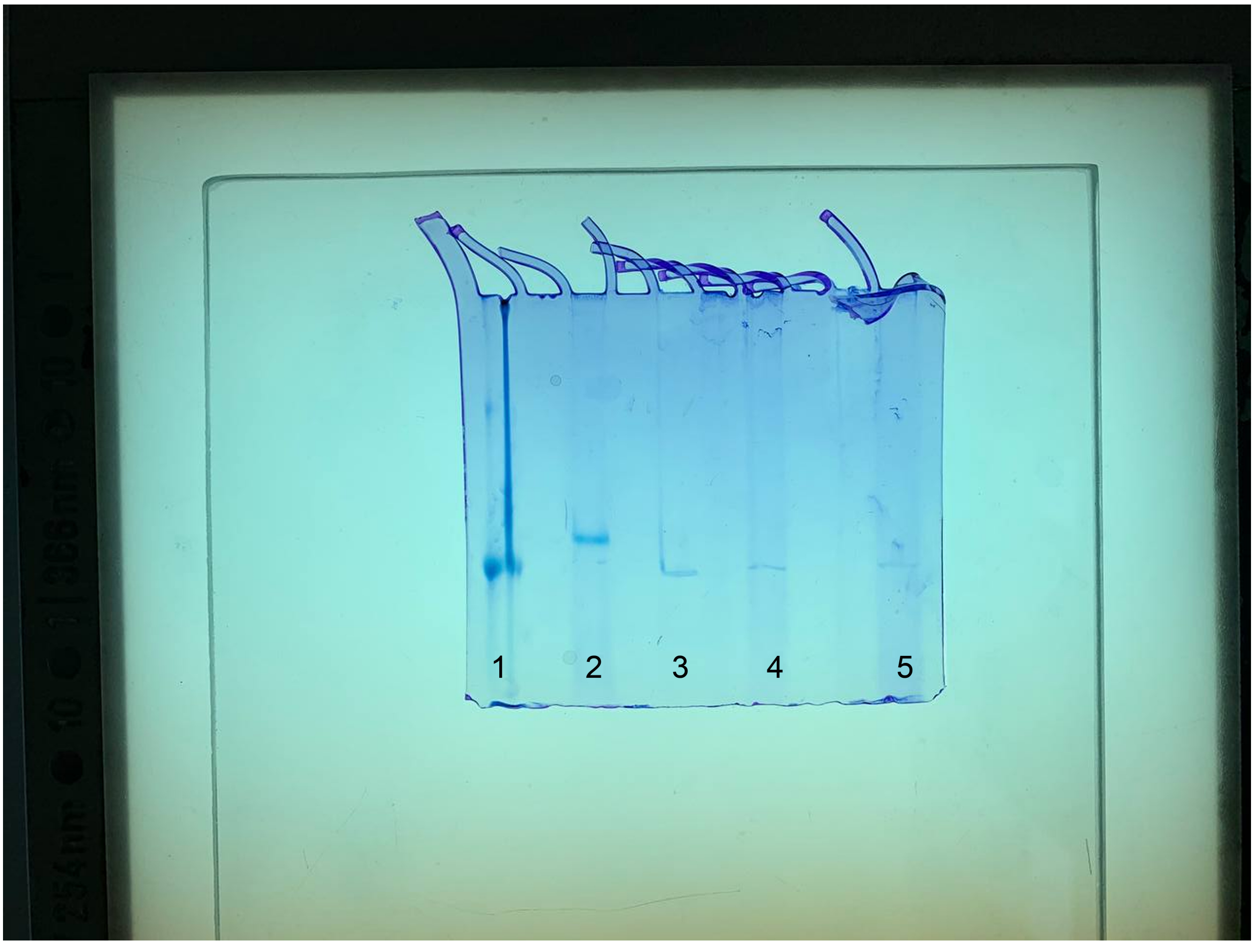

Gradient Native electrophoresis and Prussian blue staining of clarified sponge homogenates: lane 1 – Horse ferritin, lane 2 – Halichondria panicea bodies, lane 3 – Halisarca dujardini body; lane 4 – Halisarca dujardini body cells, 5 – Halisarca dujardini cell aggregates.
