# Supplementary figures and images for: Conservative and Atypical Ferritins of Sponges
Source: Int J Mol Sci. 2021 Aug 11;22(16):8635. doi: 10.3390/ijms22168635 (PMC8395497; doi:10.3390/ijms22168635)

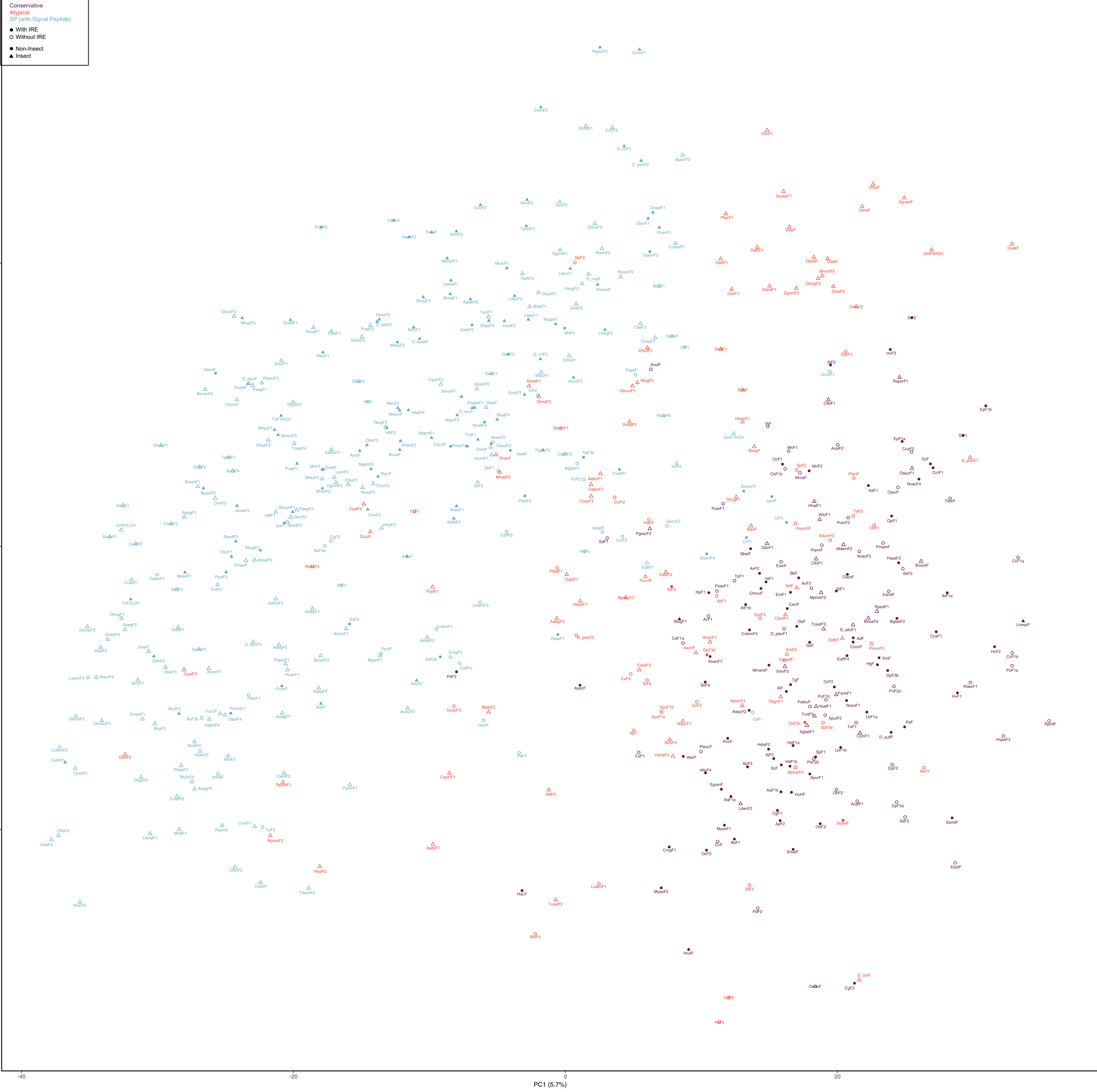

Supplement: Supplementary file 1 [file ijms-22-08635-s001.zip › suppl_figures/Figure_S02. PCA on a set of animal ferritins built using 474 sequence features selected by unsupervised algorithms (large version).pdf]

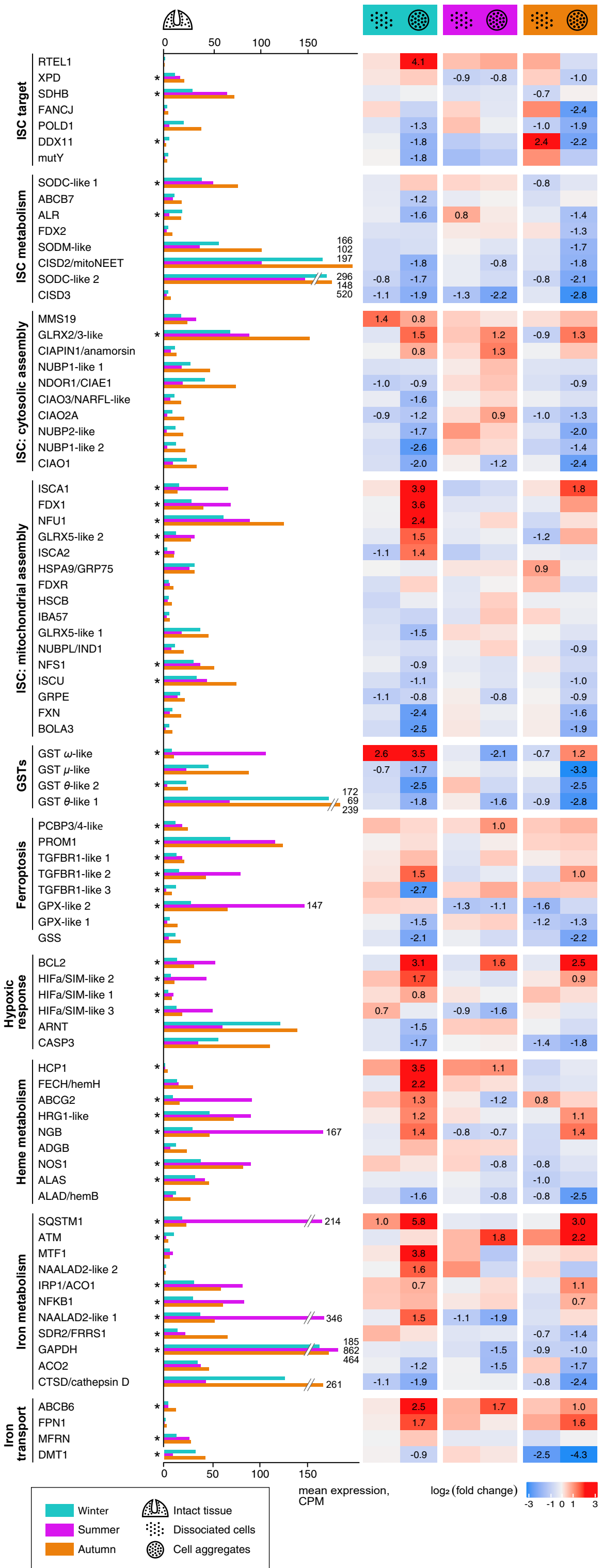

Supplement: Supplementary file 1 [file ijms-22-08635-s001.zip › suppl_figures/Figure_S05. Heatmap of expression levels of iron metabolic proteins of H.dujardini during the reaggregation experiment.pdf]

# Color Key

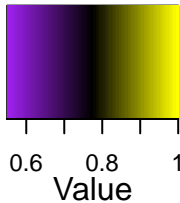

sample correlation matrix  
hd\_se\_3seasons\_24h\_RSEM.gene.counts.tsv.minRow10.CPM.log2

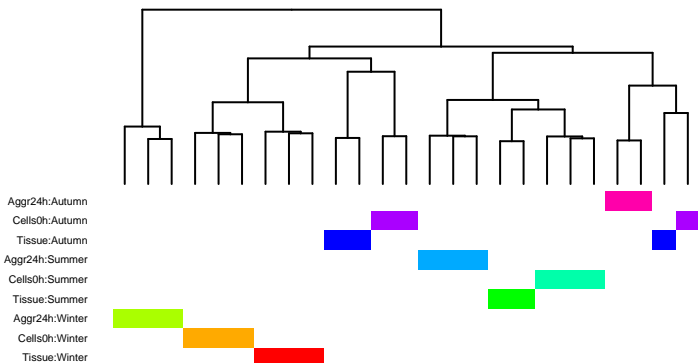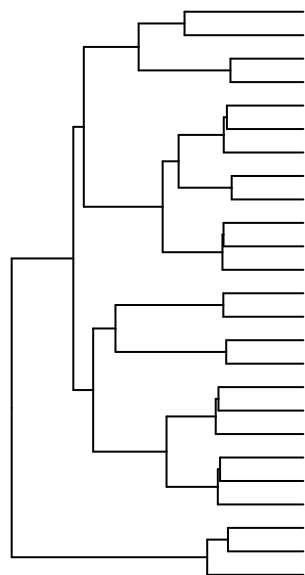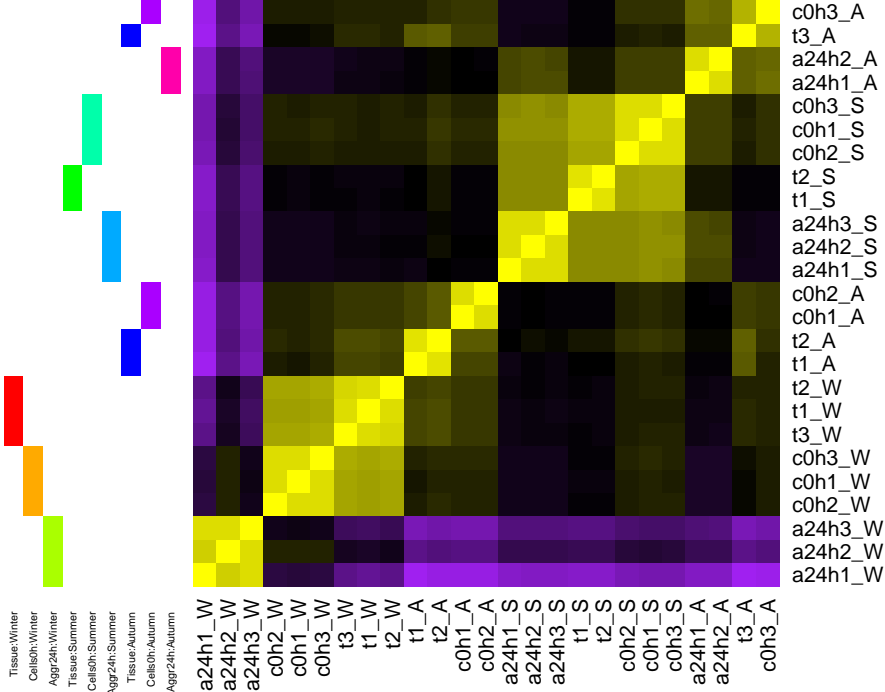

Supplement: Supplementary file 1 [file ijms-22-08635-s001.zip › suppl_figures/Figure_S06. Expression correlation of RNA-Seq samples of H.dujardini.pdf]

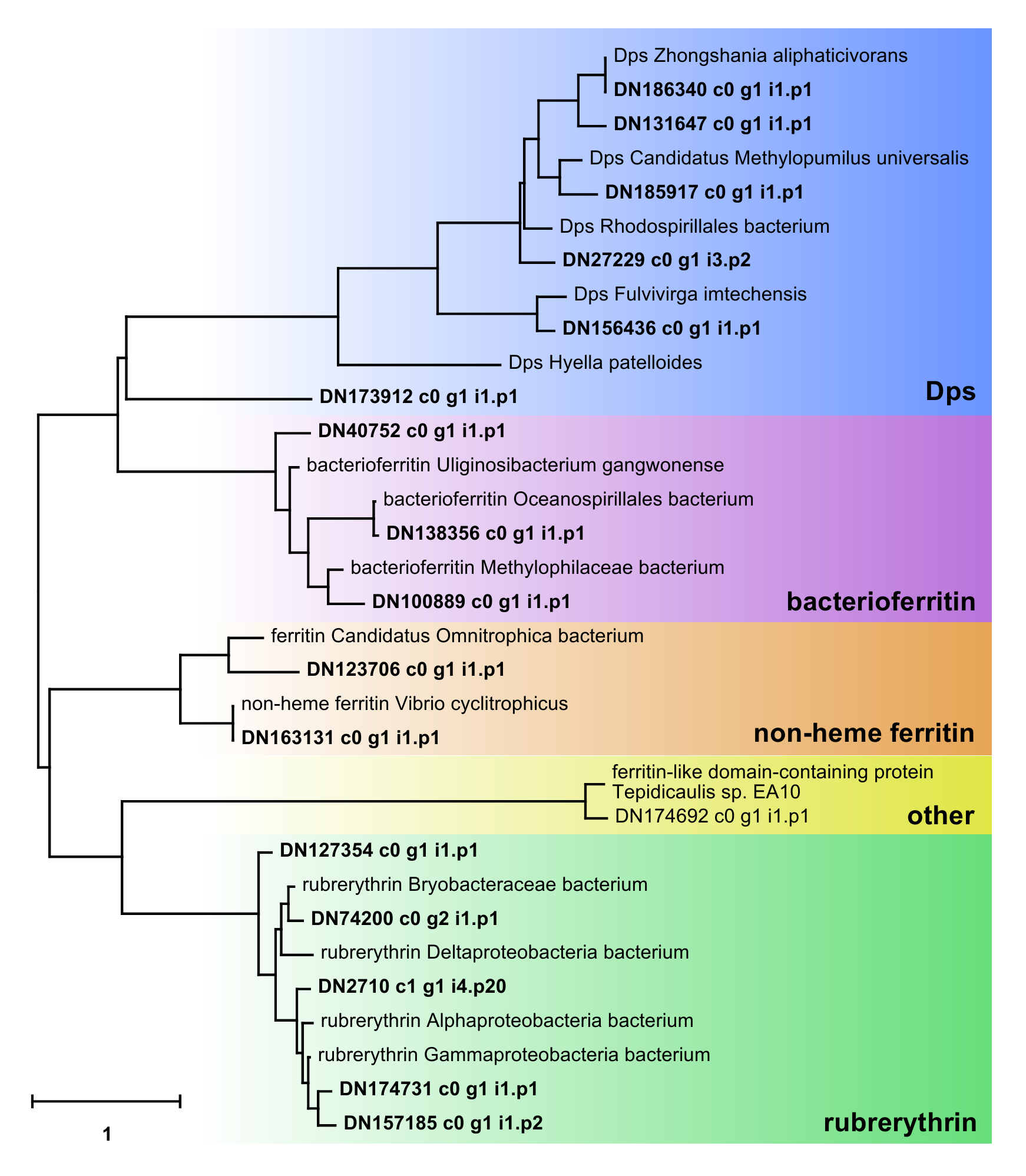

Supplement: Supplementary file 1 [file ijms-22-08635-s001.zip › suppl_figures/Figure_S10. Phylogenetic tree of bacterial ferritin superfamily members.png]
